# Hypnosis for anaesthetists: a systematic review and meta‐analyses

**DOI:** 10.1111/anae.70013

**Published:** 2025-09-24

**Authors:** Marie José Lahoud, Samuel Tell Gurary, Nadia Elia

**Affiliations:** ^1^ Division of Anaesthesiology, Department of Acute Care Medicine Geneva University Hospitals Geneva Switzerland; ^2^ Department of Anaesthesiology, Pharmacology, Intensive Care and Emergency Medicine, Faculty of Medicine University of Geneva Geneva Switzerland; ^3^ Faculty of Medicine, University of Geneva Geneva Switzerland

**Keywords:** anaesthesia, hypnosis, meta‐analyses, peri‐operative care, surgery

## Abstract

**Introduction:**

Therapeutic hypnosis appears to offer psychological and physiological benefits in various medical fields, but despite increasing interest, its value for anaesthesia remains inconclusive.

**Methods:**

We searched for studies of any design in which hypnosis was used for any intervention requiring the presence of an anaesthetist, alone or in combination with any type of anaesthesia, on children and adults. Meta‐analyses using random‐effects models were stratified on hypnosis timing, when three or more randomised controlled trials reported on a similar outcome. Additional analyses were performed adding data derived from non‐randomised controlled studies. The primary outcome was the use of hypnotics and opioids during the intervention. Secondary outcomes included all outcomes related to pain, anxiety or adverse events.

**Results:**

We identified 142 studies that included 9238 patients (8319 adults, 919 children). Pre‐intervention hypnosis decreased post‐intervention visual analogue scale pain score (mean difference ‐0.88 cm, 95%CI ‐1.72 to ‐0.05) and anxiety (standardised mean difference ‐0.76, 95%CI ‐1.14 to ‐0.38). Per‐intervention hypnosis decreased visual analogue scale pain intensity during the intervention (mean difference ‐1.14 cm, 95%CI ‐1.86 to ‐0.41) without impacting on post‐intervention pain; decreased post‐intervention anxiety (standardised mean difference ‐0.44, 95%CI ‐0.75 to ‐0.13); and lowered the risk of postoperative nausea and vomiting (risk ratio 0.43, 95%CI 0.25–0.74). Adding non‐randomised controlled studies did not alter these results substantially. Evidence of the impact of pre‐ or per‐intervention hypnosis on other outcomes, or of post‐intervention hypnosis on any outcome, was lacking.

**Discussion:**

Hypnosis may help reduce anxiety, alleviate pain during and after a procedure and lower the incidence of postoperative nausea and vomiting. However, despite the inclusion of more than 9000 patients in studies examining the use of hypnosis for anaesthesia, its impact on most outcomes remains unknown.

## Plain Language Summary

Hypnosis is sometimes used in medicine and may help people feel better in their mind and body. Some doctors are interested in using hypnosis during surgery, but we still don't know for sure how helpful it really is. To learn more, we looked at studies where hypnosis was used in medical procedures that needed an anaesthetist, a doctor who helps people sleep or stay calm during surgery. These studies included both adults and children. The main thing we checked was how much medicine, like sleeping drugs or painkillers, people needed. We also looked at pain, anxiety and side effects. In total, we found 142 studies with 9238 people, including 8319 adults and 919 children. When hypnosis was used before the procedure, people later had less pain and felt less worried. When hypnosis was used during the procedure, people felt less pain at that time, had less anxiety afterwards and were less likely to feel sick or vomit after surgery. Using results from less strict studies did not change these findings much. However, there wasn't enough proof about what happens if hypnosis is used after the procedure. Overall, hypnosis might help people feel calmer, reduce pain and lower the chance of feeling sick after surgery. But even though many studies have been done, we still don't know everything about how hypnosis affects patients during surgery.

## Introduction

Therapeutic hypnosis has gained attention as a complementary approach to conventional treatments. It is characterised by a trance‐like mental state induced through verbal and non‐verbal communication between the hypnotherapist and the patient [1]. This state reduces peripheral awareness and heightens responsiveness to suggestion and is thought to offer psychological and physiological benefits [2].

In anaesthesia, hypnosis is sometimes used as the sole anaesthetic for minor surgeries, in conjunction with light sedation, or as an adjuvant technique during the peri‐intervention period in patients undergoing general anaesthesia. Its potential to enhance patient wellbeing, reduce pain perception and decrease the need for pharmacological interventions, without associated adverse effects, could make it a valuable intervention [3, 4, 5].

However, the evidence of its efficacy in anaesthesia remains controversial. In 2021, a meta‐analysis including 50 randomised controlled trials (RCTs) suggested that hypnosis decreased mental distress and medication consumption in adults undergoing surgical procedures [6]. However, the generalisation of these results to anaesthesia practice remained limited since the authors included studies in which anaesthetists are usually not involved (i.e. hypnosis in dental practice), combined different timing of hypnosis implementation and because of irrelevant pooling of outcomes (i.e. regrouping of hypnotics, opioids, non‐opioid analgesics, anti‐emetics and vasopressors under the term ‘medication use’). Since 2021, new RCTs have been published, and an approach considering the perspective of the anaesthetist may help understand what can be reasonably expected from hypnosis in this context.

This systematic review aimed to describe the existing literature on the use of hypnosis for anaesthetists, quantify its impact and, when possible, identify knowledge gaps and outline directions for future research.

## Methods

Reporting follows PRISMA recommendations [7]. We changed the planned analyses by stratifying all results based on the timing of hypnosis administration to provide results more relevant to anaesthetists.

We included studies of any design in which hypnosis was used for any intervention requiring the presence of an anaesthetist, alone or in combination with any type of anaesthesia, on children and adults. We included studies exploring hypnosis for lumbar punctures, due to the similarity of the technique with spinal anaesthesia, and for transoesophageal echocardiography as this procedure is performed in the presence of anaesthetists in some centres. Any type of formal hypnosis was considered. We did not include studies on hypnosis for chronic pain or those related to obstetrics, dentistry, bone marrow puncture or burns. Studies involving informal hypnosis, hypnotic communication, muscle relaxation, suggestions without a formal hypnosis session or those using virtual reality or music were not included. Narrative reviews or surveys were also not considered.

A comprehensive search was conducted in PubMed, Embase and the Cochrane library in April 2024, and updated in February 2025 with the help of a librarian from the Faculty of Medicine of the University of Geneva, Switzerland. Bibliographies of systematic reviews and of retrieved studies were examined for additional references. A highly sensitive search strategy, without restriction on the publication period or language, was applied (online Supporting Information Appendix S1). Studies were selected using Covidence systematic review software (Veritas Health Innovation, Melbourne, VIC, Australia) [8], and the inclusion criteria were verified for all retrieved trials by the three authors separately (MJL, NE, STG). Two authors (MJL, STG) extracted data from included studies independently and entered them into a Microsoft Excel spreadsheet (Microsoft Corporation, Redmond, WA, USA) designed specifically for the purpose of this study. Any disagreements were resolved through discussion with the third author (NE). Data were verified by both MJL and STG to ensure accuracy and consistency. Data extracted included: name of the first author; publication year; country where the study was performed; study design; timing of hypnosis administration; type of anaesthesia; type of surgery or procedure; hypnosis practitioner; patient characteristics (sex and age); and all reported outcomes. We contacted authors to obtain missing data when required.

The primary qualitative outcome was the description of all retrieved studies and settings in which hypnosis was used by anaesthetists. The primary quantitative outcomes were the intra‐intervention consumption of hypnotics and opioids. Secondary quantitative outcomes included: pain scores and postoperative analgesic use; anxiety scores; satisfaction scores (for patients and the medical team); incidence of complications; incidence of postoperative nausea and vomiting (PONV); and duration of the procedures, post‐anaesthesia care unit (PACU) and hospital stay.

We stratified all meta‐analyses on three timings of hypnosis implementation: pre‐intervention; per‐intervention (hypnosis provided during the procedure/surgery with or without a pre‐intervention session); and post‐intervention (with or without a pre‐ or per‐ intervention session). To make the evidence as robust as possible, we report estimates based on RCTs only in the main text.

Differences in continuous outcomes were computed at the study level and combined into a mean difference, while binary outcomes were combined into weighted risk ratios. Standardised mean differences were computed when an outcome was reported using different scales and 95%CIs were used for all measures. Random‐effects models were used throughout due to the expected clinical heterogeneity across studies. For studies reporting medians (and IQR or range), we computed the means and standard deviations using the methods recommended in the Cochrane Handbook for Systematic Reviews of Interventions [9, 10]. When a single study reported on an effect that was largely different from the other studies, it was not included in the analyses. When an outcome was reported at different time‐points in the trials, we focused on the time‐point reported most frequently across trials. Then, when possible (i.e. enough trials available), we regrouped the time‐points into early (0–6 h), intermediate (6–24 h) or late (> 24 h) time‐points and assessed the result of the statistical test for heterogeneity across groups. When no statistical evidence of difference was found, the time‐points were pooled. We added the information provided in non‐randomised controlled studies, which included non‐randomised experimental trials and cohort studies with a control group, in separate additional analyses. Analyses were performed on STATA 18 (StataCorp, College Station, TX, USA) and cross checked in RevMAN (Cochrane Collaboration, London, UK).

The risk of bias was assessed according to each outcome using the revised Cochrane Risk of Bias tool for RCTs [11] and the ROBINS‐I tool [12] for non‐randomised controlled studies. For RCTs, if lack of blinding was unlikely to affect the interpretation of the outcome, we considered the risk of bias as low. For both RCTs and non‐randomised controlled studies, selective reporting bias was deemed high when there was a discrepancy between the outcome described in the registered protocol and those reported in the article, and as intermediate in case of doubt due to the absence of a registered protocol. Studies with critical risk of biases were excluded from analyses. The level of confidence in the point estimates was judged using GRADE recommendations [10].

## Results

**Figure 1:**
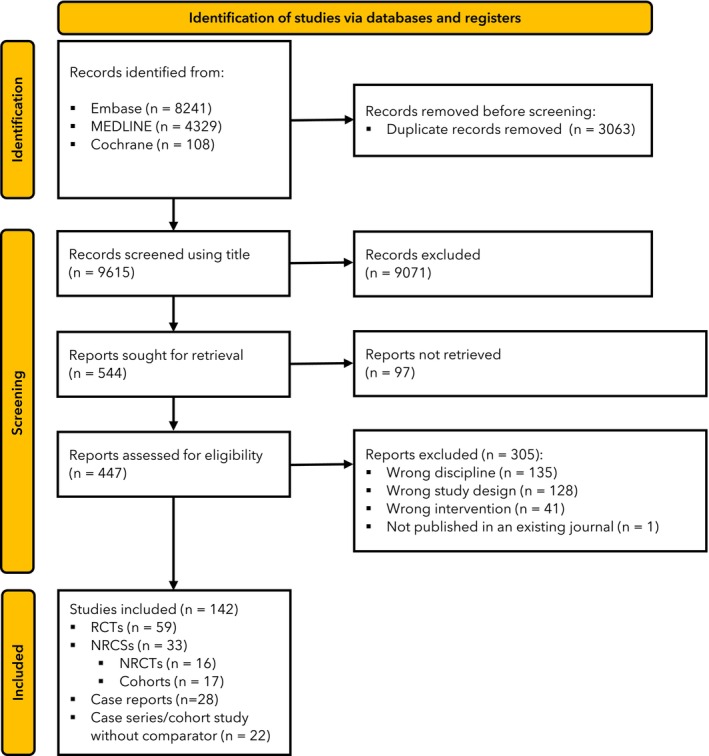
Study flowchart. RCTs, randomised controlled trials; NRCSs, non‐randomised controlled studies; NRCTs, non‐randomised controlled trials.

The initial search yielded 12,678 records. After removing double hits and screening titles for relevance, 12,134 records were omitted. The remaining 544 articles were assessed for eligibility by reading the full texts; 402 did not meet our inclusion criteria, leaving 142 studies included in the analyses. Six authors were contacted; two answered our queries. The 142 included studies (9238 patients; 8319 adults and 919 children) were regrouped by us into four design categories. The first category included 59 RCTs (4996 patients) [3, 4, 5, 13, 14, 15, 16, 17, 18, 19, 20, 21, 22, 23, 24, 25, 26, 27, 28, 29, 30, 31, 32, 33, 34, 35, 36, 37, 38, 39, 40, 41, 42, 43, 44, 45, 46, 47, 48, 49, 50, 51, 52, 53, 54, 55, 56, 57, 58, 59, 60, 61, 62, 63, 64, 65, 66, 67, 68]. The second category included 33 non‐randomised controlled studies (3191 patients), consisting of 16 non‐randomised controlled experimental trials and 17 cohort studies including a control group. The third category included 22 reports of more than one patient lacking a control group (1023 patients), therefore not allowing for the estimation of the effect of hypnosis (cohort studies without comparator, or case series). The last category included 28 case reports (Fig. 1 and online Supporting Information Appendices S2–S6).

Of the 50 RCTs included in the previous meta‐analysis [6], 19 did not fulfil our inclusion criteria and 28 new RCTs were included. Reasons for not included articles were that they focused on conversational hypnosis (n = 6) or virtual reality (n = 1); related to dentistry (n = 5) or burn management (n = 1); or were dissertations (n = 4); or abstracts (n = 2).

**Figure 2:**
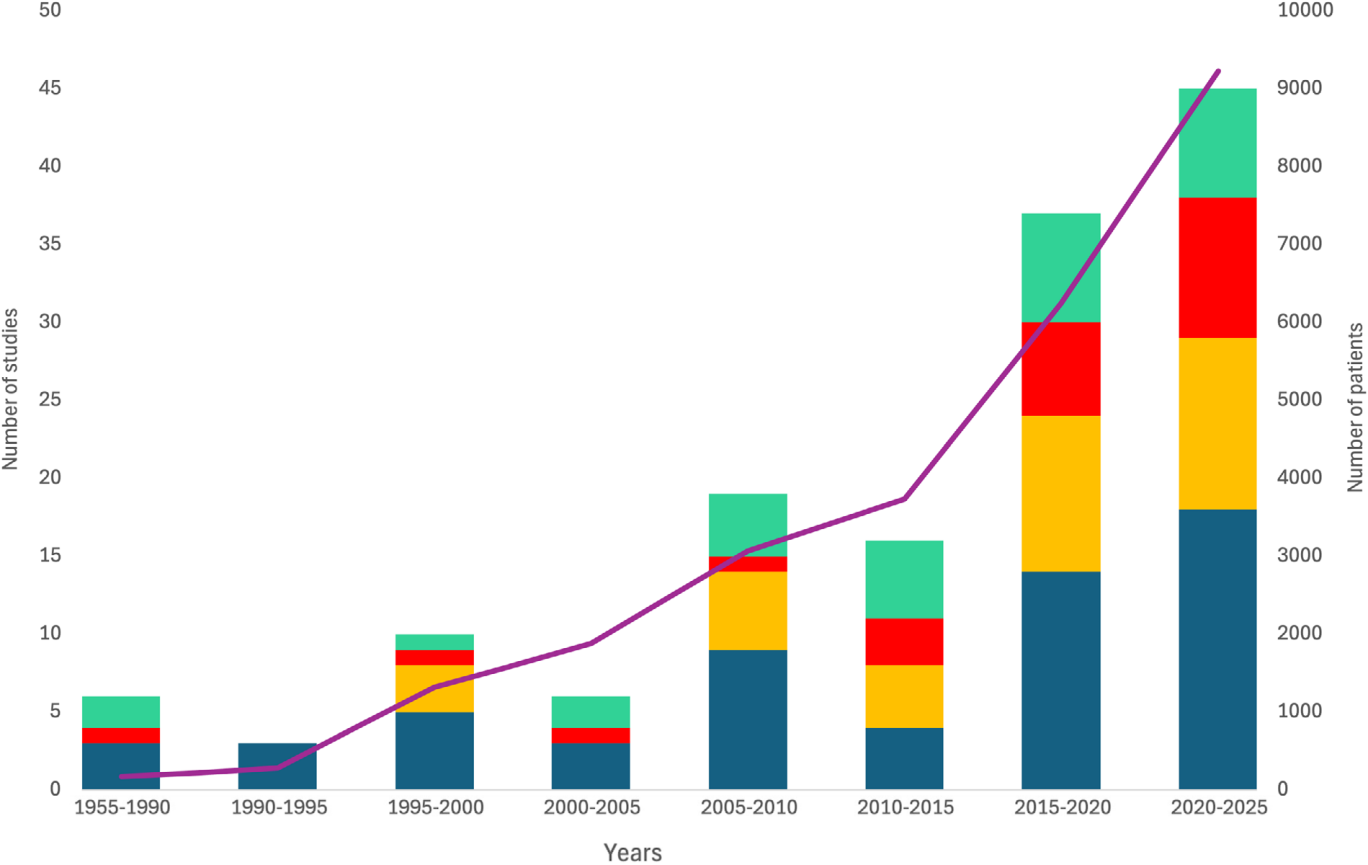
Temporal distribution of studies. Blue, randomised controlled trials; yellow, non‐randomised controlled studies; red, uncontrolled studies of more than one patient; green, case reports; violet line, cumulative number of patients.

Included studies were published from 1955 to 2025, of which 122 (86%) were published after 2000, and originated from 19 countries: France (n = 49); USA (n = 25); UK (n = 9); Italy (n = 8); and 17 studies from six different countries in Asia. Seven RCTs, six reports without a comparator, four case reports and three non‐randomised controlled studies focused on children aged from 3 y to 18 y (median 10 y). There was large heterogeneity in the surgeries and procedures examined, the type of anaesthesia and the timing of hypnosis administration (Table 1). The most studied fields were breast surgeries (n = 18); cardiac‐related procedures (n = 17); gynaecology (n = 14); and orthopaedic procedures (n = 10). Hypnosis was performed before the intervention only in 32 studies; pre‐ and/or per‐intervention in 96; or post‐intervention (with or without pre‐ and/or per‐intervention sessions) in 14. Hypnosis was provided by an anaesthetist (n = 42); nurse (n = 14); hypnotherapist (n = 13); psychotherapist (n = 11); anaesthetist or a nurse (n = 6); and by a non‐anaesthetist physician (n = 4). The practitioner was not specified in 25 studies. In 11 studies, hypnosis was auto‐induced (self‐hypnosis), audio recording was used in 15 and one trial applied a script. The hypnosis technique was described in detail in 108 articles, with 17 providing the complete script. Ericksonian hypnosis [69] was reported in 22 articles specifically. The use of hypnosis through suggestions was reported in 92 articles. Among these, 22 described the ‘safe place’ technique explicitly. Metaphors were used in 16 articles, including five describing the ‘magic glove’ technique [70]. The detailed characteristics of the included studies are provided in online Supporting Information Appendices S2–S5. Figure 2 illustrates the evolution of study designs over time.

**Table 1:** Hypnosis timing and type of anaesthesia categorised according to type of intervention.

	n	Hypnosis timing			Type of anaesthesia				
		PRE	PER	POST	LA/LR	GA	AS + LA	SC	None
Breast surgery	18	7	9	1	5	8	3	‐	1
Cardiac related	17	4	11	3	6	7	3	2	‐
Parathyroid and thyroid surgery	7	‐	6	1	2	3	2	‐	‐
Dermatology and plastic surgery	7	1	6	‐	‐	1	3	1	2
Otolaryngology	5	1	4	‐	1	2	1	‐	1
Gynaecology	14	4	10	‐	2	4	3	2	3
Neurosurgery	9	1	8	‐	2	3	4	‐	‐
Oncology	2	‐	‐	2	‐	2	‐	‐	‐
Ophthalmology	6	2	3	1	4	1	‐	‐	1
Orthopaedic	10	3	5	2	3	5	‐	1	1
Radiology/percutaneous	9	‐	8	1	4	1	1	1	2
Thoracic	3	1	‐	2	‐	3	‐	‐	‐
Urology	3	1	2	0	1	1	‐	‐	1
Visceral	5	1	3	1	‐	4	1	‐	‐
Procedures									
Endoscopy	6	1	5	0	2	‐	2	‐	2
Lumbar puncture	3	1	2	0	3	‐	‐	‐	‐
Mixed surgeries	6	2	4	‐	1	2	1	2	‐
Transoesophageal echocardiography	4	1	3	0	1	‐	2	1	‐
Venipuncture	8	1	7	0	3	‐	3	‐	2
Total	142	32	96	14	40	47	29	10	16

PRE, pre‐intervention; PER, per‐intervention; POST, post‐intervention; LA/LR, local or locoregional anaesthesia; GA, general anaesthesia; AS + LA, analgosedation and local anaesthesia; SC, standard care.

Twenty‐two RCTs examined the impact of hypnosis when implemented before the intervention (full results in Table 2). There was a low level of evidence of a reduction in post‐intervention pain and moderate evidence of a reduction in post‐intervention anxiety following pre‐intervention hypnosis. Clinical relevance was graded as moderate and high, respectively. One study examining anxiety [46] reported a result that was markedly different from the other RCTs and was omitted, but including the outlier RCT [46] did not impact the pooled result (standardised mean difference ‐1.01, 95%CI ‐1.52 to ‐0.51).

**Table 2:** Summary of results. Estimates are reported as risk ratios for binary outcomes, standardised mean difference for opioid and anxiety scores and mean difference for visual analogue scale (VAS) scores. Analyses are presented for all outcomes reported in ≥ three randomised controlled trials (RCTs).

	Pre‐intervention (22 RCTs)					Per‐intervention (28 RCTs)				
	RCTs	n	Estimate (95%CI)	I^2^	Grade of evidence	RCTs	n	Estimate (95%CI)	I^2^	Grade of evidence
**Primary outcomes**										
Any opioid during the intervention	3	266	‐0.64 (‐1.40–0.13)	82%	Moderate					
**Secondary outcomes**										
Pain during the intervention (VAS cm)						6	811	‐1.14 (‐1.86 to ‐0.41)*	91%	Moderate
Pain after the intervention (VAS cm)	7	419	‐0.88 (‐1.72 to ‐0.05)*	87%	Low	7	927	‐0.51 (‐1.46–0.43)	90%	Moderate
Need for class 1–2 analgesic (yes/no)	3	212	0.30 (0.06–1.60)	84%	Moderate					
Need for class 3 analgesic (yes/no)						4	340	0.77 (0.53–1.13)	81%	Moderate
Anxiety after the intervention (score)	8	515	‐0.76 (‐1.14 to ‐0.38)*	76%	Moderate	8	963	‐0.44 (‐0.75 to ‐0.13)*	79%	Low
Patient satisfaction (VAS cm)						4	465	0.27 (‐0.18–0.72)	62%	Moderate
PONV (yes/no)						3	185	0.43 (0.25–0.74)*	0%	High
Procedure duration (min)						7	1134	‐2.02 (‐3.81 to ‐0.23)*	51%	Moderate

n, number of patients in RCTs reporting on the outcome; PONV, postoperative nausea and vomiting.

* Statistically significant.

**Figure 3:**
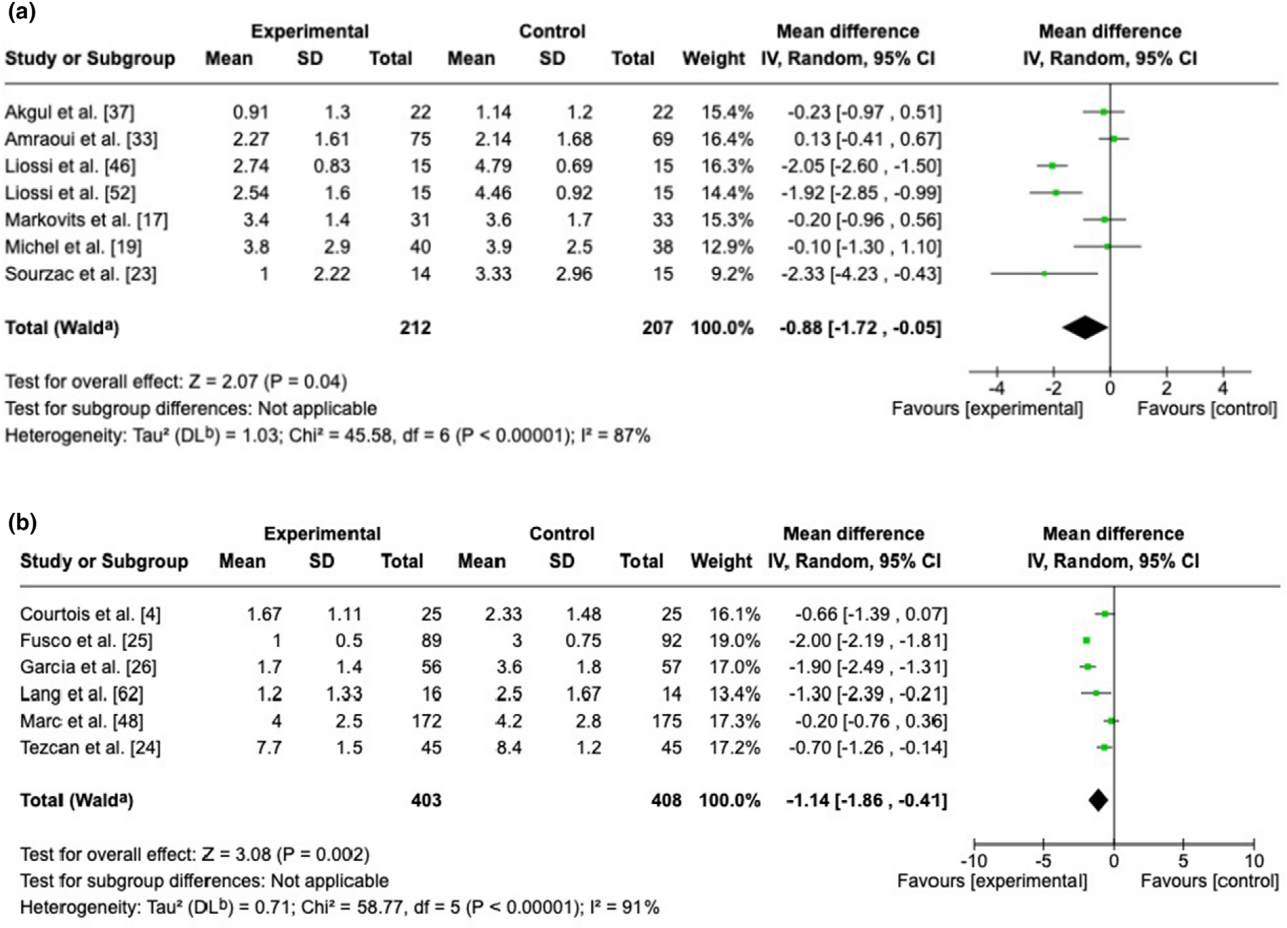
Meta‐analyses for visual analogue scale pain intensity: (a) after the intervention following a pre‐intervention hypnosis session; and (b) during the intervention following a per‐intervention hypnosis session. SD, standardised mean difference.

**Figure 4:**
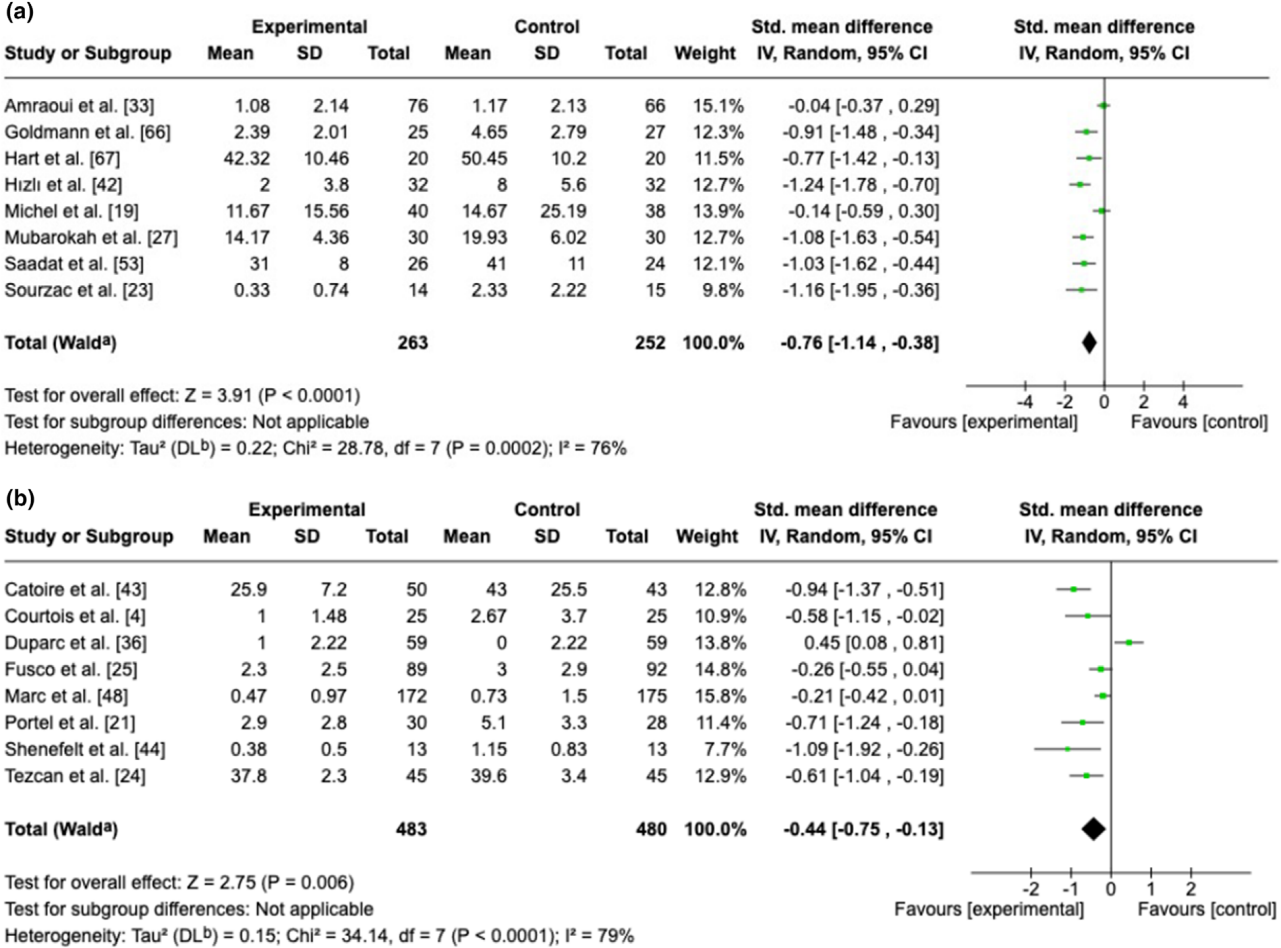
Meta‐analyses for anxiety after the intervention: (a) following pre‐intervention hypnosis session; and (b) following per‐intervention hypnosis session. SD, standardised mean difference.

Twenty‐eight RCTs examined the impact of hypnosis implemented during the intervention (Table 2). We found moderate evidence of a reduction in per‐intervention pain intensity (high clinical relevance), low evidence for a reduction in post‐intervention anxiety (high clinical relevance) and high‐quality evidence for a reduction in PONV (high clinical relevance). In addition, there was moderate evidence of a lack of impact of hypnosis on post‐intervention pain, on the need for class 3 analgesics and on patient satisfaction, and a clinically non‐relevant decrease in the duration of the procedure of about 2 min. Fewer than three RCTs reported on other outcomes. Figures 3 and 4 show forest plots of pain and anxiety scores.

Nine RCTs examined the impact of hypnosis implemented after the intervention, all of which combined the post‐intervention session with a pre‐intervention session [5, 13, 20, 22, 31, 59, 64], a per‐intervention session [55] or both [57]. Fewer than three RCTs reported on each outcome.

Six non‐randomised controlled studies examined the impact of hypnosis when implemented before the intervention. Integrating data from these non‐randomised controlled studies into the RCT‐based analyses gave consistent results regarding the ability of pre‐intervention hypnosis to decrease post‐intervention pain intensity and the trend towards a decreased need for class 1–2 analgesics, showing a similar point estimate and a result becoming statistically significant. It also suggested a potential to decrease propofol consumption by about 25 mg and to decrease PACU stay by 13 min (online Supporting Information Appendix S7).

Twenty‐four non‐randomised controlled studies examined the impact of hypnosis implemented during the intervention. Adding the evidence from these non‐randomised controlled studies provided additional evidence regarding the ability of per‐intervention hypnosis to decrease pain intensity during the intervention, to decrease anxiety post‐intervention and to decrease PONV. It also added data to the trend towards decreased pain intensity after the intervention, showing a similar point estimate and a result becoming statistically significant, and the lack of impact of per‐intervention hypnosis on patient satisfaction and on procedure duration. New evidence based on these non‐randomised controlled studies included a potential to decrease propofol consumption by about 112 mg; decreased need for additional per‐intervention analgosedation; decreased PACU stay by about 47 min; and suggested a lack of impact of per‐intervention hypnosis on medical team satisfaction. Three non‐randomised controlled studies examined the impact of hypnosis implemented after the intervention. Adding the evidence from these non‐randomised controlled studies to the results based on RCTs suggested a lack of impact of post‐intervention hypnosis on pain intensity (online Supporting Information Appendix S7. All detailed figures and risk of bias assessments for RCTs and non‐randomised controlled trials are available in Appendix S8).

## Discussion

This review summarises the available evidence regarding the use of hypnosis in the peri‐intervention setting, from the point of view of an anaesthetist. Our work illustrates several different methodological points. First, hypnosis has been studied for about 70 years, in many countries, in a wide variety of clinical settings, related to all types of anaesthesia. Despite 142 identified studies describing 9238 patients, of which 59 were RCTs, the overall quality of the retrieved studies was weak and the level of evidence of the impact of hypnosis in this setting ranges from absent to moderate. Second, most of the retrieved studies concerned gynaecological procedures and breast surgery, followed by cardiac‐related procedures. Third, hypnosis was mainly implemented before and during the interventions; hypnosis applied after the intervention was examined rarely. Fourth, despite wide heterogeneity among the studies, the hypnosis technique itself remained consistent, mainly employing suggestions and metaphors. Fifth, hypnosis was applied by different practitioners with a large variability of outcomes reported thus illustrating the lack of clear research agenda and of clear expectations regarding what hypnosis is expected to improve. Finally, well performed RCTs with adequate blinding of investigators exist, are feasible and should be aimed for.

Our work also clarifies some clinical points. Pre‐intervention hypnosis may reduce anxiety and pain after the intervention, which is clinically relevant. The data concerning per‐intervention hypnosis suggest that it could decrease pain intensity during the procedure by about 1 cm on the 1–10 cm visual analogue scale without impacting on pain intensity after the procedure; reduce anxiety after the procedure; and reduce the risk of PONV by half, with grades of evidence ranging from moderate to high. All these endpoints are important to patients. Adding data from non‐randomised controlled studies to RCTs provides consistent estimates of these benefits. Evidence regarding the impact of post‐intervention hypnosis sessions is lacking.

These findings need to be discussed considering what is already known. The minimal clinically relevant difference in visual analogue pain score was shown to be as high as 2 points on the 11 points numerical pain scale for chronic pain [71, 72], and to differ widely across clinical settings and baseline pain [73]. In the postoperative period, where good analgesia can be achieved with pain scores < 4 cm, a difference of 1 cm has been suggested to be clinically important [74]. In this context, the reduction achieved through hypnosis appears particularly relevant. Interestingly, patients tend to experience less pain when hypnosis is used alongside a procedure, but without any difference in pain score after the procedure. This may reflect variations in pain perception. Brain imaging studies suggest that hypnosis can modulate subjective pain perception by influencing two distinct brain regions: the somatosensory cortex, which processes pain intensity; and the anterior cingulate cortex, which handles pain unpleasantness [75]. Therefore, hypnotic suggestions that focus on comfort and reducing pain unpleasantness may not alter the reported intensity of pain but can improve the overall perception of pain. Unfortunately, there is no validated measurement of the perception of pain, and our result contrasts with previous studies [76, 77].

Postoperative anxiety is a relevant outcome for both patients and anaesthetists, and its reduction through hypnosis could also improve the risk of chronic pain development [78]. The ability of hypnosis to reduce post‐intervention anxiety makes it appealing. Another key priority for anaesthetists and patients is the management of PONV. It has been suggested that hypnosis may have a place in treating and preventing nausea and vomiting during pregnancy [79], as well as during chemotherapy [80]. Our study suggests that it may also have an impact in the peri‐intervention setting, where per‐intervention hypnosis was shown to decrease the risk of PONV by half. This large effect, which is highly clinically relevant, is comparable with that observed with intravenous dexamethasone [81]. However, it is based on only three RCTs and should be confirmed in future trials.

The slight reduction in propofol dosage observed in patients who undergo pre‐intervention hypnosis sessions is likely of limited relevance to patients themselves, but it may be of interest to anaesthetists. This reduction could be clinically meaningful in specific contexts, such as in older patients or those with cardiac insufficiency or haemodynamic instability. The reduction of analgosedation needs provides new and clearer perspectives for anaesthetists than the findings from the meta‐analysis by Holler et al. [6], where the authors reported less “*medication consumption*” but combined all sorts of medications. However, these reductions in analgosedation needs should be interpreted cautiously, as our results are based on only two RCTs and one non‐randomised controlled study, and the lack of blinding of the anaesthetists in these studies could have influenced the administration of hypno‐analgesics.

The absence of impact of per‐intervention hypnosis on patient satisfaction was a surprise. This may be explained by a ceiling effect with patients being all highly satisfied with their care, whether hypnosis is used or not, making it difficult to detect meaningful differences. This is well‐recognised in the field of pain, summarised as “*no pain, no gain*” [82]. Nevertheless, as hypnosis is added increasingly to patient care [83], future rigorous studies are needed to clarify its impact on patient satisfaction.

We found a wide range of practitioners delivering hypnosis in the peri‐intervention context. This highlights the potential for collaborative practices. Trained nurses and psychologists can deliver peri‐intervention hypnosis, working in close collaboration with the surgical and anaesthesia teams [84]. The role of patients as an active proponent of their intervention through self‐hypnosis is also interesting as it may result in competencies that could be used throughout their lives. As anaesthetists remain the main hypnosis providers in these studies, they could be trained in this field. A recent publication exposed a pragmatic approach to help anaesthetists integrate hypnotic techniques into their daily practice [85].

Our study has limitations. First, despite an exhaustive search, relevant studies may have been omitted. Our search identified publications from the 1950s that we were unable to access. From their (vague) titles, we do not know whether they would have been relevant to our review. Also, we did not search for unpublished studies. Second, the quality of the evidence produced is limited by the low quality of the studies identified. It is a shame that 70 years of publications on hypnosis in the peri‐intervention setting has led to such poor evidence of its impact. This is due to poor designs and inadequate reporting. Third, we had to deal with huge heterogeneity across included studies. We chose an approach to be able to give meaningful answers to anaesthetists, based on the timing of hypnosis administration. Significant heterogeneity remained that was not all explained by differences in anaesthetic techniques and study designs. We did not check for the impact of the hypnosis practitioners or types of hypnosis administered. Meta‐regression was not possible due to the small number of studies reporting on a given endpoint. Fourth, we converted data that were reported as median (IQR/range) to mean (SD). This method is appropriate when the data are approximately normally distributed and the sample size is sufficiently large, but remains an area of approximation. Fifth, some relevant results were reported inconsistently or could not be obtained even after contacting the authors. In one randomised trial, even the number of patients analysed per group was not reported clearly. This questions the quality of the peer‐review of these studies. Finally, the grading of evidence of most of our results was only moderate, and although we analysed extensive data from approximately 5000 patients across 59 RCTs, we remained unable to settle on many relevant anaesthetic outcomes.

Our work aimed to help identify knowledge gaps that should guide future research. The impact of hypnosis sessions conducted in the post‐intervention phase remains a largely overlooked topic. The evidence regarding the impact of pre‐intervention hypnosis sessions is also weak and deserves further well‐designed large RCTs. Evaluation of the cost‐effectiveness of hypnosis sessions is required and necessary to support the adoption of this technique, although it is difficult to address. Finally, the main future challenge will be selecting outcomes that truly matter to patients in clinical practice. These should be explored from a patient and public involvement perspective in future RCTs.

The evidence regarding the added value of hypnosis for anaesthetists in the peri‐intervention setting remains weak. This systematic review suggests that it may have an impact on anxiety following an intervention, on pain during and after the intervention, and on PONV incidence when hypnosis is administered during the intervention. Its influence on most other clinical outcomes remains unknown.

## Supporting information


**Appendix S1.** Search strategy.
**Appendix S2.** Randomised controlled trials.
**Appendix S3.** Non‐randomised controlled studies.
**Appendix S4.** Uncontrolled studies of more than one patient.
**Appendix S5.** Case reports.
**Appendix S6.** Bibliography of included studies.
**Appendix S7.** Analyses including non‐randomised controlled studies data.
**Appendix S8.** Figures of quantitative analyses.
