# Supplementary material for: Hypnosis for anaesthetists: a systematic review and meta‐analyses
Source: Anaesthesia. 2025 Sep 24;81(1):103–15. doi: 10.1111/anae.70013 (PMC12747654; doi:10.1111/anae.70013)
Supplement: Supplementary file 1 — Appendix S1. Search strategy. Appendix S2. Randomised controlled trials. Appendix S3. Non‐randomised controlled studies. Appendix S4. Uncontrolled studies of more than one patient. Appendix S5. Case reports. Appendix S6. Bibliography of included studies. Appendix S7. Analyses including non‐randomised controlled studies data. Appendix S8. Figures of quantitative analyses. [file ANAE-81-103-s001.pdf]

Appendix S1: Search equations

Embase:

('hypnosis'/exp OR 'hypnosis':ti,ab,kw OR 'hypnotherap*':ti,ab,kw OR 'hypnoti*':ti,ab,kw OR 'self-hypnosis':ti,ab,kw OR 'self-hypnotic':ti,ab,kw OR 'autohypnosis':ti,ab,kw OR 'hypnoa*nesthesia':ti,ab,kw OR 'hypnosedation*':ti,ab,kw) AND ('anesthesist'/exp OR 'anesthesiology'/exp OR 'anaesthes*':ti,ab,kw OR 'surgery'/exp OR 'surgery' OR 'perioperative':ti,ab,kw OR 'postoperative':ti,ab,kw OR 'preoperative':ti,ab,kw OR 'operati*':ti,ab,kw OR 'intraoperative':ti,ab,kw OR 'surgery':ti,ab,kw OR 'surgical':ti,ab,kw)

Pubmed:

("surgical procedures, operative"[MeSH Terms] OR "Surgery"[MeSH Subheading] OR "Perioperative"[Title/Abstract] OR "Postoperative"[Title/Abstract] OR "Preoperative"[Title/Abstract] OR "operati*"[Title/Abstract] OR "Intraoperative"[Title/Abstract] OR "Surgery"[Title/Abstract] OR "surgical"[Title/Abstract] OR ("Anesthetists"[MeSH Terms] OR "Anesthesiology"[MeSH Terms] OR "Anaesthesia"[Title/Abstract] OR "Anesthesia"[Title/Abstract] OR "Anaesthesias"[Title/Abstract] OR "Anesthesiology"[Title/Abstract] OR "Anaesthesiology"[Title/Abstract] OR "Anaesthetist"[Title/Abstract] OR "Anesthetist"[Title/Abstract] OR "anesthesia assistant*"[Title/Abstract] OR "anaesthesia assistant*"[Title/Abstract])) AND ("Hypnosis"[MeSH Terms] OR "hypnosis, anesthetic"[MeSH Terms] OR "Hypnosis"[Title/Abstract] OR "hypnotherap*"[Title/Abstract] OR "hypnoti*"[Title/Abstract] OR "self-hypnosis"[Title/Abstract] OR "self-hypnotic"[Title/Abstract] OR "Autohypnosis"[Title/Abstract] OR "hypnoa*nesthesia"[Title/Abstract] OR "hypnosedation*"[Title/Abstract])

Appendix S2: Randomized controlled trials

AUTHOR	YEAR	COUNTRY	PROCEDURE	HYPNOSIS				CONTROL				PRACTITIONER	TIMING
				N	Age (years)	Sex M/F	Anaesthesia	N	Age (years)	Sex M/F	Anaesthesia		
Azam	2024	Canada	Oncology	29	56 (12)	14/15	GA	28	56 (10)	12/16	GA	Psychologist	PRE, POST
Catalano	2024	Switzerland	Neurosurgery	10	63.8 [43-73]	5/5	LA	9	62 [42-72]	8/1	LA	Anaesthesiologist	PRE, PER
Polomeni	2024	France	Mixed surgeries	47	[7-14] °	25/22	None	49	[7-14] °	23/26	LA	Hypnotherapist	PER
Rosenbloom	2024	Canada	Oncologic	45	58.26 (12)	18/21	GA	47	57 (10)	21/24	GA	Psychologist	PRE, POST
Sola	2023	France	Dermatology	30	9.5 [7-16]	17/13	GA	30	10 [7-16]	11/19	GA	Anaesthesiologist/ Nurse	PRE, PER
Courtois	2022	France	Lumbar puncture	25	77 (5)	10/15	LA	25	77 (5)	38/12	LA	Psychologist	PER
Markovits	2022	USA	Orthopaedic	31	70.6 [48 -87	11/20	GA	33	65 [42-92]	16/17	GA	nr	PRE
Meyer	2022	USA	Gynaecology	71	54 (40.6)	0/71	SC	67	61 (47)	0/67	SC	Recording	PRE
Michel	2022	France	Thoracic	40	42 (26)	27/13	GA	38	42.5 (23)	19/19	GA	Anaesthesiologist	PRE
Moreno	2022	Mexico	Breast surgery	20	nr [30-80]	0/20	GA	20	nr [30-80]	0/20	GA	Recording	PRE, POST
Portel	2022	France	Endoscopy	30	65 (13)	17/13	LA	28	62 (12)	24/6	LA	Nurse	PER
Rousseaux	2022	Belgium	Cardiac related	25	68 (12)	18/7	GA	25	63 (11)	18/7	GA	Recording	PRE, POST
Viegas	2022	Canada	Cardiac related	39	13 [11-15]	18/21	GA	39	13 [11-15]	19/20	GA	Script	PRE, PER
Sourzac	2021	France	Gynaecology	14	63 (nr)°	0/14	GA	15	63 (nr)°	0/15	GA	Nurse	PRE
Tezcan	2021	Turkey	Urology	45	64.5 (7.5)	45/0	LA	45	64 (7)	45/0	LA	nr	PRE, PER
Fusco	2020	France	Venipuncture	89	56 [28-89]	41/48	None	92	54 [18-86]	44/48	None	Anaesthesiologist/ Nurse	PER
Garcia*	2020	France	Cardiac related	56	70 (12)	43/13	LA	57	70 (12)	46/11	LA	Nurse	PER
Innayatun	2020	Indonesia	Gynaecology	30	nr	0/30	LR	30	nr	0/30	LR	nr	PRE
Nowak	2020	Germany	Mixed surgeries	191	52 [43-62]	76/115	GA	194	54 [46-62]	84/110	GA	Recording	PER
Efsun	2019	Turkey	ENT	11	26 (5.2)	6/5	GA	11	26 (10)	6/5	GA	Anaesthesiologist	PRE
Hoslin	2019	France	Venipuncture	77	61 (13)	28/49	LA	71	57 (14)	27/44	LA	nr	PRE, PER
Lee	2019	Malaysia	Orthopaedic	8	66 (9.3)	01.Jul	GA	8	68 (10.4)	0/8	GA	Recording	PRE, POST
Sánchez	2019	Mexico	Breast surgery	58	48 (10)	0/58	LA	57	48 (12)	0/57	LA	Recording	PRE
Amraoui	2018	France	Breast surgery	77	53 [20-84]	0/77	GA	71	57 [33-79]	0/71	GA	Anaesthesiologist	PRE
Bataille	2018	France	Gynaecology	31	41 [37-46]	0/31	GA	34	42 [38-45]	0/34	GA	Anaesthesiologist/ Nurse	PER
Chen	2018	China	Ophthalmology	55	64 (5.7)	25/30	LA	56	65 (5)	27/29	LA	nr	PRE
Duparc-Alegria	2018	France	Orthopaedic	59	15 [10-18]	19/40	GA +/- LR	59	15 [13-16]	15/44	GA +/- LR	Nurse	PER
Akgul	2016	Turkey	Cardiac related	22	54 (10)	17/5	GA	22	55 (9)	18/4	GA	Anaesthesiologist	PRE
Behnaz	2016	Iran	Ophthalmology	45	nr [50 to 75]	47/43°	LA	45	nr [50 to 75]'	47/43°	LA + AS	Psychologist	PER
Corman	2016	France	TOE	47	60 (15)	31/16	LA	51	55 (14)	29/22	LA	Psychologist	PRE
Dogan	2016	Turkey	TOE	27	39 (9.2)	11/16	LA	49	35 (13)	26/23	LA + sedation	Anaesthesiologist	PRE, PER
Joudi	2016	Iran	Visceral	60	43 (9.4)	nr	GA	60	43 (14)	nr	GA	Recording	PRE
Hizli	2015	Turkey	Urology	32	63.5 (6)	32/0	None	32	62 (7)	32/0	None	Hypnotherapist	PRE
Catoire	2013	France	Venipuncture	50	30 (3.6)	0/50	None	43	32 (3.5)	0/43	Sedation	nr	PER
Shenefelt	2013	USA	Dermatology	13	59 [23-75]	7/6	nr	13	66 [48-76]	7/5	nr	Nurse	PER
Reinhard	2012	Germany	Gynaecology	78	31 (4.4)	0/78	None	122	32.5 (5)	0/122	None	Obstetrician	PER
Liossi	2009	UK	Venipuncture	15	8.4 (2)	6/9	LA	15	8.4 (2)	8/7	LA	Hypnotherapist	PRE
Lang	2008	USA	Percutaneous	66	48 [33-75]	21/45	LA	70	50.5 [29-79]	31/39	LA	Self-hypnosis	PER
Marc	2008	Canada	Gynaecology	172	26 (6)	0/172	Sedation	175	24 (5)	0/175	Sedation	nr	PRE, PER
Schnur	2008	USA	Breast surgery	49	46 (2)	0/49	GA	41	45 (2)	0/41	GA	Psychologist	PRE
Marc	2007	Canada	Gynaecology	14	27 (7.2)	0/14	None	15	26 (5)	0/15	Nitrous oxide	nr	PRE, PER
Montgomery	2007	USA	Breast surgery	105	49 (13)	0/105	LA + AS	95	48 (13)	0/95	LA + AS	Psychologist	PRE
Liossi	2006	UK	Lumbar puncture	15	nr [6-16]	nr	LA	15	nr [6-16]	nr	LA	Psychologist	PRE
Saadat	2006	USA	Mixed surgeries	26	42 (10)	18/8	nr	24	43 (10)	19.May	nr	nr	PRE
Calipel	2005	France	Mixed surgeries	23	4.5 [2-10]	19/4	None	27	5 [2-11]	21/6	Sedation	Anaesthesiologist	PRE, PER
Cowan	2001	USA	Visceral	10	37 (nr)	2/8	GA	17	36 (nr)	0/17	GA	Recording	PER, POST
Defechereux	2000	Belgium	Parathyroid/thyroid surgery	20	46 (12)	1/19	AS	20	45 (15)	4/16	GA	Anaesthesiologist	PER
Lang	2000	USA	Percutaneous	66	48 [33-75]	21/45	LA	70	50.5 [29-79]	31/39	LA	Self-hypnosis	PRE, PER, POST
Conlong	1999	UK	Endoscopy	33	58 [19-92]	nr	LA	46	58 [19-92]	nr	LA	Gastroenterologist	PER
Ashton	1997	USA	Cardiac related	20	64 (3)	17/3	GA	12	62 (3)	11/1	GA	Self-hypnosis	PRE, POST
Enqvist	1997	Sweden	Breast surgery	23	39 [22-63]	0/23	GA	25	41.5 [24-69]	0/25	GA	Recording	PRE
Faymonville	1997	Belgium	Plastic	31	36 (14)	3/29	LA + AS	25	34 (10)	4/21	LA + AS	Anaesthesiologist	PER
Lang	1996	USA	Radiology	16	69 [54-83]	16/0	Sedation	14	64 [44-78]	14/0	Sedation	nr	PRE, PER
Williams	1994	UK	Gynaecology	22	nr	0/22	GA	29	nr	0/29	GA	Recording	PER
Greenleaf°°	1992	USA	Cardiac related	nr	nr	nr	GA	nr	nr	nr	GA	Psychologist	PRE, POST
Weinstein	1991	USA	Cardiac related	16	60 (8)	nr	LA	16	59 (11)	nr	LA	Hypnotherapist	PRE, PER
Goldmann	1988	UK	Gynaecology	25	nr	0/25	LA	27	nr	0/27	LA	nr	PRE
Hart	1980	USA	Cardiac related	20	nr	16/4	nr	20	nr	17/3	nr	Recording	PRE
Field §	1974	USA	Orthopaedic	30	nr	nr	nr	30	nr	nr	nr	Recording	PRE

Age is reported as mean (SD) or [range]; M: Male; F: Female; nr: not reported. AS: analgosedation; SC: standard care; LA: local anesthesia; GA: general anesthesia; PRE: preoperative; PER: peroperative; POST: postoperative; ENT: Ear Nose Throat; TEE: Trans-esophageal echography; *Garcia 2020: white noise and suggestions were added to LA in the control group; °: the value is reported for the entire sample; °°Greenleaf 1992: 32 patients were randomized in 3 groups. The number of included patients per groups are not reported. The mean (sd) age of the sample was 59 (9) and the male/female ratio was 26/6. §Field 1974: the male/female ratio was only reported for the entire sample (58/2).

Appendix S3: Non-randomized controlled studies (NRCS)

AUTHOR	COUNTRY	PROCEDURE	DESIGN	HYPNOSIS				CONTROL				PRACTITIONER	TIMING
				N	Age (years)	Sex M/F	Anaesthesia	N	Age (years)	Sex M/F	Anaesthesia		
Berlière	Belgium	Breast surgery	non randomized trial	95	60 [34-86]	0/95	LA + AS	93	62 [28-83]	0/93	GA	Anaesthesiologist	PER
Cossu	Switzerland	Neurosurgery	controlled cohort	14	43 (9)	7/12	LA + AS	8	50 (11)	4/4	GA	Hypnotherapist	PER
Derycke	France	Cardiac related	controlled cohort	28	70 (10.5)	28/0	AS	43	75 (8)	38/5	GA	Anaesthesiologist	PER
Bankole	France	Neurosurgery	controlled cohort	26	41.5 (12)	11/15	LA	35	41.5 (12)	19/16	Sedation	Anaesthesiologist	PRE, PER
Loseto°	France	Percutaneous	non randomized trial	21	59 (nr)°	nr	LA	10	59 (nr)°	nr	LA	Nurse	PER
Badidi	France	Cervical endocrin	controlled cohort	50	50 (nr)	15/35	LA + AS	50	50 (nr)	15/35	GA	Anaesthesiologist/ Nurse	PRE, PER
Juana María	Spain	Dermatological	non randomized trial	33	8 (2)	nr	Sedation	32	8 (3)	nr	Sedation	Anaesthesiologist	PRE
Scaglione	Italy	Cardiac related	non randomized trial	11	57 (12)	9/2	LA+ AS	19	53 (14)	13/6	LA+ AS	Nurse	PER
Pesce	Italy	Neurosurgery	controlled cohort	6	52 [22-72]	nr	LR	9	54 [34-71]	nr	LR + Sedation	nr	PER
Takahashi	France	Cardiac related	controlled cohort	36	85 (7)	15/21	LA + Sedation	107	84 (7)	51/56	LA + Sedation	nr	PER
Touzé	France	Cervical endocrin	controlled cohort	19	61 (nr)	10/9	LA + Analgesic	17	64 (nr)	6/14	LA + AS	Anaesthesiologist/ Nurse	PER
Chapet	France	Urologic	controlled cohort	68	66 (6)	68/0	LA	79	66 (6)	79/0	GA or AS	Anaesthesiologist	PER
Lacroix	France	Breast surgery	non randomized trial	21	60 [36-79]	0/21	LR + AS	21	58 [39-75]	0/21	LR+ GA	Anaesthesiologist	PER
Berlière	Belgium	Breast surgery	non randomized trial	150	59.5 (nr)	0/150	AS	150	58 (nr)	0/150	GA	Anaesthesiologist	PER
Boselli	France	Orthopaedic	non randomized trial	50	48 [33-53]	21/29	LR	50	34 [30-50]	20/30	SC	Anaesthesiologist	PRE
Manworren	USA	Thoracic	controlled cohort	24	15 (2)	22/2	GA + IV LA or Th. EP	29	15 (2)	25/4	GA + IV LA or	Self-hypnosis	PRE, POST
Zech	Germany	Neurosurgery	controlled cohort	64	62 (7.6)	46/18	LA+ AS	22	63 (7.6)	14/8	GA	Anaesthesiologist	PER
Agard	France	Ophthalmologic	non randomized trial	102	74.5 (7)	nr	LA	69	73 (7)	nr	LA	Nurse	PRE, PER
Romain	France	Visceral	controlled cohort	35	60 [27-81]	31/4	LR	55	60 [21-85]	51/4	LR or LR + GA	Nurse	PER
Zemmoura	France	Neurosurgery	controlled cohort	37	41 [18-67]°	19/18	LA + AS	33	41 [18-67]°	nr	GA	Anaesthesiologist	PRE, PER
Eren	Turkey	TEE	non randomized trial	45	39 (13.7)	20/25	None	41	36 (14)	21/20	Sedation	Anaesthesiologist	PRE, PER
Coveney	UK	Breast surgery	controlled cohort	39	nr	0/39	GA	38	nr	0/38	GA	nr	PRE
Gauchotte	France	Gynaecologic	controlled cohort	30	nr	0/30	None	40	nr	0/40	None	Nurse	PER
Lew	USA	Breast surgery	non randomized trial	20	nr [30-79]	0/20	GA	18	nr [30-79]	0/18	GA	Hypnotherapist	PRE
Musellec	France	Gynaecologic	controlled cohort	12	39 (3)	0/12	AS	12	41 (3.5)	0/12	GA	Anaesthesiologist	PER
Novoa	USA	Cardiac related	non randomized trial	50	67 (10)	36/14	GA	50	66 (9.6)	36/14	GA	Surgeon	PRE
Bouté	France	Breast surgery	non randomized trial	14	nr	0/14	LA	14	nr	0/14	LA	Anaesthesiologist	PER
Levitas	Israel	Gynaecologic	controlled cohort	89	32 (4)	0/89	SC	96	32 (5)	0/96	SC	nr	PRE, PER
Elkins	USA	Endoscopy	controlled cohort	6	58 (6.2)	5/1	AS	10	56 (7.2)	3/7	AS	Recording	PER
Lobe	USA	Thoracic	non randomized trial	5	nr [12-18]°	nr	GA + PCA	5	nr [12-18]°	nr	GA + Th.EP	Self-hypnosis	PRE, POST
Defechereux	Belgium	Cervical endocrin	non randomized trial	218	48.5 (nr)	49/169	AS	119	59.5 (nr)	27/92	GA	Anaesthesiologist	PRE,PER,POST
Enqvist	Sweden	ENT	non randomized trial	45	24 (8.3)	12/33	GA	45	23 (7)	24/21	GA	Recording	PRE, PER
Faymonville	Belgium	Plastic surgery	non randomized trial	172	36 (18)	50/122	AS	137	39 (15)	35/102	AS	Anaesthesiologist	PER

Age is reported as mean (SD) or [range]; M: Male; F: Female; nr: not reported. AS: analgosedation; SC: standard care; LA: local anesthesia; LR: locoregional; GA: general anesthesia; Th. EP: Thoracic epidrual; PCA: Patient controlled analgesia; PRE: preoperative; PER: peroperative; POST: postoperative; °: the age was reported for the entire sample.

Appendix S4: Uncontrolled studies of more than one patient

AUTHOR	YEAR	COUNTRY	PROCEDURE	HYPNOSIS				PRACTITIONER	TIMING
				N	Age (years)	Sex M/F	Anaesthesia		
Bobin	2023	France	Endoscopy	23	51 (11.7)	16/7	None	nr	PRE, PER
Fontanges	2023	France	Cardiac related	16	10.5 [4-16]	nr	LA	nr	PER
Chandrasegaran	2022	Malaysia	Ophtalmology	2	34-55	1/1	LA	Anaesthesiologist	PER
Wood	2022	France	Neurosurgery	74	54 (12.5)	39/35	LA	Hypnotherapist	PRE, PER
Lind	2021	Norway	Breast surgery	5	nr [40-70]	0/5	GA	Hypnotherapist	PRE
Lopes	2021	France	Neurosurgery	46	54 [23-85]	24/22	GA	Nurse	PRE
Tran	2021	France	Endoscopy	140	12 [9-14]	70/70	Sedation	Nurse	PRE
Jaouen	2020	France	ENT	51	63 [22-87]	14/37	LA + Analgesic	Anaesthesiologist/ Nurse	PRE, PER
Kissel	2020	France	Percutaneous	20	59 [36-80]	0/20	LA + Sedation	Radiation therapist	PER
Amedro	2019	France	TOE	16	nr [11-18]	9/7	None or GA or AS	Hypnotherapist	PER
Fathi	2019	Iran	Orthopaedic	2	33.5 (nr)	1/1	LA	Anaesthesiologist	PER
Barbero	2018	Italy	Cardiac related	5	nr	2/3	LA	Nurse	PER
Sterkers	2018	France	Venipuncture	30	54 [35-77]	0/30	LA+AS	Recording	PRE, PER
Claude	2016	France	Radiotherapy	132	3 [0.4-5]	74/58	GA	nr	PRE, PER
Bouzinac	2012	France	Breast surgery	3	nr	0/3	LA+ Analgesic	Anaesthesiologist	PER
Galy	2012	France	Cardiac related	150	70 (nr)	107/43	LR	Anaesthesiologist	PER
Dominguez	2010	Spain	Endoscopy	26	nr [20-67]	14/12	None	nr	PRE, PER
Hermes	2005	Germany	ENT	209	nr [13-87]	nr	LA	Recording	PRE, PER
Séfiani	2004	France	Visceral	50	52 (64)	3/47	LA+ AS	Anaesthesiologist	PER
Bertoni	1999	Italy	Radiotherapy	3	nr [4-5]	2/1	None	Psychotherapist	PRE, PER
Adams	1992	Canada	Percutaneous	2	19-44	0/2	None or LA	Psychotherapist	PRE, PER
Alvin	1976	USA	Orthopaedic	18	nr [12-22]	3/15	GA	Anaesthesiologist	PER

Age is reported as mean (SD) or [range]; M: Male; F: Female; nr: not reported. AS: analgosedation; LA: local anaesthesia; LR: locoregional; GA: general anaesthesia; TEE: Trans-oesophageal echography; ENT: Ear Nose Throat; PRE: pre-intervention; PER: per-intervention; POST: post-intervention.

Appendix S5: Case reports

AUTHOR	YEAR	COUNTRY	PROCEDURE	HYPNOSIS			PRACTITIONER	TIMING
				Age (years)	Sex M/F	Anaesthesia		
Bapteste	2023	France	Neurosurgery	12	M	Sedation	nr	PRE, PER
Oung	2023	Switzerland	Parathyroid/thyroid surgery	60	F	Analgesic	Anaesthesiologist	PRE, PER
Chandrasegaran	2022	Malaysia	Orthopaedic	44	M	LR	Anaesthesiologist	PER
Pilia	2022	Italy	Visceral	56	M	GA	Anaesthesiologist	PRE, PER
Fathi	2021	Iran	Ophthalmology	54	F	None	Anaesthesiologist	PER
Vanreusel	2021	Belgium	Cardiac related	66	F	LA	nr	PER
Makovac	2020	Switzerland	Parathyroid/thyroid surgery	33	M	LA+ Analgesic	Anaesthesiologist	PER
Ibañez del Prado	2019	Spain	Venipuncture	52	M	Sedation	Hypnotherapist	PRE, PER
Al-Nasser	2018	France	Parathyroid/thyroid surgery	76	M	LA	Anaesthesiologist	PER
Cholet	2017	France	Cardiac related	67	M	LA+ Analgesic	Anaesthesiologist	PER
Fathi	2017	Iran	Gynaecology	51	F	None	Anaesthesiologist	PER
Fuzier	2017	France	Breast surgery	58	F	LA+ Analgesic	Nurse	PRE, PER
Fiddaman	2016	UK	Breast surgery	85	F	LA	Hypnotherapist	PRE, PER
Bienvenu	2015	France	Orthopaedic	20	F	GA	Hypnotherapist	PRE. PER. POST
Antonelli	2014	Italy	mixed surgeries	35	M	GA	Anaesthesiologist	PRE, PER
Facco	2013	Italy	Dermatology	42	F	None	nr	PRE, PER
Wong	2011	Australia	Gynaecology	34	F	Analgesic	nr	PRE, PER
Kiss	2011	France	Ophthalmology	73	M	LA	nr	PRE, PER, POST
O'Shea	2011	UK	Orthopaedic	62	M	None	Self-hypnosis	PER
Von Ungern	2009	Australia	Venipuncture	13	M	LA	Anaesthesiologist	PER
Cyna	2007	Australia	Venipuncture	5	M	None	Self-hypnosis	PRE, PER
Mackenzie	2007	Australia	mixed surgeries	6	M	GA	Anaesthesiologist	PRE
Burkle	2005	USA	Breast surgery	67	F	LA	Self-hypnosis	PER
Wain	2004	USA	ENT	31	M	None	Anaesthesiologist	PRE, PER
Simon	2001	USA	Lumbar puncture	61	F	LA	Hypnotherapist	PRE, PER
Botta	1999	USA	Plastic	NS	M	None	Self-hypnosis	PER
Finer	1973	Sweden	Percutaneous	nr	F	None	nr	PER
Mason	1955	UK	Breast surgery	24	F	None	Anaesthesiologist	PER

M: Male; F: Female; nr: not reported; LA: local anaesthesia; LR: locoregional; GA: general anaesthesia; ENT: Ear Nose Throat; PRE: pre-intervention; PER: per-intervention; POST: post-intervention.

Appendix S6: Bibliography of included articles.

RCTs:

-Azam MA, Weinrib AZ, Slepian PM et al. Effects of perioperative clinical hypnosis on heart rate variability in patients undergoing oncologic surgery: secondary outcomes of a randomized controlled trial. Frontiers in Pain Research 2024; **5**. https://doi.org/10.3389/fpain.2024.1354015

-Catalano Chiuvé S, Momjian S, Wolff A, Corniola MV. Effectiveness and reliability of hypnosis in stereotaxy: a randomized study. Acta Neurochirurgica 2024; **166**: 112. https://doi.org/10.1007/s00701-024-05943-0

-Polomeni M-M, Huguet T, Mariotti M et al. Avoiding pain during propofol injection in pediatric anesthesia: Hypnoanalgesia of the hand versus intravenous lidocaine. Paediatric Anaesthesia 2024; **34**: 742–9. https://doi.org/10.1111/pan.14909

-Rosenbloom B, Slepian PM, Azam MA et al. A Randomized Controlled Trial of Clinical Hypnosis as an Opioid-Sparing Adjunct Treatment for Pain Relief in Adults Undergoing Major Oncologic Surgery. Journal of Pain Research 2024; **17**: 45–59. https://doi.org/10.2147/JPR.S424639

-Sola C, Devigne J, Bringuier S et al. Hypnosis as an alternative to general anaesthesia for paediatric superficial surgery: a randomised controlled trial. British Journal of Anaesthesia 2023; **130**: 314–21. https://doi.org/10.1016/j.bja.2022.11.023

-Courtois-Amiot P, Cloppet-Fontaine A, Poissonnet A et al. Hypnosis for pain and anxiety management in cognitively impaired older adults undergoing scheduled lumbar punctures: a randomized controlled pilot study. Alzheimer's Research & Therapy 2022; **14**: 120. https://doi.org/10.1186/s13195-022-01065-w

-Markovits J, Blaha O, Zhao E, Spiegel D. Effects of hypnosis versus enhanced standard of care on postoperative opioid use after total knee arthroplasty: the HYPNO-TKA randomized clinical trial. Regional Anesthesia and Pain Medicine 2022: rapm-2022-103493. https://doi.org/10.1136/rapm-2022-103493

-Meyer L, Iniesta-Donate M, Munsell M et al. HERO-Trial: Integration of self-hypnosis in an enhanced recovery after surgery program: A prospective randomized trial (115). Gynecologic Oncology 2022; **166**: S75. https://doi.org/10.1016/S0090-8258(22)01341-5

-Michel-Cherqui M, Szekely B, Fessler J et al. Feasibility and Usefulness of Self-Hypnosis in Patients Undergoing Double-Lung Transplantation During the Pre- and Postoperative Periods: A Randomized Study. Journal of Cardiothoracic and Vascular Anesthesia 2022; **36**: 2490–9. https://doi.org/10.1053/j.jvca.2022.01.027

-Moreno Hernández D, Téllez A, Sánchez-Jáuregui T, García CH, García-Solís M, Valdez A. Clinical Hypnosis For Pain Reduction In Breast Cancer Mastectomy: A Randomized Clinical Trial. International Journal of Clinical and Experimental Hypnosis 2022; **70**: 4–15. https://doi.org/10.1080/00207144.2022.2003697

-Portel L, Perel A, Masson L, Roy C, Mebs S. Tolerance's improvement of flexible bronchoscopy by Ericksonian hypnosis: The BREATH study. Respiratory Medicine and Research 2022; **81**: 100798. https://doi.org/10.1016/j.resmer.2020.100798

-Rousseaux F, Dardenne N, Massion PB et al. Virtual reality and hypnosis for anxiety and pain management in intensive care units: A prospective randomised trial among cardiac surgery patients. European Journal of Anaesthesiology 2022; **39**: 58–66. https://doi.org/10.1097/EJA.0000000000001633

-Viegas J, Holtby H, Runeckles K, Lang EV. The Impact of Scripted Self-Hypnotic Relaxation on the Periprocedural Experience and Anesthesiologist Sedation Use in the Pediatric Cardiac Catheterization Suite: A Prospective Randomized Controlled Trial. Journal of Pain Research 2022; **15**: 3447–58. https://doi.org/10.2147/JPR.S373608

-Sourzac J, Germain C, Frison E, Sztark F, Conri V, Floccia M. Effect of a hypnosis session before hysterectomy on pre- and postoperative anxiety. International Journal of Gynecology & Obstetrics 2021; **155**: 156–7. https://doi.org/10.1002/ijgo.13800

-Tezcan B, Ademoğlu D, Can M et al. A Randomized Clinical Trial on the Effect of Hypnosis on Anxiety and Pain in Rigid Cystoscopy Patients. Journal of Endourology 2021; **35**: 47–53. https://doi.org/10.1089/end.2020.0101

-Fusco N, Bernard F, Roelants F et al. Hypnosis and communication reduce pain and anxiety in peripheral intravenous cannulation: Effect of Language and Confusion on Pain During Peripheral Intravenous Catheterization (KTHYPE), a multicentre randomised trial. British Journal of Anaesthesia 2020; **124**: 292–8. https://doi.org/10.1016/j.bja.2019.11.020

-Garcia R, Bouleti C, Li A et al. Hypnosis Versus Placebo During Atrial Flutter Ablation. JACC: Clinical Electrophysiology 2020; **6**: 1551–60. https://doi.org/10.1016/j.jacep.2020.05.028

-Mubarokah RI, Prasetya H, Respati SH. The Effectiveness of Hypnotherapy to Reduce Anxiety in Pre-Caesarean Section Women. Journal of Maternal and Child Health 2020; **5**: 12–8. https://doi.org/10.26911/thejmch.2020.05.01.02

-Nowak H, Zech N, Asmussen S et al. Effect of therapeutic suggestions during general anaesthesia on postoperative pain and opioid use: multicentre randomised controlled trial. BMJ 2020; **371**: m4284. https://doi.org/10.1136/bmj.m4284

-Efsun Ozgunay S, Ozmen S, Karasu D, Yilmaz C, Taymur I. The Effect of Hypnosis on Intraoperative Hemorrhage and Postoperative Pain in Rhinoplasty. International Journal of Clinical and Experimental Hypnosis 2019; **67**: 262–77. https://doi.org/10.1080/00207144.2019.1612670

-Hoslin L, Motamed C, Maurice-Szamburski A, Legoupil C, Pons S, Bordenave L. Impact of hypnosis on patient experience after venous access port implantation. Anaesthesia Critical Care & Pain Medicine 2019; **38**: 609–13. https://doi.org/10.1016/j.accpm.2019.02.013

-Lee JK, Zubaidah JO, Fadhilah ISI, Normala I, Jensen MP. Prerecorded Hypmotic Peri-Surgical Intervention to Alleviate Risk of Chronic Postsurgical Pain in Total Knee

Replacement; a Randomized Controlled Pilot Study. International Journal of Clinical and Experimental Hypnosis 2019; **67**: 217–45. https://doi.org/10.1080/00207144.2019.1580975

-Sánchez-Jáuregui T, Téllez A, Juárez-García D, García CH, García FE. Clinical Hypnosis and Music In Breast Biopsy:A Randomized Clinical Trial. American Journal of Clinical Hypnosis 2019; **61**: 244–57. https://doi.org/10.1080/00029157.2018.1489776

-Amraoui J, Pouliquen C, Fraisse J et al. Effects of a Hypnosis Session Before General Anesthesia on Postoperative Outcomes in Patients Who Underwent Minor Breast Cancer Surgery: The HYPNOSEIN Randomized Clinical Trial. JAMA Network Open 2018; **1**: e181164. https://doi.org/10.1001/jamanetworkopen.2018.1164

-Bataille A, Guirimand A, Szekely B et al. Does a hypnosis session reduce the required propofol dose during closed-loop anaesthesia induction?: A randomised controlled trial. European Journal of Anaesthesiology 2018; **35**: 675–81. https://doi.org/10.1097/EJA.0000000000000751

-Chen X, Yuan R, Chen X et al. Hypnosis intervention for the management of pain perception during cataract surgery. Journal of Pain Research 2018; **11**: 1921–6. https://doi.org/10.2147/JPR.S174490

-Duparc-Alegria N, Tiberghien K, Abdoul H, Dahmani S, Alberti C, Thiollier A-F. Assessment of a short hypnosis in a paediatric operating room in reducing postoperative pain and anxiety: A randomised study. Journal of Clinical Nursing 2018; **27**: 86–91. https://doi.org/10.1111/jocn.13848

-Akgul A, Guner B, Çırak M, Çelik D, Hergünsel O, Bedirhan S. The Beneficial Effect of Hypnosis in Elective Cardiac Surgery: A Preliminary Study. The Thoracic and Cardiovascular Surgeon 2016; **64**: 581–8. https://doi.org/10.1055/s-0036-1580623

-Behnaz F, Solhpour A. To Compare Efficacy of Hypnosis and Intravenous Sedation in Controlling of Important Variables of Vital Signs and Evaluate the Patient Anxiety Before and after Topical Anesthesia in Ophthalmic Surgery. Novelty in Biomedicine 2016; **4**: 93–9. https://doi.org/10.22037/nbm.v4i3.7925

-Corman I, Bouchema Y, Miquel B et al. HypnosIS to faciLitate trans-Esophageal echocardiograPhy Tolerance: The I-SLEPT study. Archives of Cardiovascular Diseases 2016; **109**: 171–7. https://doi.org/10.1016/j.acvd.2015.09.008

-Dogan Y, Eren GA, Tulubas E, Oduncu V, Sahin A, Ciftci S. The effect of sedation during transoesophageal echocardiography on heart rate variability: a comparison of hypnotic sedation with medical sedation. Kardiologia Polska 2016; **74**: 591–7. https://doi.org/10.5603/KP.a2015.0237

-Joudi M, Fathi M, Izanloo A, Montazeri O, Jangjoo A. An Evaluation of the Effect of Hypnosis on Postoperative Analgesia following Laparoscopic Cholecystectomy. International Journal of Clinical and Experimental Hypnosis 2016; **64**: 365–72. https://doi.org/10.1080/00207144.2016.1171113

-Hızlı F, Özcan O, Selvi İ et al. The effects of hypnotherapy during transrectal ultrasound-guided prostate needle biopsy for pain and anxiety. International Urology and Nephrology 2015; **47**: 1773–7. https://doi.org/10.1007/s11255-015-1111-0

-Catoire P, Delaunay L, Dannappel T et al. Hypnosis versus Diazepam for Embryo Transfer: A Randomized Controlled Study. American Journal of Clinical Hypnosis 2013; **55**: 378–86. https://doi.org/10.1080/00029157.2012.747949

-Shenefelt PD. Anxiety Reduction Using Hypnotic Induction and Self-Guided Imagery for Relaxation During Dermatologic Procedures. International Journal of Clinical and Experimental Hypnosis 2013; **61**: 305–18. https://doi.org/10.1080/00207144.2013.784096

-Reinhard J, Heinrich TM, Reitter A, Herrmann E, Smart W, Louwen F. Clinical Hypnosis Before External Cephalic Version. American Journal of Clinical Hypnosis 2012; **55**: 184–92. https://doi.org/10.1080/00029157.2012.665399

-Liossi C, White P, Hatira P. A randomized clinical trial of a brief hypnosis intervention to control venepuncture-related pain of paediatric cancer patients. Pain 2009; **142**: 255–63. https://doi.org/10.1016/j.pain.2009.01.017

-Lang EV, Berbaum KS, Pauker SG et al. Beneficial effects of hypnosis and adverse effects of empathic attention during percutaneous tumor treatment: when being nice does not suffice. Journal of vascular and interventional radiology: JVIR 2008; **19**: 897–905. https://doi.org/10.1016/j.jvir.2008.01.027

-Marc I, Rainville P, Masse B et al. Hypnotic analgesia intervention during first-trimester pregnancy termination: an open randomized trial. American Journal of Obstetrics and Gynecology 2008; **199**: 469.e1-469.e9. https://doi.org/10.1016/j.ajog.2008.01.058

-Schnur JB, Bovbjerg DH, David D et al. Hypnosis decreases presurgical distress in excisional breast biopsy patients. Anesthesia and Analgesia 2008; **106**: 440–4, table of contents. https://doi.org/10.1213/ane.0b013e31815edb13

-Marc I, Rainville P, Verreault R, Vaillancourt L, Masse B, Dodin S. The use of hypnosis to improve pain management during voluntary interruption of pregnancy: an open randomized preliminary study. Contraception 2007; **75**: 52–8. https://doi.org/10.1016/j.contraception.2006.07.012

-Montgomery GH, Bovbjerg DH, Schnur JB et al. A Randomized Clinical Trial of a Brief Hypnosis Intervention to Control Side Effects in Breast Surgery Patients. JNCI Journal of the National Cancer Institute 2007; **99**: 1304–12. https://doi.org/10.1093/jnci/djm106

-Liossi C, White P, Hatira P. Randomized clinical trial of local anesthetic versus a combination of local anesthetic with self-hypnosis in the management of pediatric procedure-related pain. Health Psychology 2006; **25**: 307–15. https://doi.org/10.1037/0278-6133.25.3.307

-Saadat H, Drummond-Lewis J, Maranets I et al. Hypnosis Reduces Preoperative Anxiety in Adult Patients: Anesthesia & Analgesia 2006; **102**: 1394–6. https://doi.org/10.1213/01.ane.0000204355.36015.54

-Calipel S, Lucas-Polomeni M, Wodey E, Ecoffey C. Premedication in children: hypnosis versus midazolam. Pediatric Anesthesia 2005; **15**: 275–81. https://doi.org/10.1111/j.1460-9592.2004.01514.x

-Cowan GS, Buffington CK, Cowan GS, Hathaway D. Assessment of the effects of a taped cognitive behavior message on postoperative complications (therapeutic suggestions under anesthesia). Obesity Surgery 2001; **11**: 589–93. https://doi.org/10.1381/09608920160556779

-Defechereux T, Degauque C, Fumal I et al. L'hypnosédation, un nouveau mode d'anesthésie pour la chirurgie endocrinienne cervicale. Étude prospective randomisée. Annales de Chirurgie 2000; **125**: 539–46. https://doi.org/10.1016/S0003-3944(00)00238-8

-Lang EV, Benotsch EG, Fick LJ et al. Adjunctive non-pharmacological analgesia for invasive medical procedures: a randomised trial. The Lancet 2000; **355**: 1486–90. https://doi.org/10.1016/S0140-6736(00)02162-0

-Conlong P, Rees W. The use of hypnosis in gastroscopy: a comparison with intravenous sedation. Postgraduate Medical Journal 1999; **75**: 223–5. https://doi.org/10.1136/pgmj.75.882.223

-Ashton C, Whitworth GC, Seldomridge JA et al. Self-hypnosis reduces anxiety following coronary artery bypass surgery. A prospective, randomized trial. The Journal of Cardiovascular Surgery 1997; **38**: 69–75.

-Enqvist B, Björklund C, Engman M, Jakobsson J. Preoperative hypnosis reduces postoperative vomiting after surgery of the breasts. A prospective, randomized and blinded study. Acta Anaesthesiologica Scandinavica 1997; **41**: 1028–32. https://doi.org/10.1111/j.1399-6576.1997.tb04831.x

-Faymonville EM, Mambourg HP, Joris J et al. Psychological approaches during conscious sedation. Hypnosis versus stress reducing strategies: a prospective randomized study. Pain 1997; **73**: 361–7. https://doi.org/10.1016/S0304-3959(97)00122-X

-Lang EV, Joyce JS, Spiegel D, Hamilton D, Lee KK. Self-hypnotic relaxation during interventional radiological procedures: Effects 012 pain perception and intravenous drug use. International Journal of Clinical and Experimental Hypnosis 1996; **44**: 106–19. https://doi.org/10.1080/00207149608416074

-Williams AR, Hind M, Sweeney BP. The incidence and severity of postoperative nausea and vomiting in patients exposed to positive intra-operative suggestions. Anaesthesia 1994; **49**: 340–2. https://doi.org/10.1111/j.1365-2044.1994.tb14190.x

-Greenleaf M, Fisher S, Miaskowski C, Duhamel K. Hypnotizability and Recovery from Cardiac Surgery. American Journal of Clinical Hypnosis 1992; **35**: 119–28. https://doi.org/10.1080/00029157.1992.10402994

-Weinstein EJ, Au PK. Use of Hypnosis before and during Angioplasty. American Journal of Clinical Hypnosis 1991; **34**: 29–37. https://doi.org/10.1080/00029157.1991.10402957

-Goldmann L, Ogg TW, Levey AB. Hypnosis and daycase anaesthesia: A study to reduce pre-operative anxiety and intra-operative anaesthetic requirements. Anaesthesia 1988; **43**: 466–9. https://doi.org/10.1111/j.1365-2044.1988.tb06633.x

-Hart RR. The influence of a taped hypnotic induction treatment procedure on the recovery of surgery patients. International Journal of Clinical and Experimental Hypnosis1980 Oct 1; https://doi.org/10.1080/00207148008409861

-Field PB. Effects of tape-recorded hypnotic preparation for surgery. International Journal of Clinical and Experimental Hypnosis 1974; **22**: 54–61. https://doi.org/10.1080/00207147408412984

NRCSs:

-Berliere M, Roelants F, Duhoux FP et al. Hypnosis Sedation Used in Breast Oncologic Surgery Significantly Decreases Perioperative Inflammatory Reaction. Cancers 2024; **17**: 49. https://doi.org/10.3390/cancers17010049

-Cossu G, Vandenbulcke A, Zaccarini S et al. Hypnosis-Assisted Awake Craniotomy for Eloquent Brain Tumors: Advantages and Pitfalls. Cancers 2024; **16**: 1784. https://doi.org/10.3390/cancers16091784

-Derycke L, De Roux Q, Mongardon N et al. Hypnosis during Endovascular Abdominal Aortic Aneurysm Repair. Journal of Clinical Medicine 2024; **13**: 979. https://doi.org/10.3390/jcm13040979

-Bankole NDA, Kanmounye US, Ouahabi AE, Zemmoura I. Asleep-awake-asleep versus hypnosis for low-grade glioma surgery: long term follow-up outcome. Neurochirurgie 2023; **69**: 101494. https://doi.org/10.1016/j.neuchi.2023.101494

-Loseto N, Zenati N, Seinturier C, Blaise S. Evaluation of patients' and practitionners' satisfaction with the use of hypnosis during a thermal endovenous procedure. JMV-Journal de Médecine Vasculaire 2022; **47**: 82–6. https://doi.org/10.1016/j.jdmv.2022.04.001

-Badidi G, Baulieu M, Vercherin P, De Pasquale V, Gavid M, Prades JM. Thyroid surgery under hypnosis: A 50-case series. European Annals of Otorhinolaryngology, Head and Neck Diseases 2021; **138**: 13–7. https://doi.org/10.1016/j.anorl.2020.06.010

-Juana María PP, Marcelino SC, Manuel QD, Jean Marc B, Francisco Javier EA. Effectiveness of Hypnoanalgesia in Paediatric Dermatological Surgery. Children 2021; **8**: 1195. https://doi.org/10.3390/children8121195

-Scaglione M, Battaglia A, Lamanna A et al. Adjunctive hypnotic communication for analgosedation in subcutaneous implantable cardioverter defibrillator implantation. A prospective single center pilot study. IJC Heart & Vasculature 2021; **35**: 100839. https://doi.org/10.1016/j.ijcha.2021.100839

-Pesce A, Palmieri M, Cofano F et al. Standard awake surgery versus hypnosis aided awake surgery for the management of high grade gliomas: A non-randomized cohort

comparison controlled trial. Journal of Clinical Neuroscience 2020; **77**: 41–8. https://doi.org/10.1016/j.jocn.2020.05.047

-Takahashi M, Mouillet G, Khaled A et al. Perioperative Outcomes of Adjunctive Hypnotherapy Compared with Conscious Sedation Alone for Patients Undergoing Transfemoral Transcatheter Aortic Valve Implantation. International Heart Journal 2020; **61**: 60–6. https://doi.org/10.1536/ihj.19-296

-Touzé A-S, Fournier E, Laffon M, Morinière S. Primary hyperparathyroid surgery under local anaesthesia: benefits of hypnosis. European Archives of Oto-Rhino-Laryngology 2020; **277**: 887–92. https://doi.org/10.1007/s00405-019-05754-5

-Chapet O, Udrescu C, Horn S et al. Prostate brachytherapy under hypnosedation: A prospective evaluation. Brachytherapy 2019; **18**: 22–8. https://doi.org/10.1016/j.brachy.2018.10.004

-Lacroix C, Duhoux FP, Bettendorff J et al. Impact of Perioperative Hypnosedation on Postmastectomy Chronic Pain: Preliminary Results. Integrative Cancer Therapies 2019; **18**: 153473541986949. https://doi.org/10.1177/1534735419869494

-Berlière M, Roelants F, Watremez C et al. The advantages of hypnosis intervention on breast cancer surgery and adjuvant therapy. The Breast 2018; **37**: 114–8. https://doi.org/10.1016/j.breast.2017.10.017

-Boselli E, Musellec H, Bernard F et al. Effects of Conversational Hypnosis on Relative Parasympathetic Tone and Patient Comfort During Axillary Brachial Plexus Blocks for Ambulatory Upper Limb Surgery. A Quasiexperimental Pilot Study. The International Journal of Clinical and Experimental Hypnosis 2018; **66**: 134–46. https://doi.org/10.1080/00207144.2018.1421355

-Manworren RCB, Anderson MN, Girard ED et al. Postoperative Pain Outcomes After Nuss Procedures: Comparison of Epidural Analgesia, Continuous Infusion of Local Anesthetic, and Preoperative Self-Hypnosis Training. Journal of Laparoendoscopic & Advanced Surgical Techniques 2018; **28**: 1234–42. https://doi.org/10.1089/lap.2017.0699

-Zech N, Seemann M, Seyfried TF, Lange M, Schlaier J, Hansen E. Deep Brain Stimulation Surgery without Sedation. Stereotactic and Functional Neurosurgery 2018; **96**: 370–8. https://doi.org/10.1159/000494803

-Agard E, Pernod C, El Chehab H, Russo A, Haxaire M, Dot C. Apport de l'hypnose dans la chirurgie de cataracte, à propos de 171 chirurgies. Journal Français d'Ophtalmologie 2016; **39**: 287–91. https://doi.org/10.1016/j.jfo.2015.04.024

-Romain B, Rodriguez M, Story F, Delhorme J-B, Brigand C, Rohr S. Outcomes of hypnosis combined with local anesthesia during inguinal repair: a pilot study. Hernia 2017; **21**: 59–63. https://doi.org/10.1007/s10029-016-1521-7

-Zemmoura I, Fournier E, El-Hage W, Jolly V, Destrieux C, Velut S. Hypnosis for Awake Surgery of Low-grade Gliomas: Description of the Method and Psychological Assessment. Neurosurgery 2016; **78**: 53–61. https://doi.org/10.1227/NEU.0000000000000993

-Eren G, Dogan Y, Demir G et al. Hypnosis for sedation in transesophageal echocardiography: a comparison with midazolam. Annals of Saudi Medicine 2015; **35**: 58–63. https://doi.org/10.5144/0256-4947.2015.58

-Coveney E, Grieve M, Kumar B. Impact of therapeutic hypnosis on pain and anxiety in patients undergoing breast cancer surgery. European Journal of Surgical Oncology (EJSO) 2011; **37**: 1002. https://doi.org/10.1016/j.ejso.2011.08.103

-Gauchotte E, Masias C, Bogusz N, Koebele A. Stérilisation tubaire par voie hystéroscopique avec le système Essure® : étude descriptive et évaluation de l'efficacité de l'hypnose. Journal de Gynécologie Obstétrique et Biologie de la Reproduction 2011; **40**: 305–13. https://doi.org/10.1016/j.jgyn.2011.02.007

-Lew MW, Kravits K, Garberoglio C, Williams AC. Use of Preoperative Hypnosis to Reduce Postoperative Pain and Anesthesia-Related Side Effects. International Journal of Clinical and Experimental Hypnosis 2011; **59**: 406–23. https://doi.org/10.1080/00207144.2011.594737

-Musellec H, Bernard F, Houssel P et al. Étude prospective comparant l'hypnosédation et l'anesthésie générale pour la pose de dispositif de stérilisation intratubaire en ambulatoire. *Annales Françaises d'Anesthésie et de Réanimation* 2010; **29**: 889–96. https://doi.org/10.1016/j.annfar.2010.10.008

-Novoa R, Hammonds T. Clinical hypnosis for reduction of atrial fibrillation after coronary artery bypass graft surgery. Cleveland Clinic Journal of Medicine 2008; **75 Suppl 2**: S44-47. https://doi.org/10.3949/ccjm.75.suppl_2.s44

-Bouté V, Halfon Y, Gagean F. Intérêt de l'hypnose en sénologie. Psycho-Oncologie 2007; **1**: 105–18. https://doi.org/10.1007/s11839-007-0023-3

-Elkins G, White J, Patel P, Marcus J, Perfect MM, Montgomery GH. Hypnosis to manage anxiety and pain associated with colonoscopy for colorectal cancer screening: Case studies and possible benefits. The International Journal of Clinical and Experimental Hypnosis 2006; **54**: 416–31. https://doi.org/10.1080/00207140600856780

-Levitas E, Parmet A, Lunenfeld E et al. Impact of hypnosis during embryo transfer on the outcome of in vitro fertilization–embryo transfer: a case-control study. Fertility and Sterility 2006; **85**: 1404–8. https://doi.org/10.1016/j.fertnstert.2005.10.035

-Lobe TE. Perioperative Hypnosis Reduces Hospitalization in Patients Undergoing the Nuss Procedure for Pectus Excavatum. Journal of Laparoendoscopic & Advanced Surgical Techniques 2006; **16**: 639–42. https://doi.org/10.1089/lap.2006.16.639

-Defechereux T, Meurisse M, Hamoir E, Gollogly L, Joris J, Faymonville ME. Hypnoanesthesia for endocrine cervical surgery: a statement of practice. Journal of Alternative and Complementary Medicine (New York, N.Y.) 1999; **5**: 509–20. https://doi.org/10.1089/acm.1999.5.509

-Enqvist B, Von Konow L, Bystedt H. Stress reduction, preoperative hypnosis and perioperative suggestion in maxillofacial surgery: Somatic responses and recovery. Stress Medicine 1995; **11**: 229–33. https://doi.org/10.1002/smi.2460110138

-Faymonville ME, Fissette J, Mambourg PH, Roediger L, Joris J, Lamy M. Hypnosis as adjunct therapy in conscious sedation for plastic surgery. Regional Anesthesia 1995; **20**: 145–51.

Uncontrolled studies of more than one patient:

-Bobin F, Garreau C, Lechien JR. Safety and Feasibility of Hypnosis-Induced Sleep Endoscopy in Obstructive Sleep Apnea Patients. Ear, Nose, & Throat Journal 2023: 1455613231170094. https://doi.org/10.1177/01455613231170094

-Fontanges P-A, De Jonkheere J, Baudelet JB et al. Clinical hypnosis associated with local anesthesia for cardiac catheterization in pediatric population. Archives of Cardiovascular Diseases Supplements 2023; **15**: 141. https://doi.org/10.1016/j.acvdsp.2022.10.271

-Chandrasegaran A, Sundralingam S. Hypnosedation for cataract surgery with ideodynamic hypnotic induction, as an approach: a case series of two patients. Received 2021-09-21; Accepted 2022-02-06; Published 2022-06-22. Journal of Health and Translational Medicine (JUMMEC) 2022; **25**: 154–7.

-Wood C, Martiné G, Espagne-Dubreuilh G et al. The Added Value of Intraoperative Hypnosis during Spinal Cord Stimulation Lead Implantation under Awake Anesthesia in Patients Presenting with Refractory Chronic Pain. Medicina 2022; **58**: 220. https://doi.org/10.3390/medicina58020220

-Lind SB, Jacobsen HB, Solbakken OA, Reme SE. Clinical Hypnosis in Medical Care: A Mixed-Method Feasibility Study. Integrative Cancer Therapies 2021; **20**: 15347354211058678. https://doi.org/10.1177/15347354211058678

-Lopes M, Dridi L, Yvernay R. Use of hypnosis preoperatively for surgery of the spine: Pilot study. Neurochirurgie 2021; **67**: 358–61. https://doi.org/10.1016/j.neuchi.2020.11.012

-Tran LC, Coopman S, Rivallain C et al. Use of Hypnosis in Paediatric Gastrointestinal Endoscopy: A Pilot Study. Frontiers in Pediatrics 2021; **9**: 719626. https://doi.org/10.3389/fped.2021.719626

-Jaouen M, Parent V, Codet M et al. Neck Surgery with Hypnosis: An Evaluation Based Upon Patient's Self Assessment. Annals of Otology, Rhinology & Laryngology 2020; **129**: 256–64. https://doi.org/10.1177/0003489419882445

-Kissel M, Andraud M, Duhamel A-S et al. Hypnosedation for endocavitary uterovaginal applications: A pilot study. Brachytherapy 2020; **19**: 462–9. https://doi.org/10.1016/j.brachy.2020.03.008

-Amedro P, Gavotto A, Gelibert D et al. Feasibility of clinical hypnosis for transesophageal echocardiography in children and adolescents. European Journal of Cardiovascular Nursing 2019; **18**: 163–70. https://doi.org/10.1177/1474515118803513

-Fathi M, Ariamanesh AS, Joudi M, Joudi M, Sadrossadati F, Izanloo A. Hypnosis as an Approach to Control Pain and Anxiety in Anterior Cruciate Ligament Reconstruction and Meniscal Surgeries: Two Case Presentations. Anesthesiology and Pain Medicine 2019; **9**. https://doi.org/10.5812/aapm.89277

-Barbero U, Ferraris F, Muro M, Budano C, Anselmino M, Gaita F. Hypnosis as an effective and inexpensive option to control pain in transcatheter ablation of cardiac arrhythmias: Journal of Cardiovascular Medicine 2018; **19**: 18–21. https://doi.org/10.2459/JCM.0000000000000605

-Sterkers N, Chabrol JL, De Troyer J, Bonijol D, Darmon JC, Donnez O. Hypnosis as adjunct therapy to conscious sedation for venous access device implantation in breast cancer: A pilot study. The Journal of Vascular Access 2018; **19**: 382–6. https://doi.org/10.1177/1129729818757975

-Claude L, Morelle M, Mancini S et al. Radiothérapie des enfants de moins de cinq ans : peut-on limiter les anesthésies itératives par les rituels et l'hypnose ? Bulletin du Cancer 2016; **103**. https://doi.org/10.1016/j.bulcan.2016.09.012

-Bouzinac A, Delbos A, Mazières M, Rontes O, Manenc J-L. Hypnose et bloc paravertébral échoguidé dans la chirurgie du cancer du sein. Annales Françaises d'Anesthésie et de Réanimation 2012; **31**: 644–5. https://doi.org/10.1016/j.annfar.2012.03.011

-Galy M, Anidjar S. Hypnoanalgesy in carotid surgery: a team approach. Sang thrombose vaisseaux 2012; **24**: 365–70. https://doi.org/10.1684/stv.2012.0729

-Domínguez-Ortega L, Rodríguez-Muñoz S. The effectiveness of clinical hypnosis in the digestive endoscopy: a multiple case report. The American Journal of Clinical Hypnosis 2010; **53**: 101–7. https://doi.org/10.1080/00029157.2010.10404332

-Hermes D, Truebger D, Hakim SG, Sieg P. Tape recorded hypnosis in oral and maxillofacial surgery—basics and first clinical experience. Journal of Cranio-Maxillofacial Surgery 2005; **33**: 123–9. https://doi.org/10.1016/j.jcms.2004.06.009

-Séfiani T, Uscain M, Sany J-L et al. Cœliochirurgie sous anesthésie locale et hypnosédation, à propos de 35 cholécystectomies et 15 cures de hernies. Annales Françaises d'Anesthésie et de Réanimation 2004; **23**: 1093–101. https://doi.org/10.1016/j.annfar.2004.08.014

-Bertoni F, Bonardi A, Magno L et al. Hypnosis instead of general anaesthesia in paediatric radiotherapy: report of three cases. Radiotherapy and Oncology: Journal of the European Society for Therapeutic Radiology and Oncology 1999; **52**: 185–90. https://doi.org/10.1016/s0167-8140(99)00082-1

-Adams PC, Stenn PG. Liver biopsy under hypnosis. Journal of Clinical Gastroenterology 1992; **15**: 122–4. https://doi.org/10.1097/00004836-199209000-00008

-Crawford AH, Jones CW, Perisho JA, Herring JA. Hypnosis for monitoring intraoperative spinal cord function. Anesthesia and Analgesia 1976; **55**: 42–4. https://doi.org/10.1213/00000539-197601000-00009

Case reports:

-Bapteste L, Marchal M, Szostek A-S, Guenot M, Beuriat P-A. A successful awake pediatric neurosurgery procedure following, 3 months after, a previous failure due to an emergence agitation: a case report. Child's Nervous System 2023; **39**: 1353–6. https://doi.org/10.1007/s00381-023-05840-4

-Oung C, Tesoro R, Marti V, Mavromati M, Lahoud M-J. Hypnosis in High-Intensity Focused Ultrasound for Thyroid Nodule Ablation. American Journal of Case Reports 2023; **24**. https://doi.org/10.12659/AJCR.941524

-Chandrasegaran A, Leong AH. Clinical hypnosis as an adjunct in anesthesia for a surgical procedure: Received 2021-10-05; Accepted 2022-08-05; Published 2023-01-15. Journal of Health and Translational Medicine (JUMMEC) 2023; **26**: 12–5. https://doi.org/10.22452/jummec.vol26no1.4

-Pilia E, Sirigu D, Mereu R, Zamboni F, Pusceddu E. A clinical operative sequence for hypnosis implementation to general anesthesia during major surgery for orthotopic liver transplantation. Annals of Medicine & Surgery 2022; **80**. https://doi.org/10.1016/j.amsu.2022.104345

-Fathi M, Sabermoghaddam A, Azaraein MH, Joudi M, GharaviFard M, Moghaddam SK. Hypnotic Anesthesia for Blepharoplasty Surgery: A Case Report. Archives of Neuroscience 2021; **8**. https://doi.org/10.5812/ans.101882

-Vanreusel I, Maes S, De Wolf D, Van Berendoncks A. Percutaneous closure of a secundum atrial septum defect performed under hypnosis: a case report. Acta Cardiologica 2021; **76**: 1078–82. https://doi.org/10.1080/00015385.2020.1822491

-Makovac P, Potié A, Roukain A et al. Hypnosis and superficial cervical anesthesia for total thyroidectomy in a high-risk patient – A case report. International Journal of Surgery Case Reports 2020; **72**: 133–6. https://doi.org/10.1016/j.ijscr.2020.05.078

-Ibañez Del Prado C, Cruzado JA, González Ordi H, Capilla Ramírez P. Use of hypnosis for the placement of a midline catheter in a patient at the end of life. Palliative & Supportive Care 2020; **18**: 113–7. https://doi.org/10.1017/S1478951519000579

-Al-Nasser B, Lucas-Gourdet E. Thyroid surgery under bilateral superficial cervical block and hypnosis. Minerva Anestesiologica 2018; **84**. https://doi.org/10.23736/S0375-9393.17.12385-0

-Cholet C, Ben Abdallah I, Khaled A, Dhonneur G, Kobeiter H, Desgranges P. Complex Endovascular Abdominal Aneurysm Repair with Fenestrated Endograft Insertion under Hypnosis and Local Anesthesia. Journal of Vascular and Interventional Radiology 2017; **28**: 1289–91. https://doi.org/10.1016/j.jvir.2017.05.021

-Fathi M, Aziz Mohammadi S, Moslemifar M et al. Hypnoanalgesia for Dilatation and Curettage Pain Control. Anesthesiology and Pain Medicine 2017; **7**. https://doi.org/10.5812/aapm.44628

-Fuzier R, Achelous S, Salvignol G, Jouve E. Hypnosis and Axillary Compartment Block for Breast Cancer Surgery: A Case Report. A & A Case Reports 2017; 9: 81–3. https://doi.org/10.1213/XAA.0000000000000532

-Fiddaman J. Simple Mastectomy under Hypnosis: A Case Study Approach. Journal of Perioperative Practice 2016; **26**: 217–20. https://doi.org/10.1177/175045891602601001

-Bienvenu M, Menrath S, Dugué S et al. Amputation et hypnose : exemple de coopération interdisciplinaire autour d'un cas clinique (podcast). Le Praticien en Anesthésie Réanimation 2015; **19**: 49–53. https://doi.org/10.1016/j.pratan.2014.12.009

-Antonelli C, Luchetti M, De Trana L. The Association of Hypno-Anesthesia and Conventional Anesthesia in a Patient With Multiple Allergies at Risk of Anaphylactic Shock. International Journal of Clinical and Experimental Hypnosis 2014; **62**: 483–91. https://doi.org/10.1080/00207144.2014.931178

-Facco E, Pasquali S, Zanette G, Casiglia E. Hypnosis as sole anaesthesia for skin tumour removal in a patient with multiple chemical sensitivity. Anaesthesia 2013; **68**: 961–5. https://doi.org/10.1111/anae.12251

-Wong L, Cyna AM, Matthews G. Rapid hypnosis as an anaesthesia adjunct for evacuation of postpartum vulval haematoma: Adjunctive hypnosis for evacuation of vulval haematoma. Australian and New Zealand Journal of Obstetrics and Gynaecology 2011; **51**: 265–7. https://doi.org/10.1111/j.1479-828X.2011.01310.x

-Kiss G, Butler J. Hypnosis for cataract surgery in an American Society of Anesthesiologists physical status IV patient. Anaesthesia and Intensive Care 2011; **39**: 1139–41. https://doi.org/10.1177/0310057X1103900626

-O'Shea J, Dodd L, Panayiotou S, Palmer S. Self-induced hypnosis for bilateral ankle arthroscopy. British Journal of Anaesthesia 2011; **106**: 282. https://doi.org/10.1093/bja/aeq387

-Von Ungern-Sternberg BS, Habre W. Hypnosis as an alternative to avoid general anesthesia in a child with severe pulmonary arterial hypertension. Pediatric Anesthesia 2009; **19**: 182–3. https://doi.org/10.1111/j.1460-9592.2008.02800.x

-Cyna AM, Tomkins D, Maddock T, Barker D. Brief hypnosis for severe needle phobia using switch--wire imagery in a 5-year old. Pediatric Anesthesia 2007; **17**: 800–4. https://doi.org/10.1111/j.1460-9592.2007.02224.x

-Mackenzie A, Frawley GP. Preoperative Hypnotherapy in the Management of a Child with Anticipatory Nausea and Vomiting. Anaesthesia and Intensive Care 2007; **35**: 784–7. https://doi.org/10.1177/0310057X0703500522

-Burkle CM, Jankowski CJ, Torsher LC, Rho EH, Degnim AC. Bis Monitor Findings During Self-Hypnosis. Journal of Clinical Monitoring and Computing 2005; **19**: 391–3. https://doi.org/10.1007/s10877-005-6539-9

-Wain HJ. Reflections on Hypnotizability and Its Impact on Successful Surgical Hypnosis: A Sole Anesthetic for Septoplasty. American Journal of Clinical Hypnosis 2004; **46**: 313–21. https://doi.org/10.1080/00029157.2004.10403615

-Simon EP, Canonico MM. Use of hypnosis in controlling lumbar puncture distress in an adult needle-phobic dementia patient. The International Journal of Clinical and Experimental Hypnosis 2001; **49**: 56–67. https://doi.org/10.1080/00207140108410379

-Botta SA. Self-Hypnosis as Anesthesia for Liposuction Surgery. American Journal of Clinical Hypnosis 1999; **41**: 299–301. https://doi.org/10.1080/00029157.1999.10404227

-Finer B, Jonzon A, Sedin G, Sjöstrand U. Some Physiological Changes During Minor Surgery Under Hypnotic Analgesia. Acta Anaesthesiologica Scandinavica 1974; **18**: 94–6. https://doi.org/10.1111/j.1399-6576.1974.tb00787.x

-Mason AA. Surgery under hypnosis. Anaesthesia 1955; **10**: 295–9. https://doi.org/10.1111/j.1365-2044.1955.tb01615.x

Appendix S7: Separate Analyses Including NRCS Data

	Timing of hypnosis administration								
	**Pre-intervention (22RCTs & 6 NRCSs)**			**Per-intervention (28 RCTs & 24 NRCSs)**			**Post-intervention (9RCTs & 3 NRCSs)**		
	**RCT/NRCS**			**RCT/NRCS**			**RCT/NRCS**		
	**# studies**	**# patients**	Estimate with 95%CI	**# studies**	**# patients**	Estimate with 95%CI	**# studies**	**# patients**	Estimate with 95%CI
**Primary outcomes**									
Propofol (mg)	2/1	348/65	**-25.2 (-31.9 to -18.6)**	1/3	65/259	**-112.3 (-186.6 to -38.0)**			nr
Any opioid, during the intervention	3/0	266/0	-0.64 (-1.40 to 0.13)*	1/3	113/215	-1.61 (-3.47 to 0.26)*			nr
No need for analgosedation (yes/no)				2/1	377/16	**3.13 [1.77 to 5.55]**			nr
**Secondary outcomes**									
Pain intensity during the intervention (VAS cm)				6/2	811/100	**-1.50 (-2.54 to -0.46)**			nr
Pain intensity after the intervention (VAS cm)	7/2	419/142	**-0.94 (-1.54 to -0.35)**	7/2	927/278	**-0.61 (-1.03 to -0.20)**	2/1	108/337	0.02 (-0.88 to 0.92)
Need for class I-II analgesic (yes/no)	3/1	212/65	**0.43 [0.19 to 0.97]**	1/2	77/258	0.24 [0.04 to 1.29]	0/0	0/0	--
Need for class III analgesic (yes/no)				4/1	340/36	0.71 [0.48 to 1.05]	1/0	50/0	
Anxiety during the intervention (score)				2/1	377/30	-0.47 (-1.20 to 0.26)	1/0	113/0	nr
Anxiety after the intervention (score)	**8/0**	**515**	**-0.76 (-1.14 to -0.38)***	8/3	963/296	**-0.72 (-1.25 to -0.19)***	0/0	0/0	--
Patient satisfaction (VAS cm)				4/2	465/176	0.21 (-0.34 to 0.75)	0/1	337	
Medical team satisfaction (VAS cm)			--	2/2	146/176	1.10 (0.52 to 1.68)	0/0	0/0	nr
Complications (yes/no)				1/6	78/665	0.78 [0.53 to 1.12]	1/1	32/337	
PONV (yes/no)				3/4	185/745	**0.25 [0.13 to 0.49]**	0/0	0/0	--
Procedure duration (min)				7/10	1134/644	-0.56 (-2.26 to 1.15)	0/1	0/337	
Time in PACU (min)	2/1	348/65	**-13.4 (-20.4 to -6.50)**	2/2	137/218	**-47.4 (-66.0 to -28.8)**	0/0	0/0	--
Hospital stay (day)				1/2	59/161	-0.42 (-1.04 to 0.19)	1/1	32/337	

Estimates are reported as WMD(95%CI), SMD(95%CI)*, RR[95%CI].nr: not relevant. In bold: statistically significant results. RCT: randomized controlled trials; NRCS: non-randomized controlled studies; # studies: number of included studies reporting on the outcome; # patients: number of patients in studies reporting on the outcome; Analyses were not performed if fewer than 3 studies reported on a given outcome.

Appendix S8

Figures of quantitative analyses

Pre-intervention hypnosis

Pre-intervention hypnosis

Propofol consumption (mg)

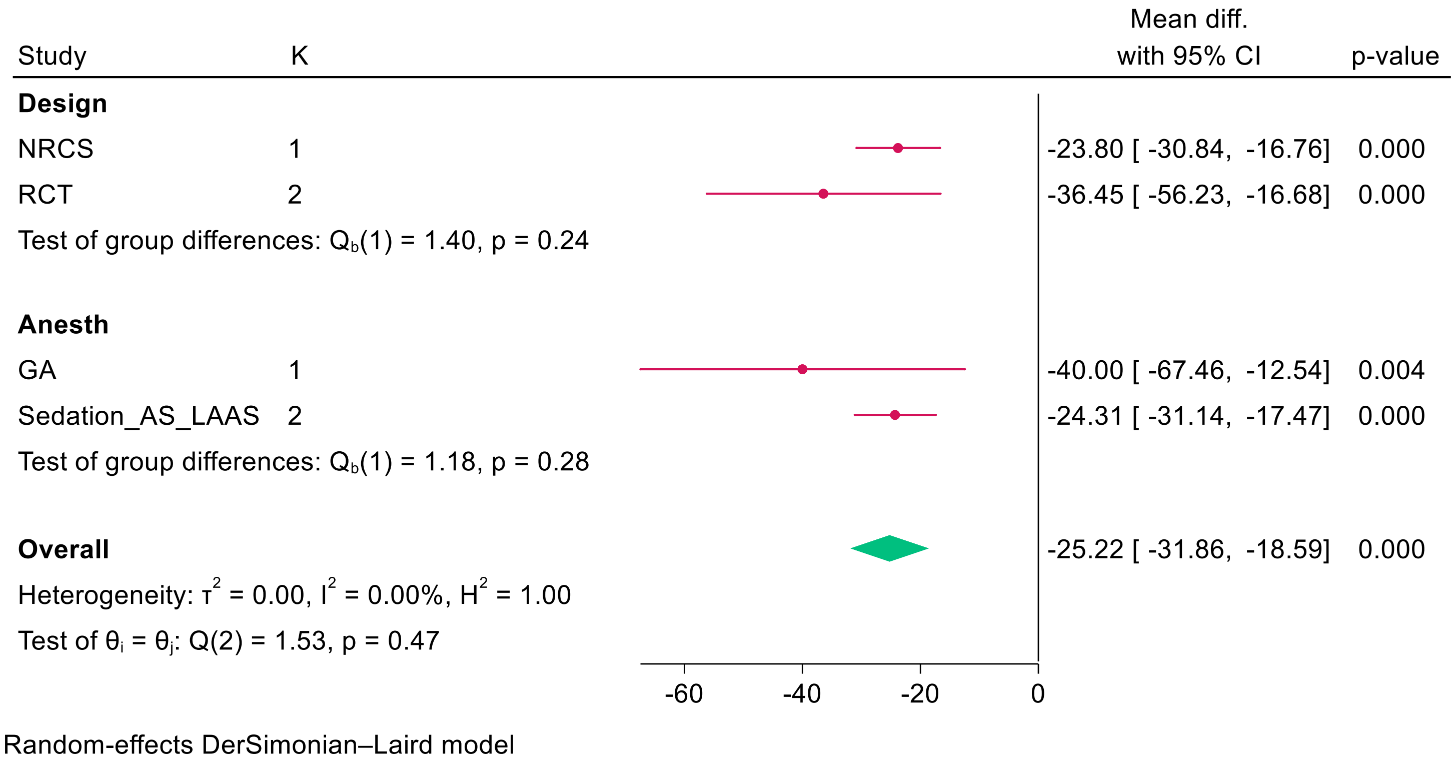

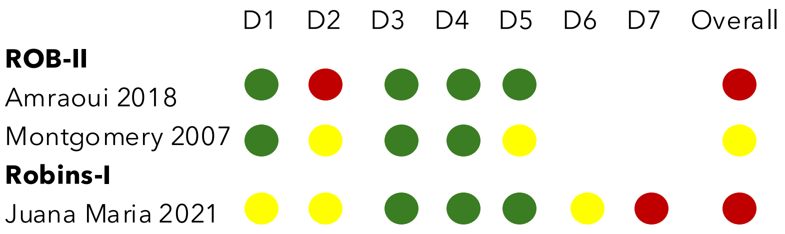

ROB-II : D1-Bias arising from the randomisation process; D2-Bias due to deviations from intended intervention; D3-Bias due to missing outcome data; D4-Bias in Measurement of the outcome; D5- Bias in the selection of the reported results
Robins-I: D1-Bais due to confounding; D2-Bias due to selection of participants; D3-Bias in classification of interventions; D4- Bias due to deviations from intended intervention; D5- Bias due to missing data; D6- Bias due to measurement of outcomes; D7- Bias in the selection of the reported results

Risk of Bias: High Some concern Low

NRCS: Non-randomized controlled studies; RCT: Randomized controlled trials; Anesth: type of anaesthesia; GA: General Anaesthesia; AS: Analgosedation; LAAS: Local anaesthesia with analgosedation

Any opioid, during the intervention

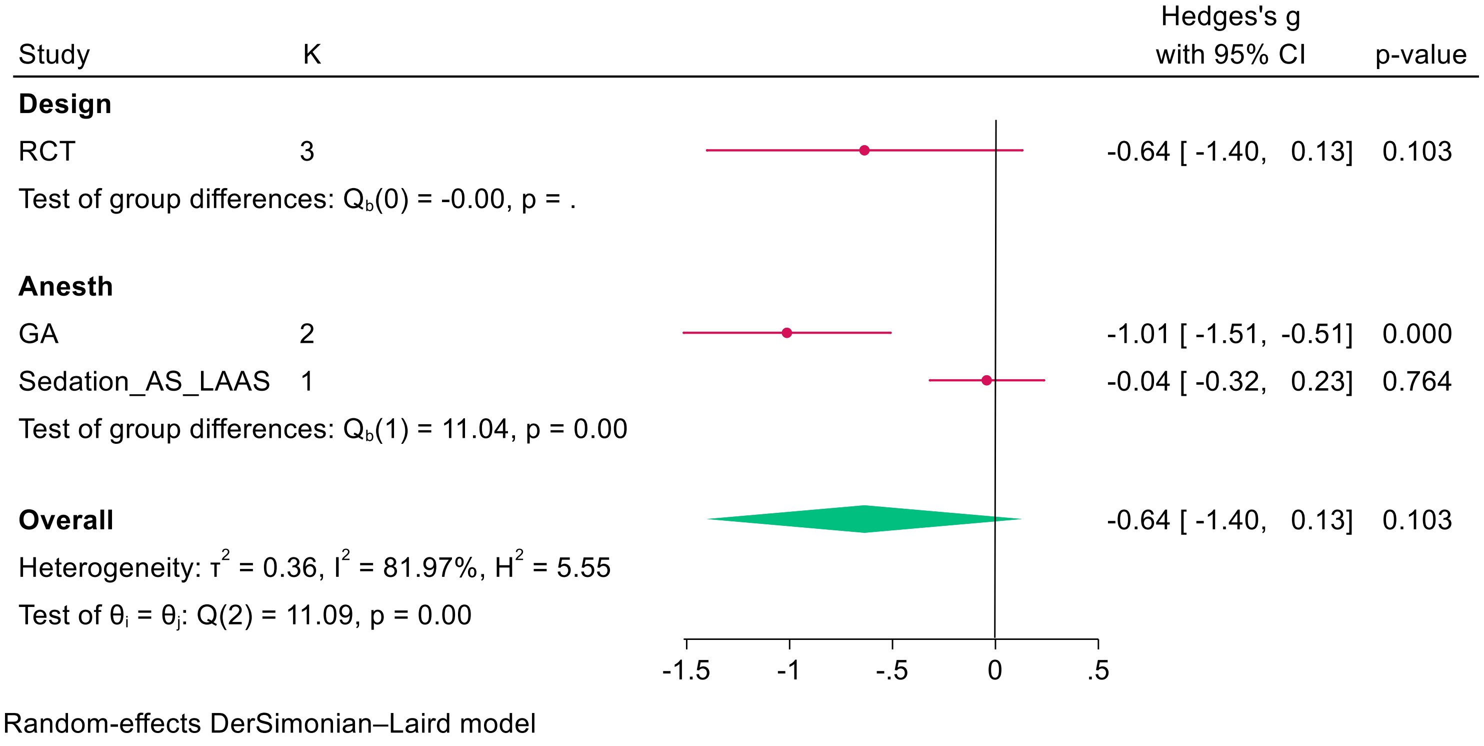

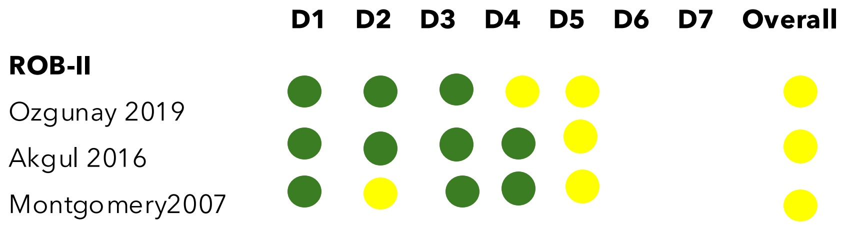

ROB-II : D1-Bias arising from the randomisation process; D2-Bias due to deviations from intended intervention; D3-Bias due to missing outcome data; D4-Bias in Measurement of the outcome; D5- Bias in the selection of the reported results

Risk of Bias: High ● Some concern ● Low ●

RCT: Randomized controlled trials; Anesth: type of anaesthesia; GA: General Anaesthesia; AS: Analgosedation; LAAS: Local anaesthesia with analgosedation

Pre-intervention hypnosis

Pain intensity after the intervention (VAS cm)

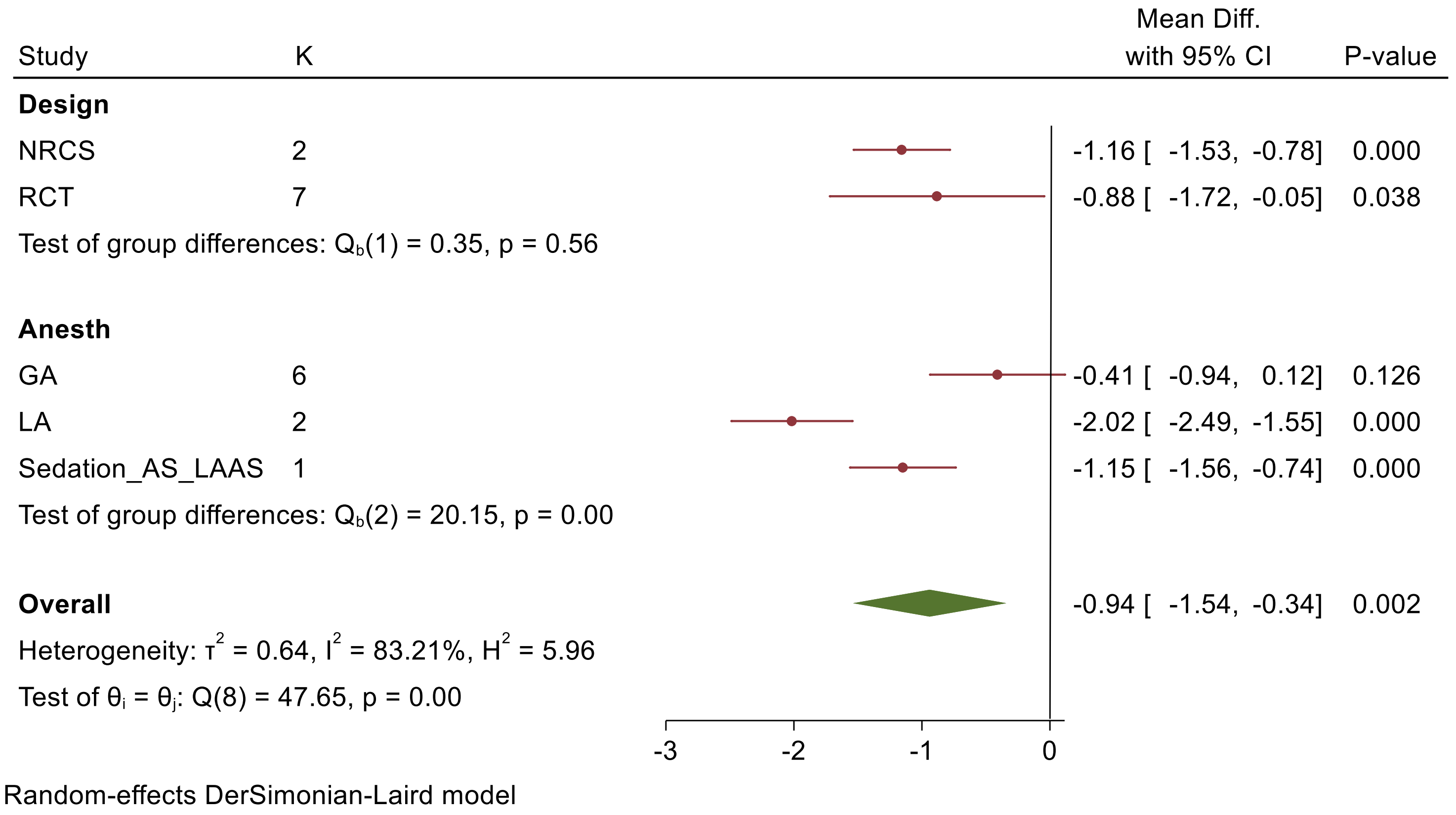

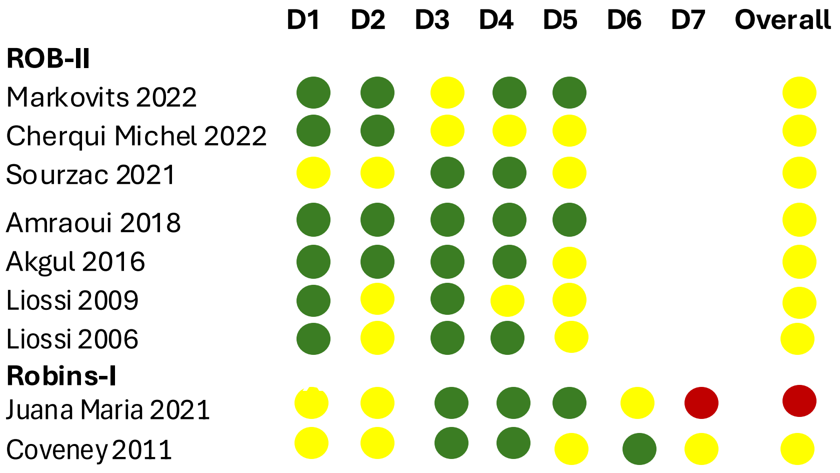

NRCS: Non-randomized controlled studies ; RCT: Randomized controlled trials; Anesth: type of anaesthesia; GA: General Anaesthesia; LA: Local anaesthesia; AS: Analgosedation; LAAS: Local anaesthesia with analgosedation

ROB-II : D1-Bias arising from the randomisation process; D2-Bias due to deviations from intended intervention; D3-Bias due to missing outcome data; D4-Bias in Measurement of the outcome; D5- Bias in the selection of the reported results

Robins-I: D1-Bais due to confounding; D2-Bias due to selection of participants; D3-Bias in classification of interventions; D4- Bias due to deviations from intended intervention; D5- Bias due to missing data; D6- Bias due to measurement of outcomes; D7- Bias in the selection of the reported results

Risk of Bias: High Some concern Low

Pre-intervention hypnosis

Need for class I-II analgesic

Study	K	Risk Ratio with 95% CI	P-value
**Design**			
NRCS	1	0.55 [0.33, 0.93]	0.025
RCT	3	0.30 [0.06, 1.60]	0.159
Test of group differences: $Q_b(1) = 0.47$, $p = 0.49$			
**Anesth**			
GA	3	0.30 [0.06, 1.60]	0.159
Sedation_AS_LAAS	1	0.55 [0.33, 0.93]	0.025
Test of group differences: $Q_b(1) = 0.47$, $p = 0.49$			
**Overall**		0.43 [0.19, 0.97]	0.041
Heterogeneity: $\tau^2 = 0.43$, $I^2 = 77.65\%$, $H^2 = 4.47$			
Test of $\theta_i = \theta_j$: $Q(3) = 13.42$, $p = 0.00$			

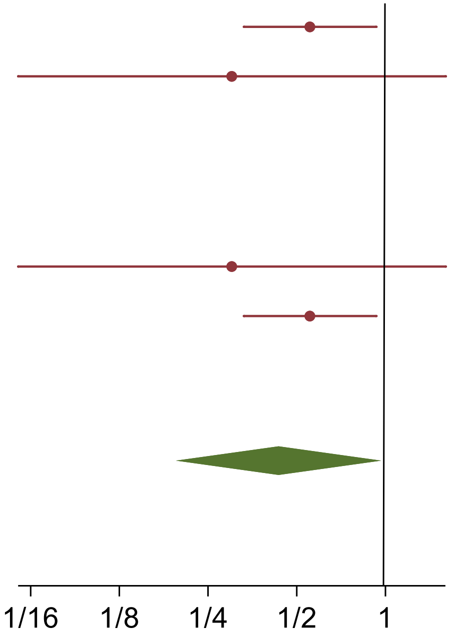

Random-effects DerSimonian-Laird model

NRCS: Non-randomized controlled studies; RCT: Randomized controlled trials; Anesth: type of anaesthesia; GA: General Anaesthesia; AS: Analgosedation; LAAS: Local anaesthesia with analgosedation

	D1	D2	D3	D4	D5	D6	D7	Overall
**ROB-II**								
**General anaesthesia**								
Ozgunay 2019	Low	Low	Low	Some concern	Some concern			Some concern
Amraoui 2018	Low	Some concern	Low	Some concern	Low			Some concern
Akgul 2016	Low	Low	Low	Low	Some concern			Some concern
**Robins-I**								
Juana Maria 2021	Some concern	Some concern	Low	Low	Low	Some concern	High	High

ROB-II : D1-Bias arising from the randomisation process; D2-Bias due to deviations from intended intervention; D3-Bias due to missing outcome data; D4-Bias in Measurement of the outcome; D5- Bias in the selection of the reported results
Robins-I: D1-Bais due to confounding; D2-Bias due to selection of participants; D3-Bias in classification of interventions; D4- Bias due to deviations from intended intervention; D5- Bias due to missing data; D6- Bias due to measurement of outcomes; D7- Bias in the selection of the reported results

Risk of Bias: High, Some concern, Low

Anxiety after the intervention (score)

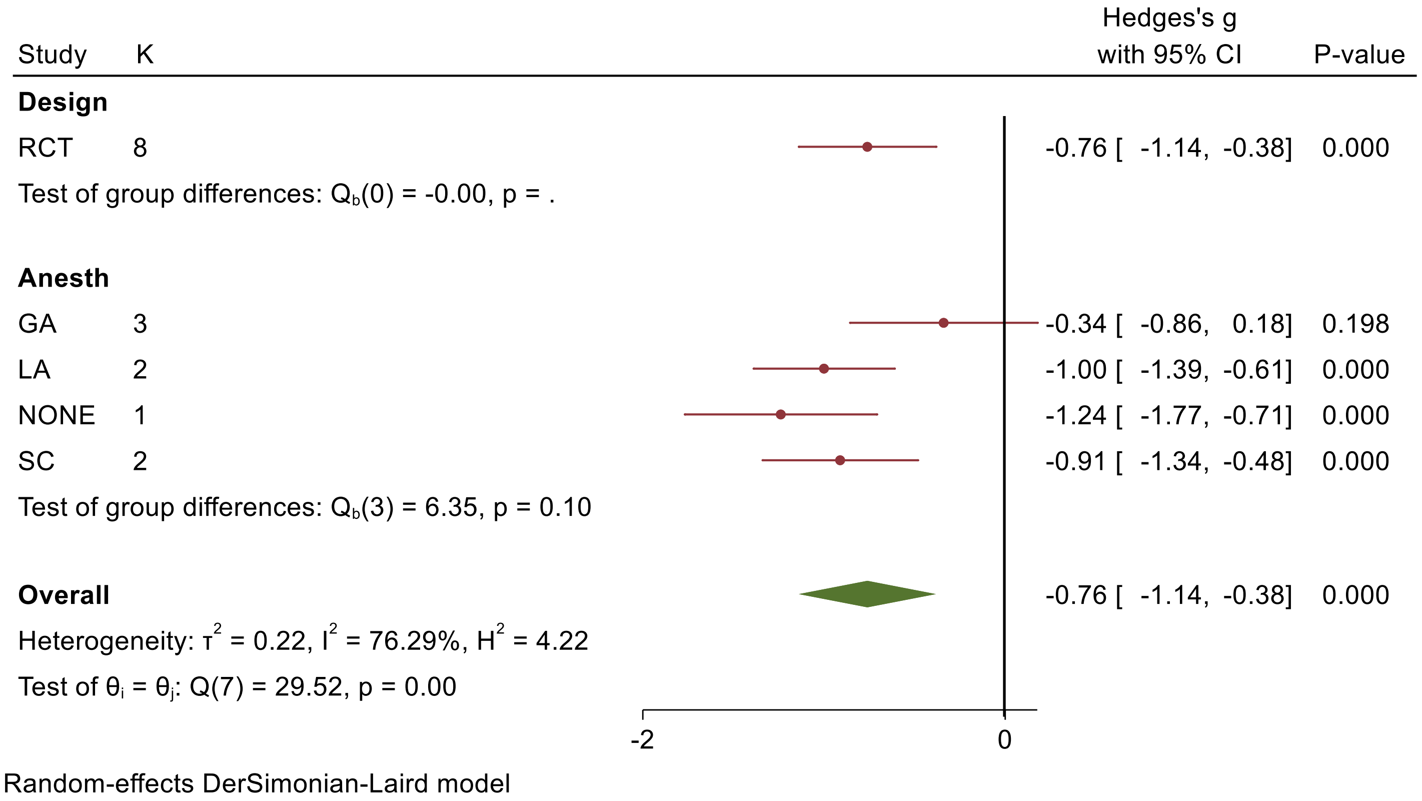

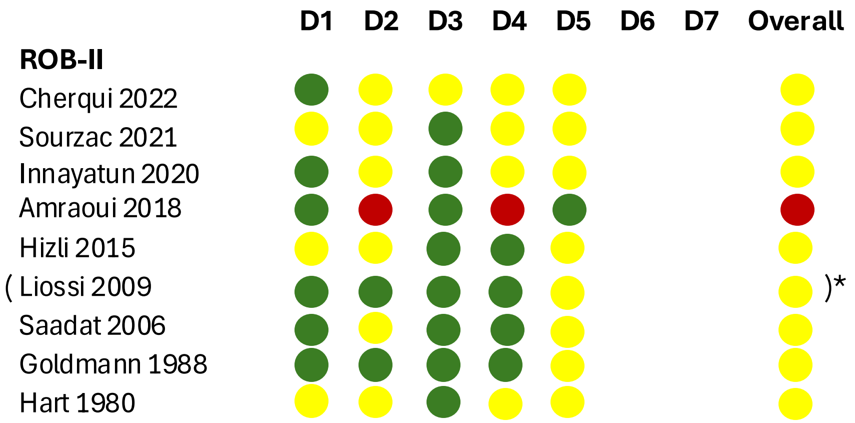

ROB-II : D1-Bias arising from the randomisation process; D2-Bias due to deviations from intended intervention; D3-Bias due to missing outcome data; D4-Bias in Measurement of the outcome; D5- Bias in the selection of the reported results

Risk of Bias: High ● Some concern ● Low ●

*excluded due to an outcome that was markedly different.

RCT: Randomized controlled trials; Anesth: type of anaesthesia; GA: General Anaesthesia; LA: Local anaesthesia: NONE: no anaesthesia; SC: standard care

Pre-intervention hypnosis

Time in PACU (min)

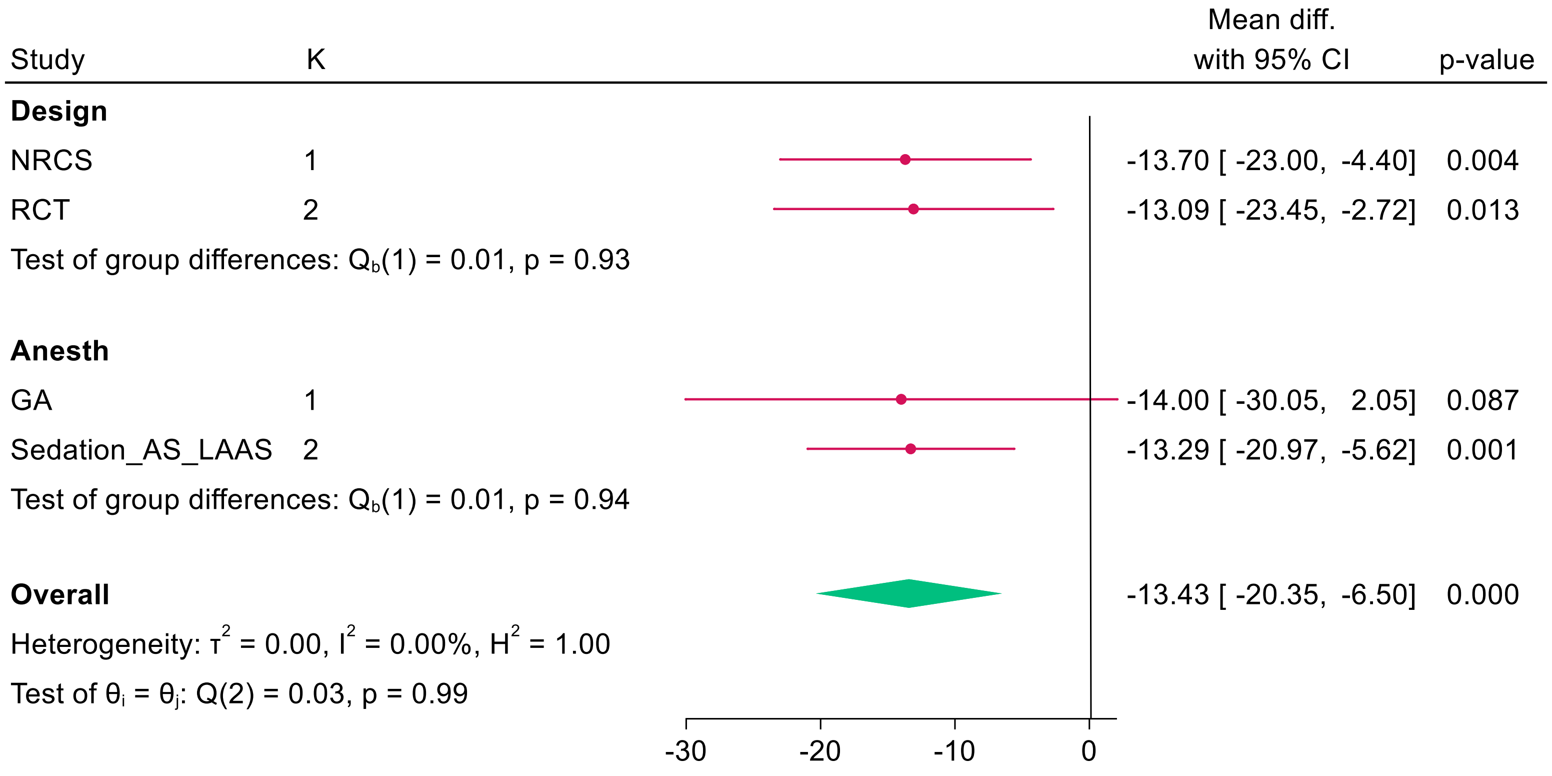

Random-effects DerSimonian–Laird model

NRCS: Non-randomized controlled studies; RCT: Randomized controlled trials; Anesth: type of anaesthesia; AS: Analgosedation; LAAS: Local anaesthesia with analgosedation

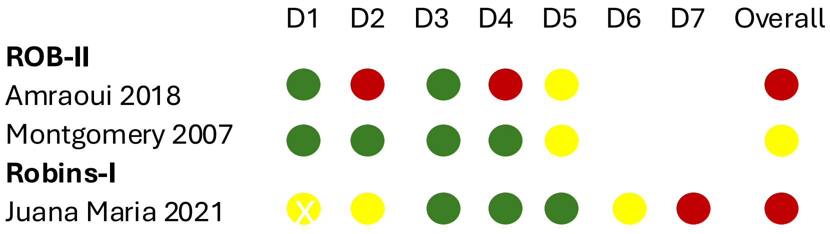

ROB-II : D1-Bias arising from the randomisation process; D2-Bias due to deviations from intended intervention; D3-Bias due to missing outcome data; D4-Bias in Measurement of the outcome; D5- Bias in the selection of the reported results

Robins-I: D1-Bais due to confounding; D2-Bias due to selection of participants; D3-Bias in classification of interventions; D4- Bias due to deviations from intended intervention; D5- Bias due to missing data; D6- Bias due to measurement of outcomes; D7- Bias in the selection of the reported results

Risk of Bias: High ● Some concern ● Low ●

Hospital stay (days)

Study	K	Mean Diff. with 95% CI	P-value
**Design**			
NRCS	1	-0.40 [-1.46, 0.66]	0.460
RCT	1	-0.80 [-1.91, 0.31]	0.158
Test of group differences: $Q_b(1) = 0.26$, p = 0.61			
**Anesth**			
GA	2	-0.59 [-1.36, 0.18]	0.131
Test of group differences: $Q_b(0) = 0.00$, p = .			
**Overall**		-0.59 [-1.36, 0.18]	0.131
Heterogeneity: $\tau^2 = 0.00$, $I^2 = 0.00\%$, $H^2 = 1.00$			
Test of $\theta_i = \theta_j$: Q(1) = 0.26, p = 0.61			

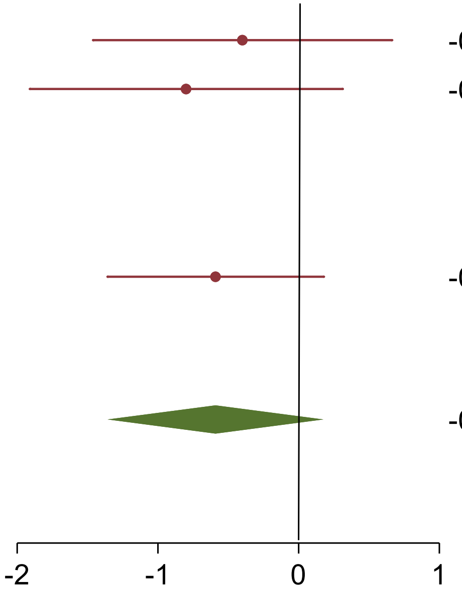

Random-effects DerSimonian-Laird model

NRCS: Non-randomized controlled studies; RCT: Randomized controlled trials; Anesth: type of anaesthesia; GA: General Anaesthesia

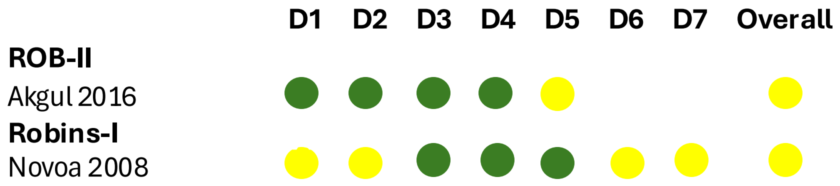

ROB-II : D1-Bias arising from the randomisation process; D2-Bias due to deviations from intended intervention; D3-Bias due to missing outcome data; D4-Bias in Measurement of the outcome; D5- Bias in the selection of the reported results
Robins-I: D1-Bais due to confounding; D2-Bias due to selection of participants; D3-Bias in classification of interventions; D4- Bias due to deviations from intended intervention; D5- Bias due to missing data; D6- Bias due to measurement of outcomes; D7- Bias in the selection of the reported results

Risk of Bias: High Some concern Low

Per-intervention hypnosis

Per-intervention hypnosis

Propofol consumption (mg)

Study	K	Mean diff. with 95% CI	p-value
**Design**			
NRCS	3	-155.92 [-240.38, -71.45]	0.000
RCT	1	2.80 [-10.72, 16.32]	0.685
Test of group differences: $Q_b(1) = 13.22$, p = 0.00			
**Anesth**			
GA	2	-196.18 [-594.75, 202.38]	0.335
Sedation_AS_LAAS	2	-85.09 [-100.56, -69.62]	0.000
Test of group differences: $Q_b(1) = 0.30$, p = 0.59			
**Overall**		-112.32 [-186.59, -38.05]	0.003
Heterogeneity: $\tau^2 = 5019.91$, $I^2 = 97.21\%$, $H^2 = 35.82$			
Test of $\theta_i = \theta_j$: Q(3) = 107.45, p = 0.00			

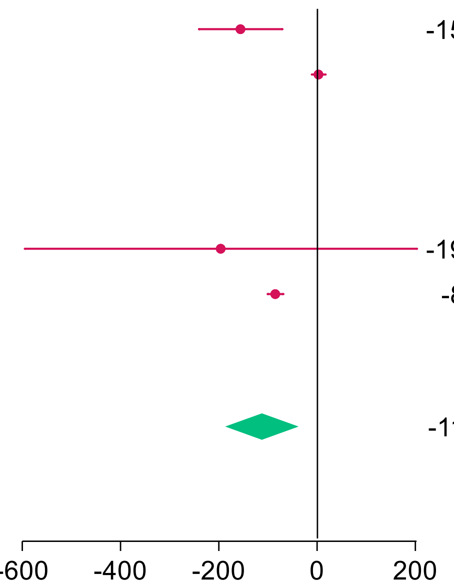

Random-effects DerSimonian–Laird model

NRCS: Non-randomized controlled studies; RCT: Randomized controlled trials; Anesth: type of anaesthesia; GA: General Anaesthesia; AS: Analgosedation; LAAS: Local anaesthesia with analgosedation

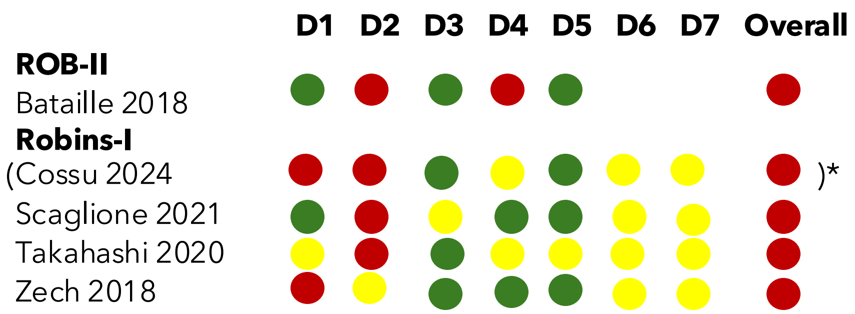

ROB-II : D1-Bias arising from the randomisation process; D2-Bias due to deviations from intended intervention; D3-Bias due to missing outcome data; D4-Bias in Measurement of the outcome; D5- Bias in the selection of the reported results

Robins-I: D1-Bais due to confounding; D2-Bias due to selection of participants; D3-Bias in classification of interventions; D4- Bias due to deviations from intended intervention; D5- Bias due to missing data; D6- Bias due to measurement of outcomes; D7- Bias in the selection of the reported results

Risk of Bias: High 🔴 Some concern 🟡 Low 🟢

*excluded due to very high risk of bias.

Per-intervention hypnosis

Any opioid, during the intervention

Study	K	Hedges's g with 95% CI	p-value
**Design**			
NRCS	3	-3.13 [-7.02, 0.77]	0.115
RCT	1	-1.45 [-1.87, -1.04]	0.000
Test of group differences: $Q_b(1) = 0.70$, $p = 0.40$			
**Anesth**			
GA	1	-143.36 [-174.02, -112.69]	0.000
LA	1	-1.45 [-1.87, -1.04]	0.000
Sedation_AS_LAAS	2	-0.79 [-1.14, -0.45]	0.000
Test of group differences: $Q_b(2) = 88.49$, $p = 0.00$			
**Overall**		-1.61 [-3.47, 0.26]	0.091
Heterogeneity: $\tau^2 = 2.64$, $I^2 = 96.64\%$, $H^2 = 29.72$			
Test of $\theta_i = \theta_j$: $Q(3) = 89.17$, $p = 0.00$			

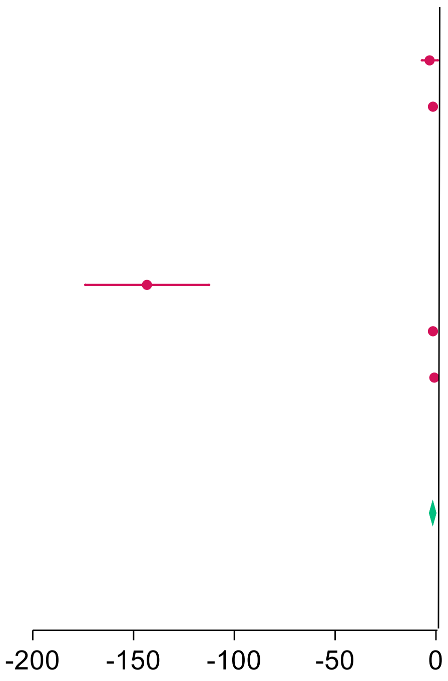

Random-effects DerSimonian–Laird model

NRCS: Non-randomized controlled studies; RCT: Randomized controlled trials; Anesth: type of anaesthesia; GA: General anaesthesia; LA: Local anaesthesia; AS: Analgosedation; LAAS: Local anaesthesia with analgosedation

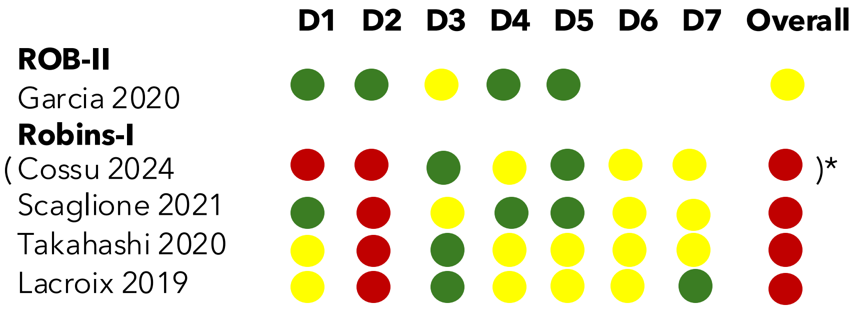

ROB-II : D1-Bias arising from the randomisation process; D2-Bias due to deviations from intended intervention; D3-Bias due to missing outcome data; D4-Bias in Measurement of the outcome; D5- Bias in the selection of the reported results
Robins-I: D1-Bais due to confounding; D2-Bias due to selection of participants; D3-Bias in classification of interventions; D4- Bias due to deviations from intended intervention; D5- Bias due to missing data; D6- Bias due to measurement of outcomes; D7- Bias in the selection of the reported results

Risk of Bias: High ● Some concern ● Low ●

*excluded due to very high risk of bias.

No need for analgosedation

Study	K	Risk ratio with 95% CI	p-value
**Design**			
NRCS	1	4.09 [1.35, 12.38]	0.013
RCT	2	3.74 [1.06, 13.18]	0.040
Test of group differences: $Q_b(1) = 0.01$, $p = 0.92$			
**Anesth**			
LA	1	10.50 [1.56, 70.89]	0.016
Sedation_AS_LAAS	2	2.65 [1.81, 3.88]	0.000
Test of group differences: $Q_b(1) = 1.92$, $p = 0.17$			
**Overall**		3.13 [1.77, 5.55]	0.000
Heterogeneity: $\tau^2 = 0.08$, $I^2 = 22.44\%$, $H^2 = 1.29$			
Test of $\theta_i = \theta_j$: $Q(2) = 2.58$, $p = 0.28$			

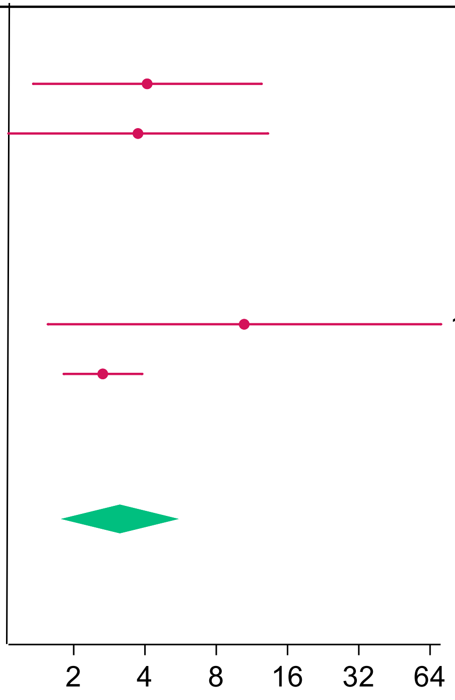

Random-effects DerSimonian–Laird model

NRCS: Non-randomized controlled studies; RCT: Randomized controlled trials; Anesth: type of anaesthesia; LA: Local anaesthesia; AS: Analgosedation; LAAS: Local anaesthesia with analgosedation

	D1	D2	D3	D4	D5	D6	D7	Overall
**ROB-II**								
Marc 2008	Low	High	Some concern	Some concern	Some concern			High
Lang 1996	Some concern	Some concern	Low	Some concern	Some concern			Some concern
**Robins-I**								
Elkins 2006	Low	High	Low	Low	Low	Some concern	Some concern	High

ROB-II : D1-Bias arising from the randomisation process; D2-Bias due to deviations from intended intervention; D3-Bias due to missing outcome data; D4-Bias in Measurement of the outcome; D5- Bias in the selection of the reported results
Robins-I: D1-Bais due to confounding; D2-Bias due to selection of participants; D3-Bias in classification of interventions; D4- Bias due to deviations from intended intervention; D5- Bias due to missing data; D6- Bias due to measurement of outcomes; D7- Bias in the selection of the reported results

Risk of Bias: High ● Some concern ● Low ●

Per-intervention hypnosis

Pain intensity during the intervention (VAS cm)

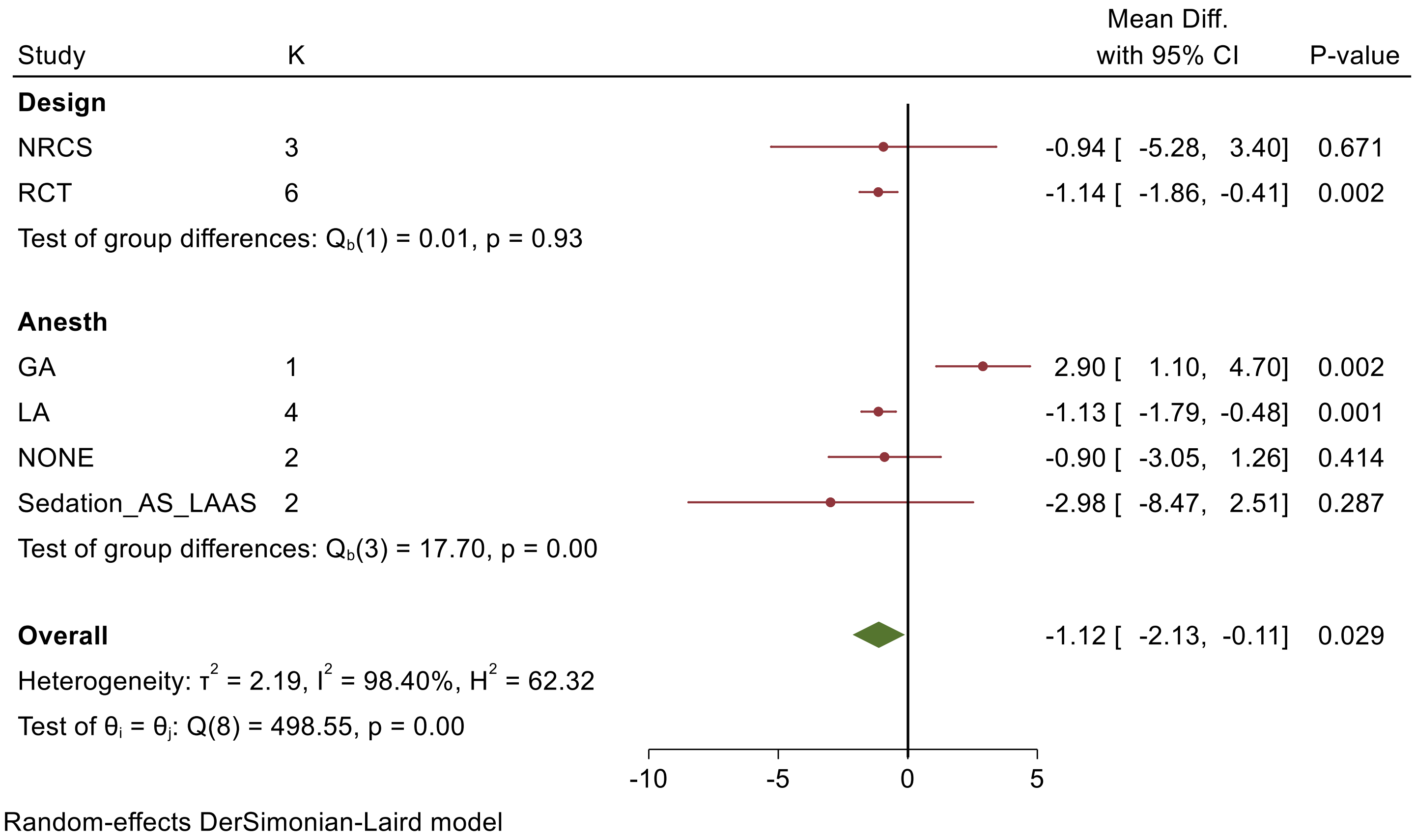

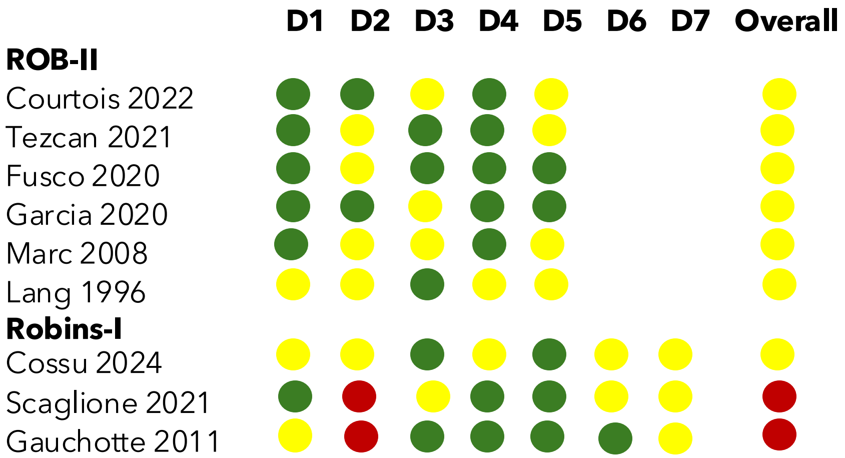

NRCS: Non-randomized controlled studies; RCT: Randomized controlled trials; Anesth: type of anaesthesia; GA: General anaesthesia; LA: Local anaesthesia; NONE: no anaesthesia

ROB-II : D1-Bias arising from the randomisation process; D2-Bias due to deviations from intended intervention; D3-Bias due to missing outcome data; D4-Bias in Measurement of the outcome; D5- Bias in the selection of the reported results

Robins-I: D1-Bais due to confounding; D2-Bias due to selection of participants; D3-Bias in classification of interventions; D4- Bias due to deviations from intended intervention; D5- Bias due to missing data; D6- Bias due to measurement of outcomes; D7- Bias in the selection of the reported results

Risk of Bias: High 🔴 Some concern 🟡 Low 🟢

Per-intervention hypnosis

Pain intensity after the intervention (VAS cm)

Study	K	Mean Diff. with 95% CI	P-value
**Design**			
NRCS	2	-1.00 [-1.39, -0.61]	0.000
RCT	7	-0.51 [-1.46, 0.43]	0.285
Test of group differences: $Q_b(1) = 0.86$, $p = 0.35$			
**Anesth**			
GA	2	-0.47 [-1.24, 0.31]	0.238
LA	4	-0.38 [-2.07, 1.31]	0.661
NONE	1	-2.00 [-2.62, -1.38]	0.000
Sedation_AS_LAAS	2	-0.72 [-1.70, 0.27]	0.156
Test of group differences: $Q_b(3) = 11.77$, $p = 0.01$			
**Overall**		-0.61 [-1.03, -0.20]	0.004
Heterogeneity: $\tau^2 = 0.26$, $I^2 = 92.23\%$, $H^2 = 12.87$			
Test of $\theta_i = \theta_j$: $Q(8) = 102.99$, $p = 0.00$			

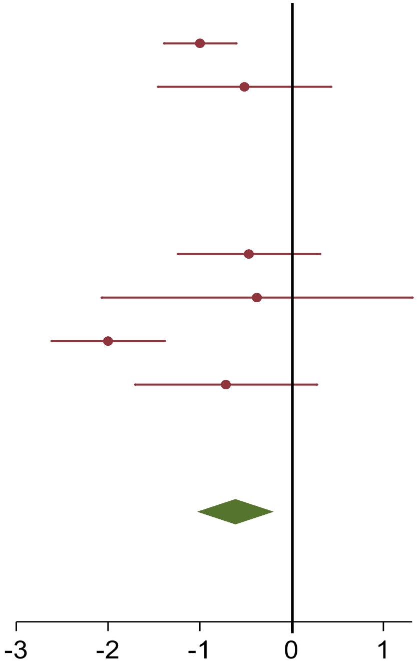

Random-effects DerSimonian-Laird model

NRCS: Non-randomized controlled studies; RCT: Randomized controlled trials; Anesth: type of anaesthesia; GA: General anaesthesia; LA: Local anaesthesia; NONE: no anaesthesia; AS: Analgosedation; LAAS: Local anaesthesia with analgosedation

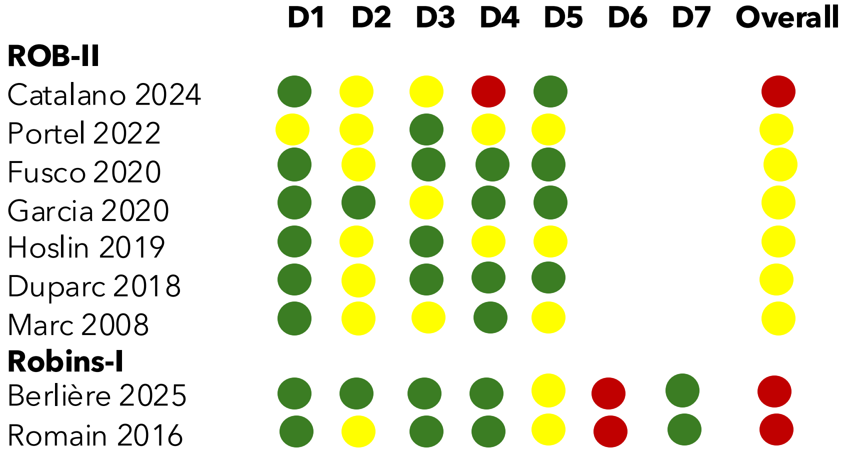

ROB-II : D1-Bias arising from the randomisation process; D2-Bias due to deviations from intended intervention; D3-Bias due to missing outcome data; D4-Bias in Measurement of the outcome; D5- Bias in the selection of the reported results

Robins-I: D1-Bais due to confounding; D2-Bias due to selection of participants; D3-Bias in classification of interventions; D4- Bias due to deviations from intended intervention; D5- Bias due to missing data; D6- Bias due to measurement of outcomes; D7- Bias in the selection of the reported results

Risk of Bias: High Some concern Low

Per-intervention hypnosis

Need for class I-II analgesic

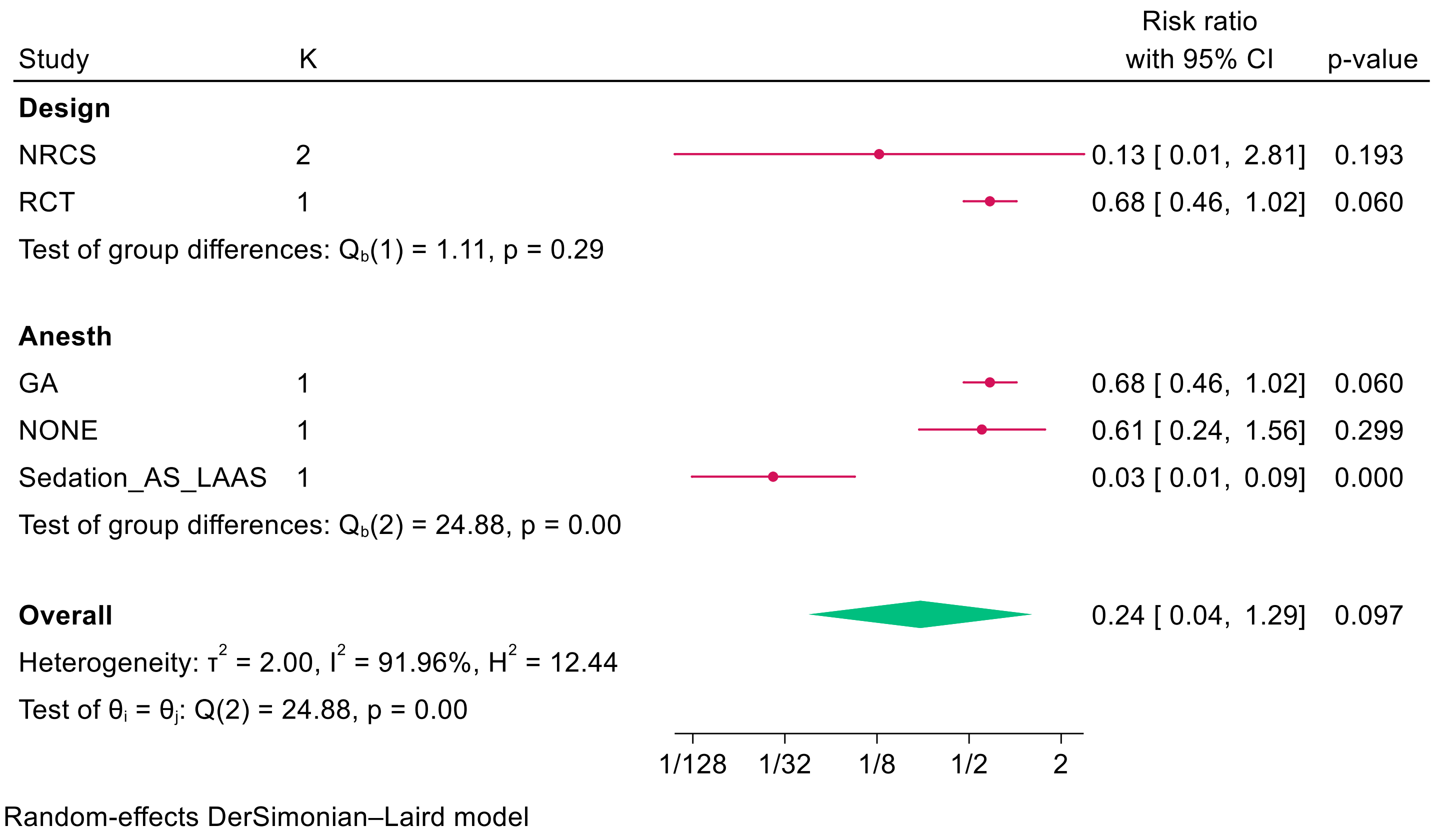

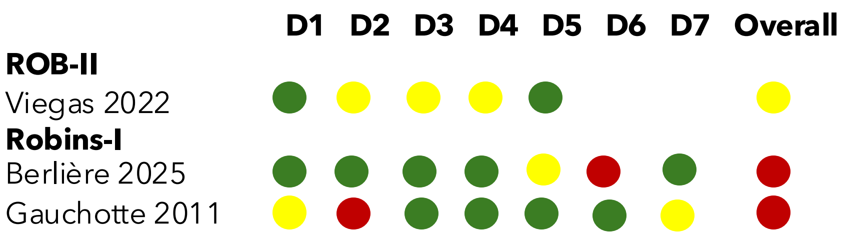

ROB-II : D1-Bias arising from the randomisation process; D2-Bias due to deviations from intended intervention; D3-Bias due to missing outcome data; D4-Bias in Measurement of the outcome; D5- Bias in the selection of the reported results
Robins-I: D1-Bais due to confounding; D2-Bias due to selection of participants; D3-Bias in classification of interventions; D4- Bias due to deviations from intended intervention; D5- Bias due to missing data; D6- Bias due to measurement of outcomes; D7- Bias in the selection of the reported results

Risk of Bias: High ● Some concern ● Low ●

NRCS: Non-randomized controlled studies; RCT: Randomized controlled trials; Anesth: type of anaesthesia; GA: General anaesthesia; NONE: no anaesthesia; AS: Analgosedation; LAAS: Local anaesthesia with analgo sedation

Per-intervention hypnosis

Need for class III analgesic

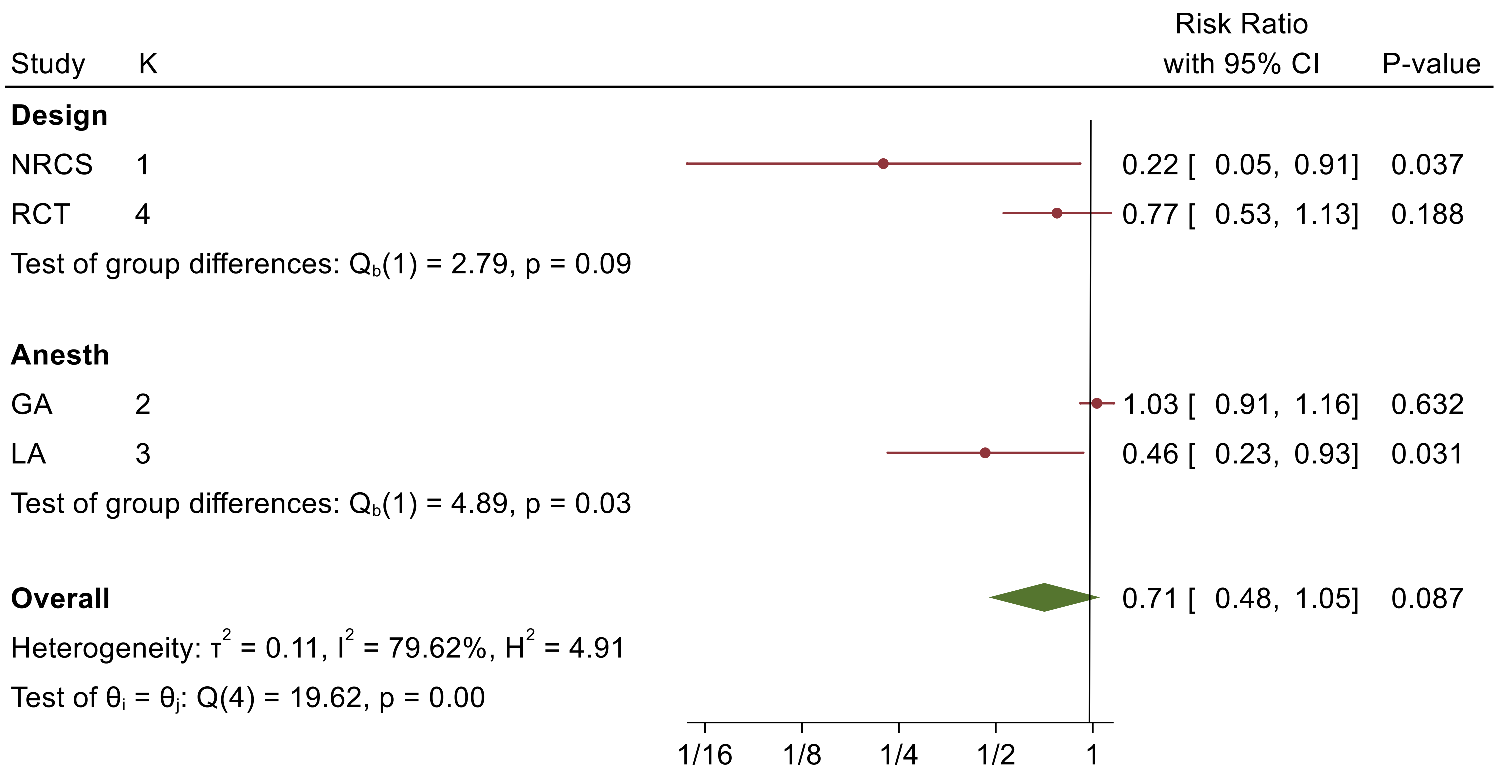

Random-effects DerSimonian-Laird model

NRCS: Non-randomized controlled studies; RCT: Randomized controlled trials; Anesth: type of anaesthesia; GA: General anaesthesia; LA: Local anaesthesia;

	D1	D2	D3	D4	D5	D6	D7	Overall
**ROB-II**								
Viegas 2022	Low	Some concern	Some concern	Some concern	Low			Some concern
Garcia 2020	Low	Low	Some concern	Low	Low			Some concern
Duparc-Alegria 2018	Low	Low	Low	Low	Low			Low
Weinstein 1991	Some concern	Low	Low	Some concern	Some concern			Some concern
**Robins-I**								
Touzé 2020	Some concern	Some concern	Low	Some concern	Some concern	Some concern	Low	Some concern

ROB-II : D1-Bias arising from the randomisation process; D2-Bias due to deviations from intended intervention; D3-Bias due to missing outcome data; D4-Bias in Measurement of the outcome; D5- Bias in the selection of the reported results

Robins-I: D1-Bais due to confounding; D2-Bias due to selection of participants; D3-Bias in classification of interventions; D4- Bias due to deviations from intended intervention; D5- Bias due to missing data; D6- Bias due to measurement of outcomes; D7- Bias in the selection of the reported results

Risk of Bias: High ● Some concern ● Low ●

Anxiety during the intervention (score)

Study	K	Hedges's g with 95% CI	p-value
**Design**			
NRCS	1	-1.18 [-1.96, -0.40]	0.003
RCT	2	-0.11 [-0.59, 0.37]	0.641
Test of group differences: $Q_b(1) = 5.20$, $p = 0.02$			
**Anesth**			
LA	1	-0.49 [-1.20, 0.21]	0.172
Sedation_AS_LAAS	2	-0.51 [-1.70, 0.69]	0.404
Test of group differences: $Q_b(1) = 0.00$, $p = 0.98$			
**Overall**		-0.47 [-1.21, 0.26]	0.209
Heterogeneity: $\tau^2 = 0.33$, $I^2 = 80.51\%$, $H^2 = 5.13$			
Test of $\theta_i = \theta_j$: $Q(2) = 10.26$, $p = 0.01$			

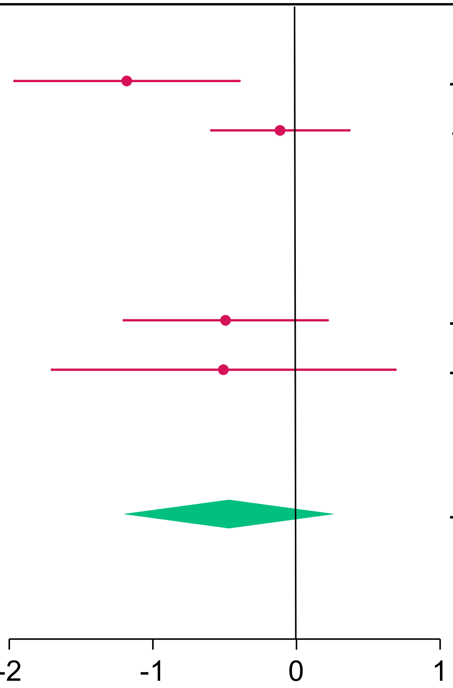

Random-effects DerSimonian–Laird model

NRCS: Non-randomized controlled studies; RCT: Randomized controlled trials; Anesth: type of anaesthesia; LA: Local anaesthesia; NONE: no anaesthesia; SC: Standard care; AS: Analgosedation; LAAS: Local anaesthesia with analgosedation

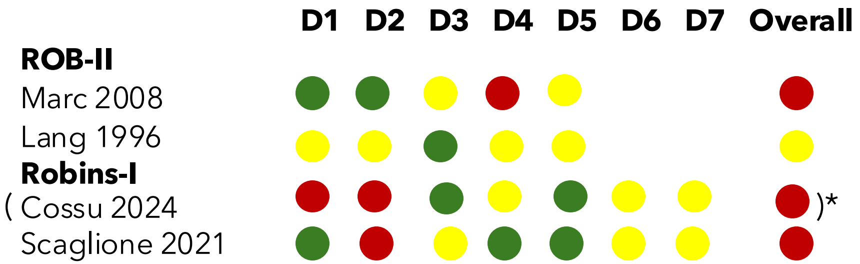

ROB-II : D1-Bias arising from the randomisation process; D2-Bias due to deviations from intended intervention; D3-Bias due to missing outcome data; D4-Bias in Measurement of the outcome; D5- Bias in the selection of the reported results

Robins-I: D1-Bais due to confounding; D2-Bias due to selection of participants; D3-Bias in classification of interventions; D4- Bias due to deviations from intended intervention; D5- Bias due to missing data; D6- Bias due to measurement of outcomes; D7- Bias in the selection of the reported results

Risk of Bias: High ● Some concern ● Low ●

*excluded due to very high risk of bias.

Anxiety after the intervention (score)

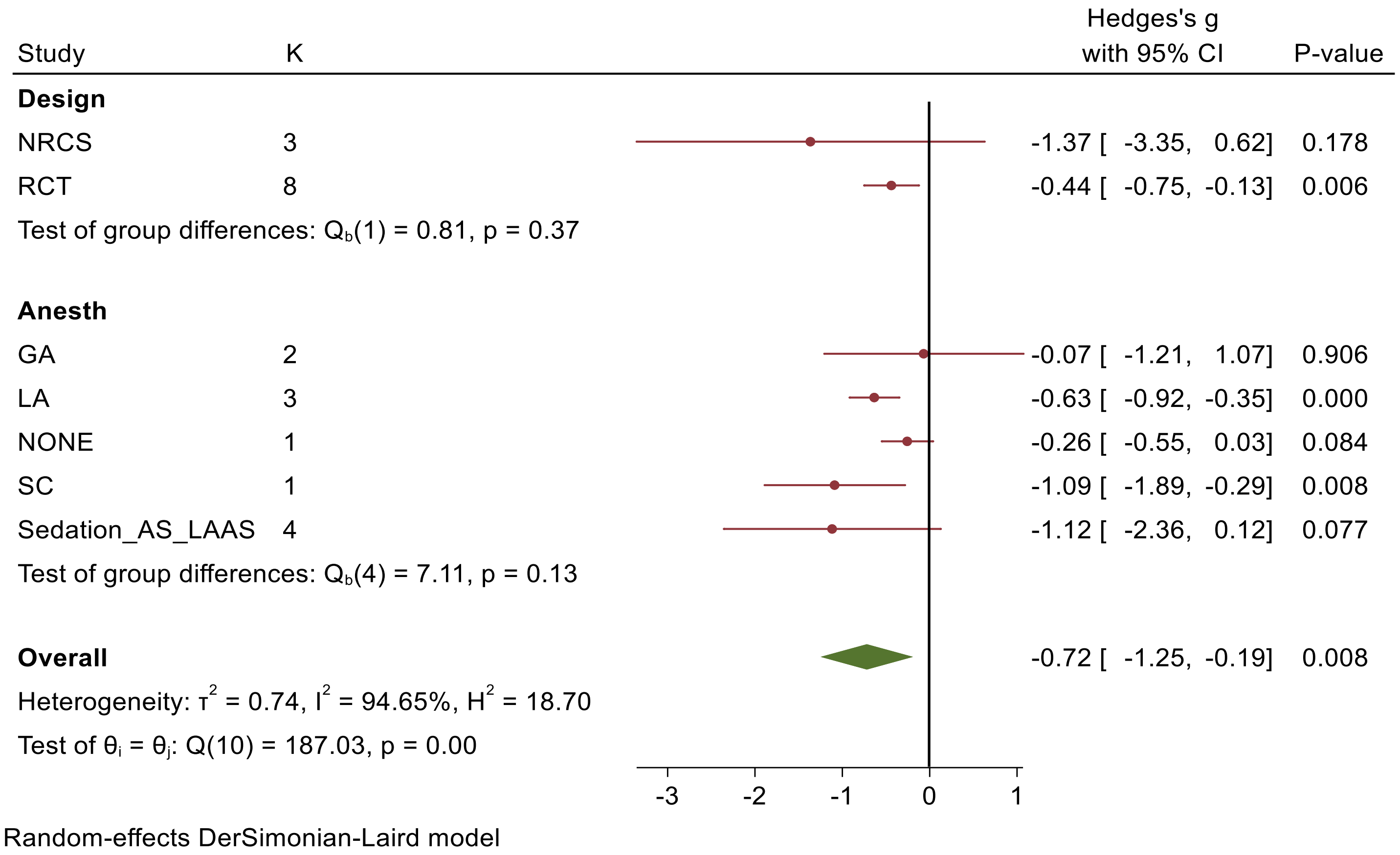

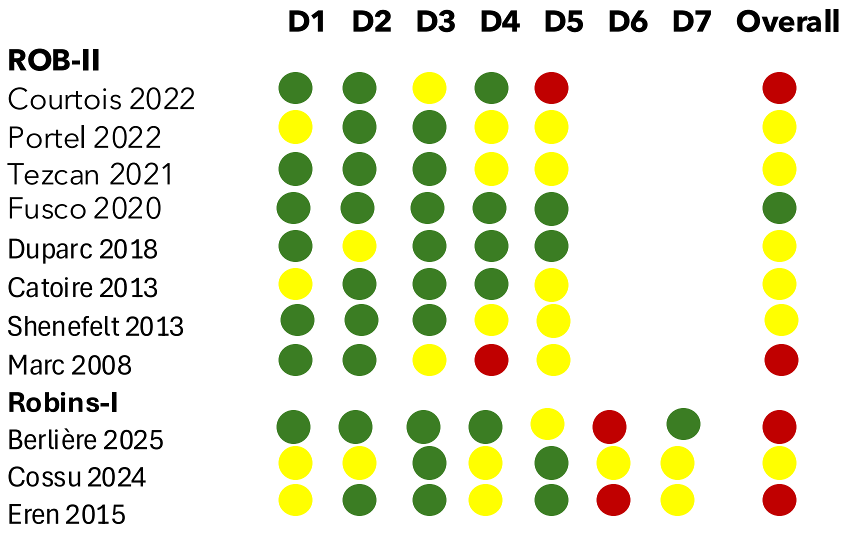

NRCS: Non-randomized controlled studies; RCT: Randomized controlled trials; Anesth: type of anaesthesia; GA: General anaesthesia; LA: Local anaesthesia; NONE: no anaesthesia; SC: Standard care; AS: Analgosedation; LAAS: Local anaesthesia with analgosedation

ROB-II : D1-Bias arising from the randomisation process; D2-Bias due to deviations from intended intervention; D3-Bias due to missing outcome data; D4-Bias in Measurement of the outcome; D5- Bias in the selection of the reported results
Robins-I: D1-Bais due to confounding; D2-Bias due to selection of participants; D3-Bias in classification of interventions; D4- Bias due to deviations from intended intervention; D5- Bias due to missing data; D6- Bias due to measurement of outcomes; D7- Bias in the selection of the reported results

Risk of Bias: High Some concern Low

Per-intervention hypnosis

Patient satisfaction (VAS cm)

Study	K	Mean Diff. with 95% CI	P-value
**Design**			
NRCS	2	0.15 [-1.22, 1.52]	0.826
RCT	4	0.27 [-0.18, 0.72]	0.235
Test of group differences: $Q_b(1) = 0.03$, p = 0.87			
**Anesth**			
GA	2	-0.49 [-0.63, -0.34]	0.000
LA	2	0.22 [-0.36, 0.81]	0.455
NONE	1	0.80 [0.23, 1.37]	0.006
Sedation_AS_LAAS	1	0.90 [0.18, 1.62]	0.014
Test of group differences: $Q_b(3) = 34.22$, p = 0.00			
**Overall**		0.21 [-0.34, 0.75]	0.461
Heterogeneity: $\tau^2 = 0.39$, $I^2 = 88.96\%$, $H^2 = 9.06$			
Test of $\theta_i = \theta_j$: Q(5) = 45.28, p = 0.00			

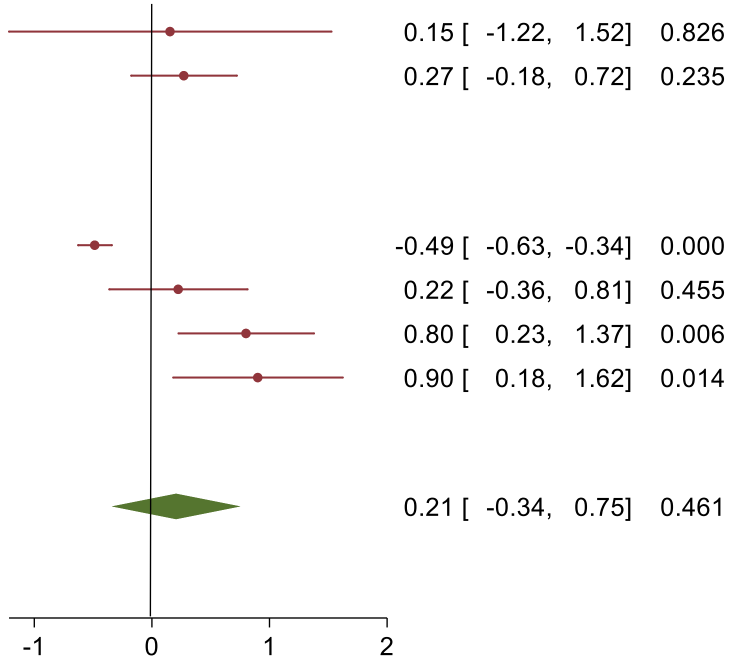

Random-effects DerSimonian-Laird model

NRCS: Non-randomized controlled studies; RCT: Randomized controlled trials; Anesth: type of anaesthesia; GA: General anaesthesia; LA: Local anaesthesia; NONE: no anaesthesia; AS: Analgosedation; LAAS: Local anaesthesia with analgosedation

	D1	D2	D3	D4	D5	D6	D7	Overall
**ROB-II**								
Polomeni 2024	Low	Low	Some concern	Low	Low			Some concern
Fusco 2020	Low	Low	Low	Low	Low			Low
Defechereux 2000	Low	Low	Low	Some concern	Some concern			Some concern
Hoslin 2019	Low	Some concern	Low	Some concern	Some concern			Some concern
**Robins-I**								
Romain 2016	Low	Some concern	Low	Low	Some concern	High	Low	High
Eren 2015	Some concern	Low	Low	Some concern	Low	High	Some concern	High

ROB-II : D1-Bias arising from the randomisation process; D2-Bias due to deviations from intended intervention; D3-Bias due to missing outcome data; D4-Bias in Measurement of the outcome; D5- Bias in the selection of the reported results

Robins-I: D1-Bais due to confounding; D2-Bias due to selection of participants; D3-Bias in classification of interventions; D4- Bias due to deviations from intended intervention; D5- Bias due to missing data; D6- Bias due to measurement of outcomes; D7- Bias in the selection of the reported results

Risk of Bias: High (red) Some concern (yellow) Low (green)

Per-intervention hypnosis

Medical team satisfaction (VAS cm)

Study	K	Mean diff. with 95% CI	p-value
**Design**			
NRCS	2	1.06 [-0.31, 2.43]	0.130
RCT	2	1.20 [1.09, 1.31]	0.000
Test of group differences: $Q_b(1) = 0.04$, $p = 0.84$			
**Anesth**			
GA	1	0.40 [0.32, 0.48]	0.000
LA	1	1.20 [0.80, 1.60]	0.000
Sedation_AS_LAAS	2	1.40 [0.85, 1.96]	0.000
Test of group differences: $Q_b(2) = 26.27$, $p = 0.00$			
**Overall**		1.10 [0.52, 1.68]	0.000
Heterogeneity: $\tau^2 = 0.31$, $I^2 = 97.86\%$, $H^2 = 46.70$			
Test of $\theta_i = \theta_j$: $Q(3) = 140.10$, $p = 0.00$			

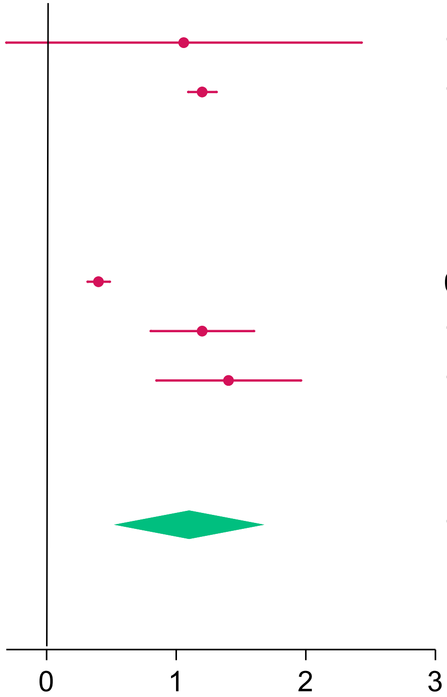

Random-effects DerSimonian–Laird model

NRCS: Non-randomized controlled studies; RCT: Randomized controlled trials; Anesth: type of anaesthesia; GA: General anaesthesia; LA: Local anaesthesia AS: Analgosedation; LAAS: Local anaesthesia with analgosedation

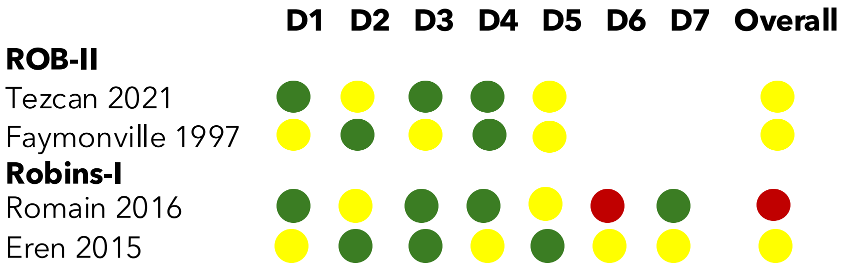

ROB-II : D1-Bias arising from the randomisation process; D2-Bias due to deviations from intended intervention; D3-Bias due to missing outcome data; D4-Bias in Measurement of the outcome; D5- Bias in the selection of the reported results

Robins-I: D1-Bais due to confounding; D2-Bias due to selection of participants; D3-Bias in classification of interventions; D4- Bias due to deviations from intended intervention; D5- Bias due to missing data; D6- Bias due to measurement of outcomes; D7- Bias in the selection of the reported results

Risk of Bias: High Some concern Low

Per-intervention hypnosis

Complications (yes/no)

Study	K	Risk ratio with 95% CI	p-value
**Design**			
NRCS	6	0.78 [0.53, 1.14]	0.202
RCT	1	0.67 [0.12, 3.77]	0.647
Test of group differences: $Q_b(1) = 0.03$, $p = 0.86$			
**Anesth**			
GA	4	0.48 [0.20, 1.13]	0.094
LA	1	0.70 [0.33, 1.46]	0.337
Sedation_AS_LAAS	2	0.95 [0.58, 1.57]	0.853
Test of group differences: $Q_b(2) = 1.96$, $p = 0.38$			
**Overall**		0.78 [0.53, 1.12]	0.179
Heterogeneity: $\tau^2 = 0.00$, $I^2 = 0.00\%$, $H^2 = 1.00$			
Test of $\theta_i = \theta_j$: $Q(6) = 2.40$, $p = 0.88$			

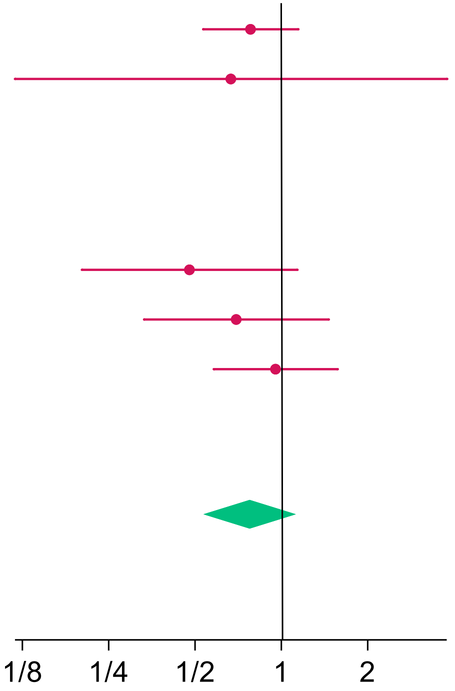

Random-effects DerSimonian–Laird model

NRCS: Non-randomized controlled studies; RCT: Randomized controlled trials; Anesth: type of anaesthesia; GA: General anaesthesia; LA: Local anaesthesia; AS: Analgosedation; LAAS: Local anaesthesia with analgosedation

	D1	D2	D3	D4	D5	D6	D7	Overall
**ROB-II**								
Viegas 2022	Low	Some concern	Some concern	Some concern	Low			Some concern
**Robins-I**								
Derycke 2024	Some concern	Some concern	Low	High	Low	Some concern	Some concern	High
Badidi 2021	Some concern	High	Low	Some concern	High	Some concern	Some concern	High
Pesce 2020	Some concern	High	Low	Some concern	Some concern	Some concern	Some concern	High
Takahashi 2020	Some concern	High	Low	Some concern	Some concern	Some concern	Some concern	Some concern
Touzé 2020	Some concern	Some concern	Low	Some concern	Some concern	Some concern	Low	Some concern
Berlière 2018	Some concern	Some concern	Low	High	Some concern	Some concern	Low	High

ROB-II : D1-Bias arising from the randomisation process; D2-Bias due to deviations from intended intervention; D3-Bias due to missing outcome data; D4-Bias in Measurement of the outcome; D5- Bias in the selection of the reported results
Robins-I: D1-Bais due to confounding; D2-Bias due to selection of participants; D3-Bias in classification of interventions; D4- Bias due to deviations from intended intervention; D5- Bias due to missing data; D6- Bias due to measurement of outcomes; D7- Bias in the selection of the reported results

Risk of Bias: High ● Some concern ● Low ●

Postoperative nausea and vomiting

Study	K	Risk Ratio with 95% CI	P-value
**Design**			
NRCS	4	0.14 [0.05, 0.43]	0.001
RCT	3	0.43 [0.25, 0.74]	0.002
Test of group differences: $Q_b(1) = 3.17$, $p = 0.08$			
**Anesth**			
GA	4	0.43 [0.26, 0.71]	0.001
LA	1	0.15 [0.02, 1.12]	0.064
Sedation_AS_LAAS	2	0.09 [0.02, 0.42]	0.002
Test of group differences: $Q_b(2) = 4.39$, $p = 0.11$			
**Overall**		0.25 [0.13, 0.49]	0.000
Heterogeneity: $\tau^2 = 0.39$, $I^2 = 49.47\%$, $H^2 = 1.98$			
Test of $\theta_i = \theta_j$: $Q(6) = 11.87$, $p = 0.06$			

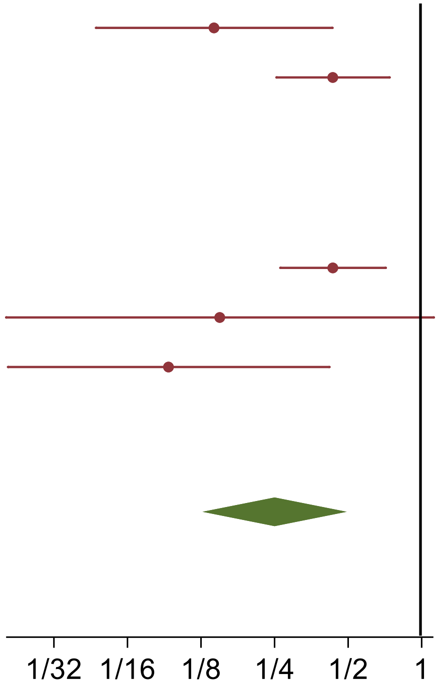

Random-effects DerSimonian-Laird model

NRCS: Non-randomized controlled studies; RCT: Randomized controlled trials; Anesth: type of anaesthesia; GA: General anaesthesia; LA: Local anaesthesia; AS: Analgosedation; LAAS: Local anaesthesia with analgosedation

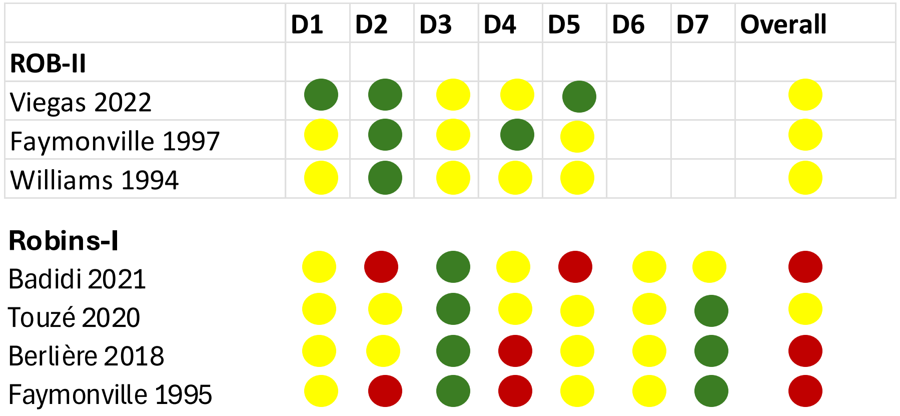

ROB-II : D1-Bias arising from the randomisation process; D2-Bias due to deviations from intended intervention; D3-Bias due to missing outcome data; D4-Bias in Measurement of the outcome; D5- Bias in the selection of the reported results

Robins-I: D1-Bais due to confounding; D2-Bias due to selection of participants; D3-Bias in classification of interventions; D4- Bias due to deviations from intended intervention; D5- Bias due to missing data; D6- Bias due to measurement of outcomes; D7- Bias in the selection of the reported results

Risk of Bias: High 🔴 Some concern 🟡 Low 🟢

Per-intervention hypnosis

Procedure duration (min)

Study	K	Mean Diff. with 95% CI	P-value
**Design**			
NRCS	10	2.66 [-3.23, 8.54]	0.376
RCT	7	-2.02 [-3.81, -0.23]	0.027
Test of group differences: $Q_b(1) = 2.22$, $p = 0.14$			
**Anesth**			
GA	7	-1.93 [-9.39, 5.52]	0.611
LA	4	-1.46 [-3.45, 0.52]	0.149
Sedation_AS_LAAS	6	0.53 [-1.71, 2.76]	0.645
Test of group differences: $Q_b(2) = 1.82$, $p = 0.40$			
**Overall**		-0.56 [-2.26, 1.15]	0.521

Heterogeneity: $\tau^2 = 4.53$, $I^2 = 76.35\%$, $H^2 = 4.23$

Test of $\theta_i = \theta_j$: $Q(16) = 67.66$, $p = 0.00$

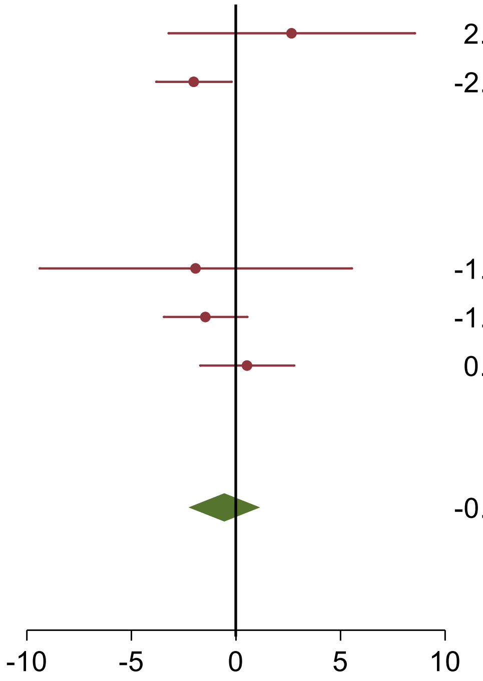

Random-effects DerSimonian-Laird model

NRCS: Non-randomized controlled studies; RCT: Randomized controlled trials; Anesth: type of anaesthesia; GA: General anaesthesia; LA: Local anaesthesia; AS: Analgosedation; LAAS: Local anaesthesia with analgosedation

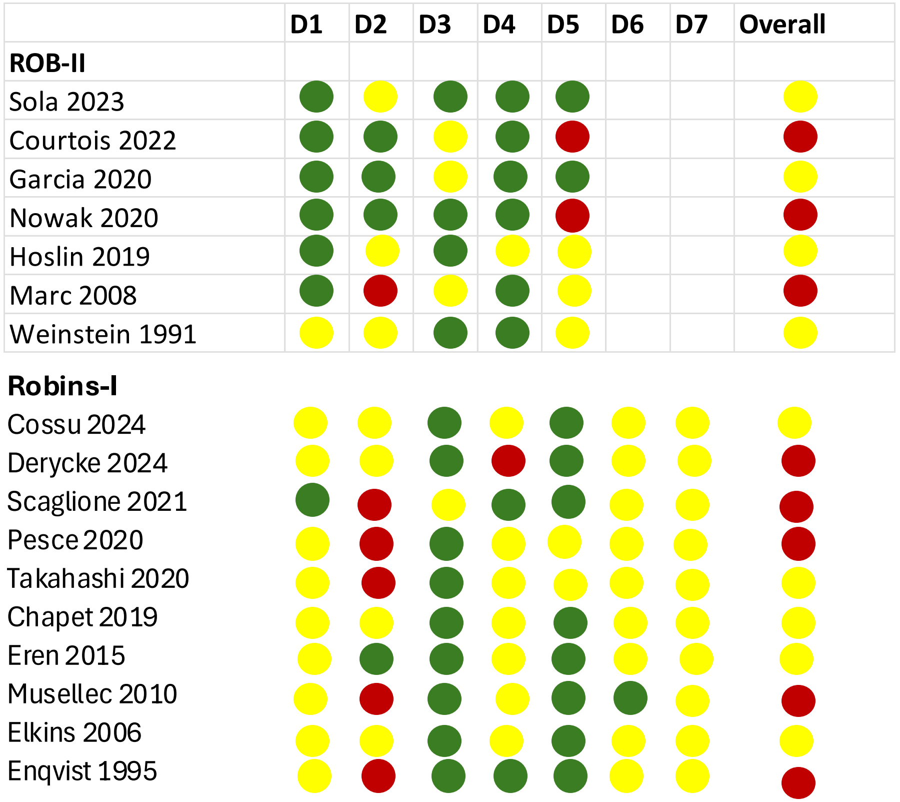

ROB-II : D1-Bias arising from the randomisation process; D2-Bias due to deviations from intended intervention; D3-Bias due to missing outcome data; D4-Bias in Measurement of the outcome; D5- Bias in the selection of the reported results

Robins-I: D1-Bais due to confounding; D2-Bias due to selection of participants; D3-Bias in classification of interventions; D4- Bias due to deviations from intended intervention; D5- Bias due to missing data; D6- Bias due to measurement of outcomes; D7- Bias in the selection of the reported results

Risk of Bias: High ● Some concern ● Low ●

Per-intervention hypnosis

Time in PACU (min)

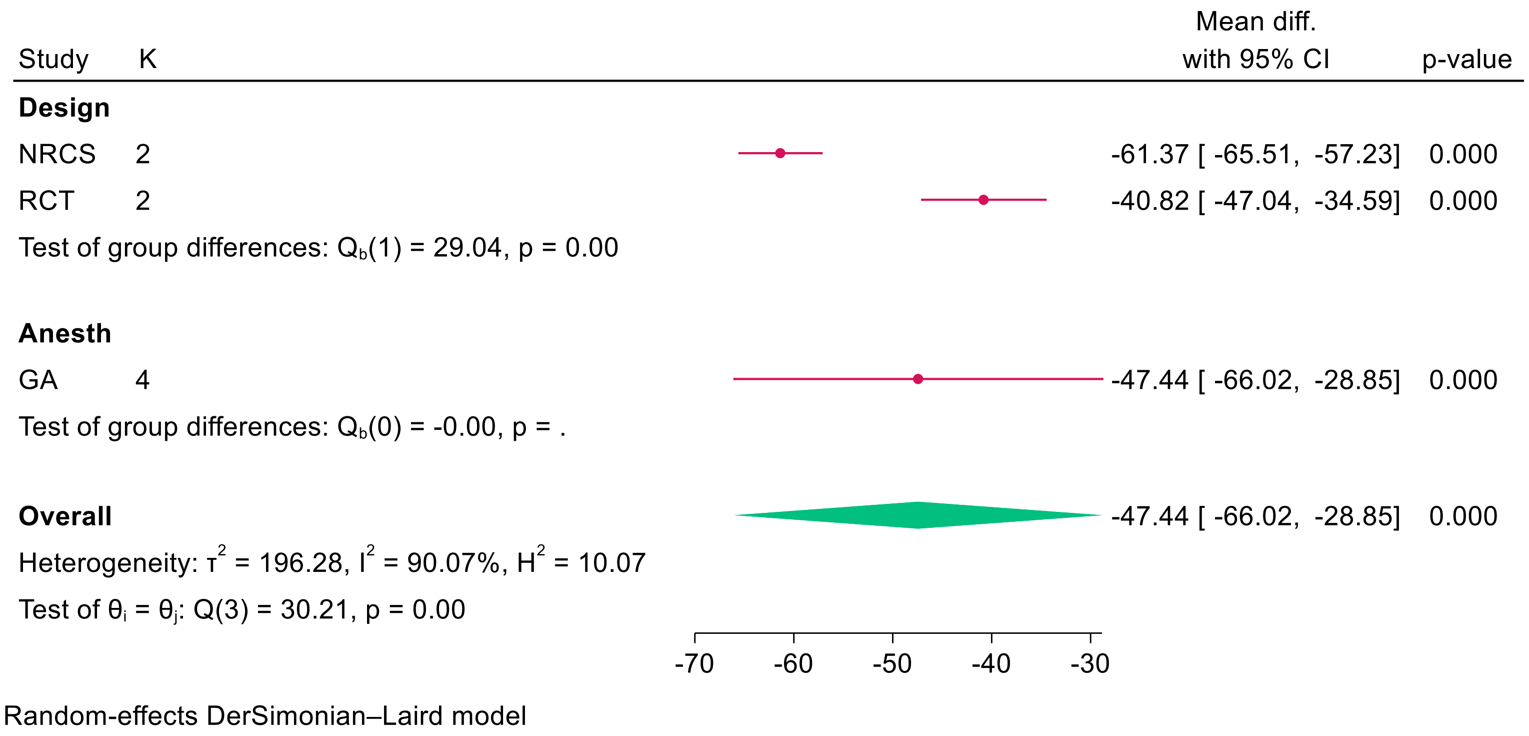

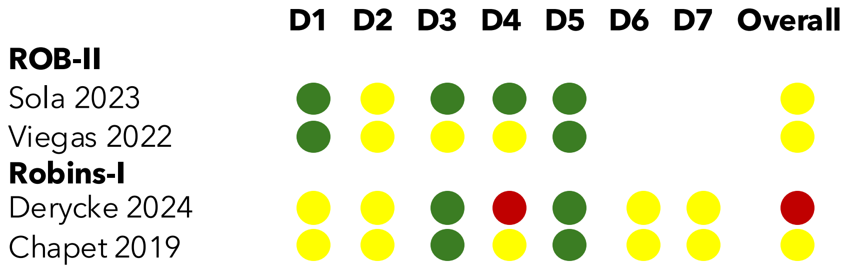

ROB-II : D1-Bias arising from the randomisation process; D2-Bias due to deviations from intended intervention; D3-Bias due to missing outcome data; D4-Bias in Measurement of the outcome; D5- Bias in the selection of the reported results
Robins-I: D1-Bais due to confounding; D2-Bias due to selection of participants; D3-Bias in classification of interventions; D4- Bias due to deviations from intended intervention; D5- Bias due to missing data; D6- Bias due to measurement of outcomes; D7- Bias in the selection of the reported results

Risk of Bias: High ● Some concern ● Low ●

NRCS: Non-randomized controlled studies; RCT: Randomized controlled trials; Anesth: type of anaesthesia; GA: General anaesthesia

Per-intervention hypnosis

Hospital stay (day)

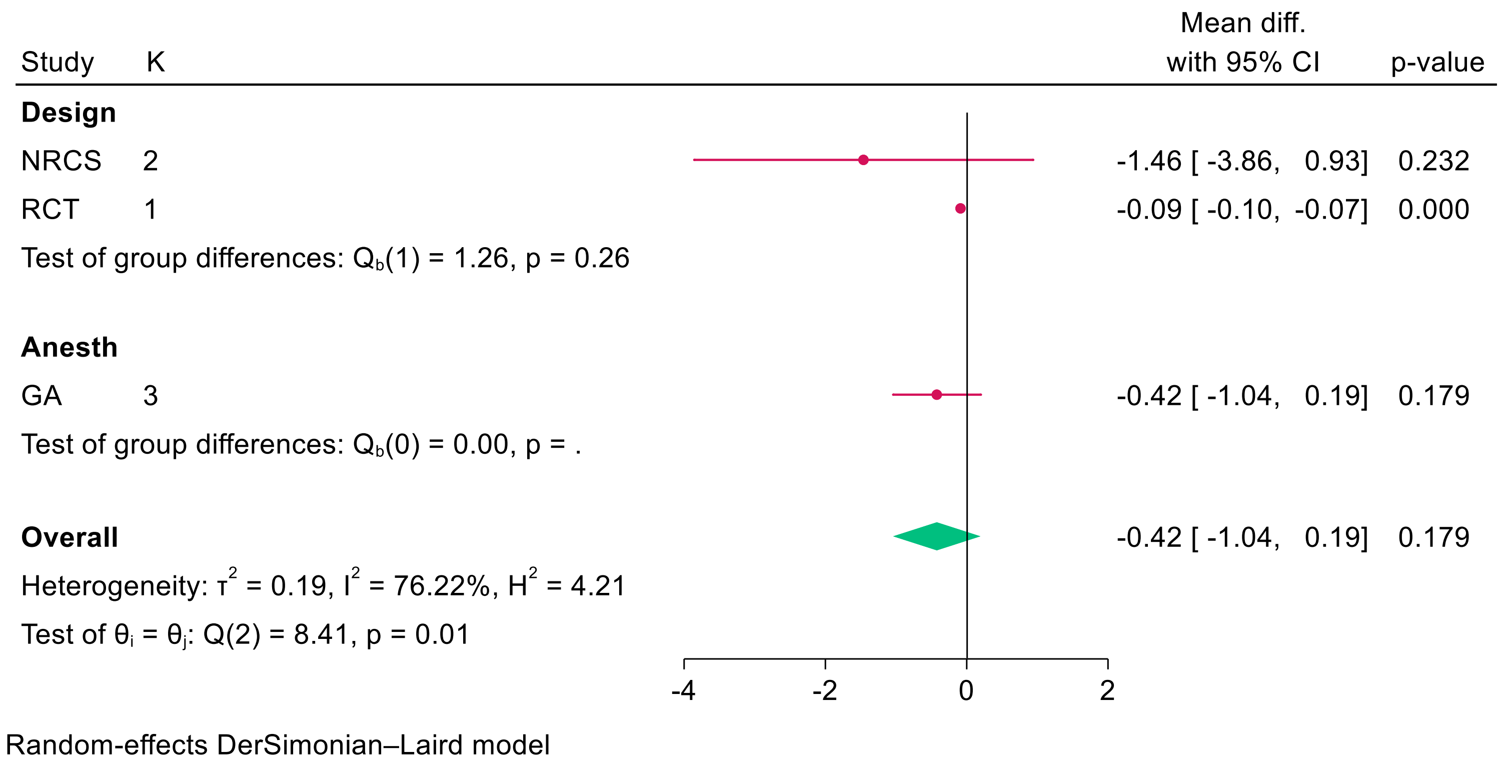

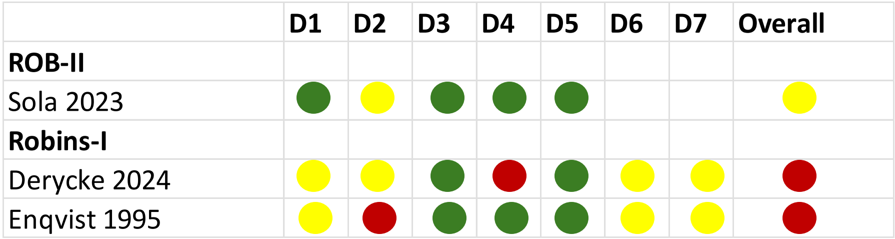

ROB-II : D1-Bias arising from the randomisation process; D2-Bias due to deviations from intended intervention; D3-Bias due to missing outcome data; D4-Bias in Measurement of the outcome; D5- Bias in the selection of the reported results
Robins-I: D1-Bais due to confounding; D2-Bias due to selection of participants; D3-Bias in classification of interventions; D4- Bias due to deviations from intended intervention; D5- Bias due to missing data; D6- Bias due to measurement of outcomes; D7- Bias in the selection of the reported results

Risk of Bias: High Some concern Low

NRCS: Non-randomized controlled studies; RCT: Randomized controlled trials; Anesth: type of anaesthesia; GA: General anaesthesia;

Post-intervention hypnosis

Post-intervention hypnosis

Pain intensity after the intervention (VAS cm)

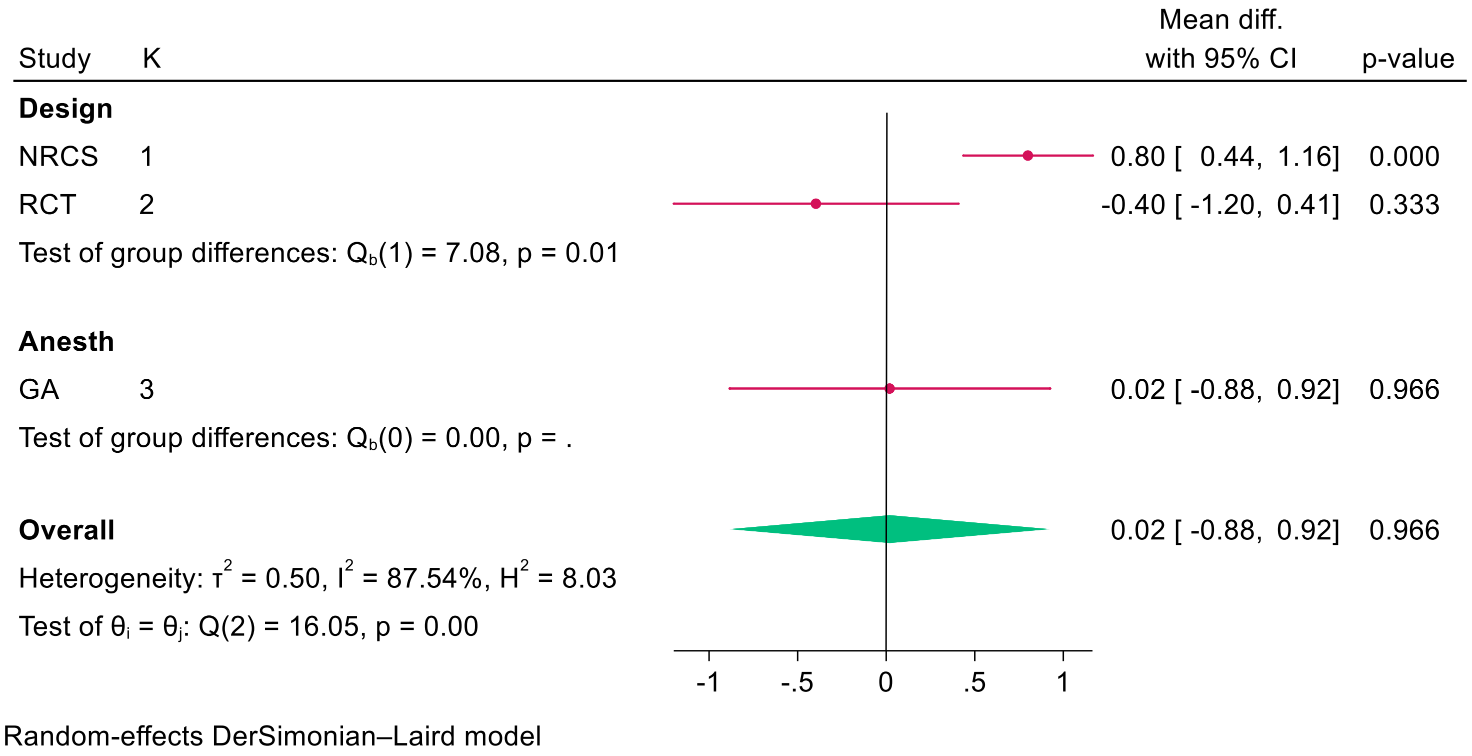

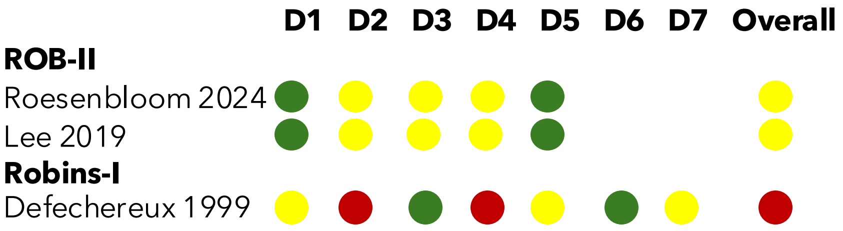

ROB-II : D1-Bias arising from the randomisation process; D2-Bias due to deviations from intended intervention; D3-Bias due to missing outcome data; D4-Bias in Measurement of the outcome; D5- Bias in the selection of the reported results
Robins-I: D1-Bais due to confounding; D2-Bias due to selection of participants; D3-Bias in classification of interventions; D4- Bias due to deviations from intended intervention; D5- Bias due to missing data; D6- Bias due to measurement of outcomes; D7- Bias in the selection of the reported results

Risk of Bias: High Some concern Low

NRCS: Non-randomized controlled studies; RCT: Randomized controlled trials; Anesth: type of anaesthesia; GA: General anaesthesia;
